# Allosteric activation of T cell antigen receptor signaling by quaternary structure relaxation

**DOI:** 10.1016/j.celrep.2021.109531

**Published:** 2021-08-10

**Authors:** Anna-Lisa Lanz, Giulia Masi, Nicla Porciello, André Cohnen, Deborah Cipria, Dheeraj Prakaash, Štefan Bálint, Roberto Raggiaschi, Donatella Galgano, David K. Cole, Marco Lepore, Omer Dushek, Michael L. Dustin, Mark S.P. Sansom, Antreas C. Kalli, Oreste Acuto

(Cell Reports *36*, 109375-1–109375-19.e1–e15; July 13, 2021)

In the originally published version of this article, p-MHC was incorrectly defined due to copyeditor oversight. The correct definition for p-MHC is “peptide-major histocompatibility complex,” and this has been corrected in the paper online.

The production team apologizes for the error.

