# Supplementary material for: Allosteric activation of T cell antigen receptor signaling by quaternary structure relaxation
Source: Cell Rep. 2021 Jul 13;36(2):109375. doi: 10.1016/j.celrep.2021.109375 (PMC8293630; doi:10.1016/j.celrep.2021.109375)
Supplement: Document S1. Figures S1–S7 and Tables S1–S4 [file mmc1.pdf]

Cell Reports, Volume *36*

Supplemental information

Allosteric activation of T cell antigen receptor signaling by quaternary structure relaxation

Anna-Lisa Lanz, Giulia Masi, Nicla Porciello, André Cohnen, Deborah Cipria, Dheeraj Prakaash, Štefan Bálint, Roberto Raggiaschi, Donatella Galgano, David K. Cole, Marco Lepore, Omer Dushek, Michael L. Dustin, Mark S.P. Sansom, Antreas C. Kalli, and Oreste Acuto

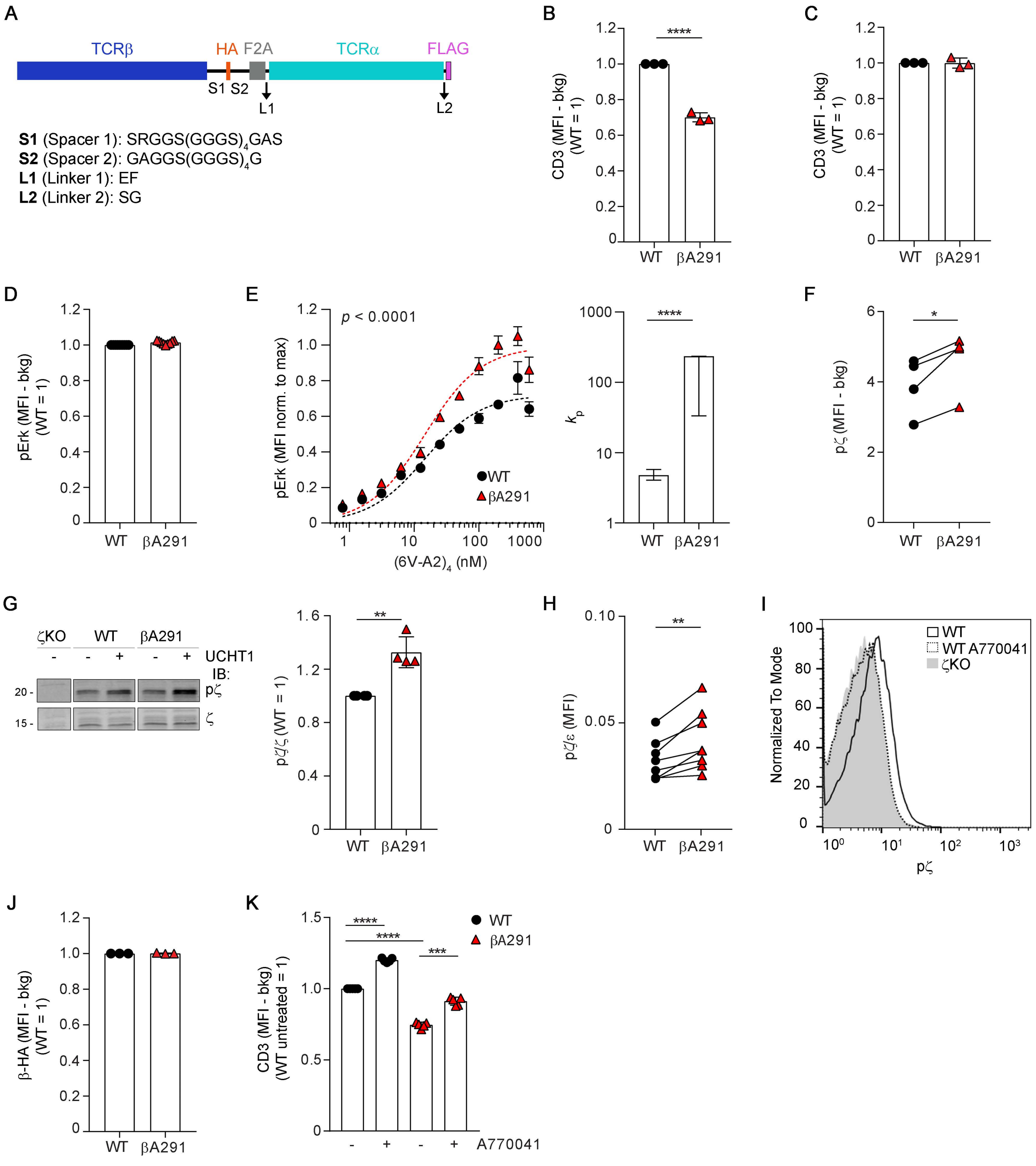

A
TCRβ
HA
F2A
TCRα
FLAG
S1 S2
L1
L2
S1 (Spacer 1): SRGGS(GGGS)4GAS
S2 (Spacer 2): GAGGS(GGGS)4G
L1 (Linker 1): EF
L2 (Linker 2): SG
B
CD3 (MFI - bkg) (WT = 1)
****
WT
βA291
C
CD3 (MFI - bkg) (WT = 1)
D
pErk (MFI - bkg) (WT = 1)
E
p < 0.0001
pErk (MFI norm. to max)
WT
βA291
(6V-A2)4 (nM)
kp
****
F
pζ (MFI - bkg)
*
G
ζKO
WT
βA291
UCHT1
IB:
pζ
ζ
pζ/ζ (WT = 1)
**
H
pζ/ε (MFI)
**
I
Normalized To Mode
WT
WT A770041
ζKO
pζ
J
β-HA (MFI - bkg) (WT = 1)
K
CD3 (MFI - bkg) (WT untreated = 1)
****
****
***
A770041

Fig S1

Figure S1. Gain-of-function mutations in the βTMR, related to Figure 1

A Scheme of the single polypeptide self-splicing 1G4 αβ expressed in J76 and $CD8^-$ J76 cells. β chain (blue), α chain (cyan), HA-tag (orange), FLAG-tag (magenta), F2A sequence (grey). The entire aa sequence can be found in **Table S1**. Spacer and short linker aa sequences are indicated below the scheme. **B** CD3 surface expression of $CD8^-$ J76 1G4-WT or 1G4-βA291. Cells were labelled or not with CellTrace violet, mixed 1:1 and analysed for CD3 surface expression and total TCRβ-HA by FACS. Gates for equal HA-tag expression level were applied and the CD3 MFI of 1G4-WT and 1G4-βA291 was extracted from the TCRβ-HA^{low} gate, see STAR Methods. Mean ± SEM of background subtracted CD3 MFI from the indicated gate normalised to the corresponding WT in the same gate, n = 3 experiments in duplicates, unpaired *t*-test $p < 0.0001$. **C** Similar CD3 surface expression of $CD8^-$ J76 1G4-WT and 1G4-βA291. Cells were induced with different doses of doxycycline, labelled or not with CellTrace violet, mixed 1:1 and analysed for CD3 surface expression by FACS, see STAR Methods. Mean ± SD of $CD3^+$ MFI normalised to WT, n = 3 experiments in triplicates, unpaired *t*-test (ns). **D** PMA/Iono induced pErk response of $CD8^-$ J76 1G4-WT or 1G4-βA291. Cells were labelled or not with CellTrace violet, mixed 1:1, treated with PMA/Iono (5') and analysed for pErk by FACS. Mean ± SD of pErk MFI normalised to WT, n = 3 experiments in triplicates, unpaired *t*-test (ns). **E** pErk response of $CD8^-$ J76 1G4-WT or 1G4-βA291 stimulated with $(6V\text{-}A2)_4$. Cells were induced with different doses of doxycycline, labelled or not with CellTrace violet, mixed 1:1 and stimulated for 3 min with different doses (0.78 – 600 nM) of PE-conjugated $(6V\text{-}A2)_4$ and analysed for pErk and $(6V\text{-}A2)_4$ binding by FACS. **Left**, $(6V\text{-}A2)_4$ nM vs. pErk MFI normalised to max (showed in Fig. **1C**) fitted to a minimal model of kinetic proofreading, n = 3 experiments in triplicates, $R^2 = 0.84$ (WT), 0.90 (βA291), F-test $p < 0.0001$. **Right**, mean ± SD of proofreading rate (k_p), n = 3 experiments in triplicates, F-test $p < 0.0001$. **F** Paired max. pζ values related to dose response reported in Fig. **1E**. J76 1G4-WT or 1G4-βA291 were labelled or not with CellTrace violet, mixed 1:1, stimulated for 1 min with increasing doses (3.125 - 200 nM) of PE-conjugated $(6V\text{-}A2)_4$ and analysed for pζ and $(6V\text{-}A2)_4$ binding by FACS. Background subtracted MFI of $(6V\text{-}A2)_4$ was plotted vs. pζ MFI and fitted by non-linear regression, n = 4, ratio paired *t*-test $p = 0.023$. **G** pζ response of J76 1G4-WT or 1G4-βA291 stimulated or not with UCHT1 Ab. **Left**, immunoblot (IB) representative of 4 experiments. **Right**, mean ± SD of pζ/ζ, n = 4, unpaired *t*-test $p = 0.0013$. **H** Paired mean values of the basal pζ in J76 1G4-WT or 1G4-βA291 reported in Fig. **1F**. Resting cells were analysed for pζ or CD3 surface expression by FACS. pζ MFI was normalised to surface CD3 MFI, n = 8, paired *t*-test $p = 0.0037$. **I** Representative FACS histograms of pζ staining related to Fig **1F**. J76 1G4-WT cells were treated or not with A770041 (5 µM) for 15 min at 37 ºC and analysed for pζ by FACS. J76-1G4WT-ζKO served as a negative control. **J** β-HA total expression of $CD8^-$ J76 1G4-WT or 1G4-βA291. Cells were labelled or not with CellTrace violet, mixed 1:1 and analysed for CD3 surface expression and total TCRβ-HA by FACS in a single HA bin, see STAR Methods. Mean ± SEM of background subtracted HA MFI in HA^{low} gate, n = 3, unpaired *t*-test (ns). **K** TCR-CD3 surface expression in J76 1G4-WT or 1G4-βA291 treated or not with A770041, n = 5, unpaired *t*-test $p < 0.0001$ (βA291 vs. WT), $p < 0.0001$ (WT ± A770041), $p = 0.0003$ (βA291 ± A770041).

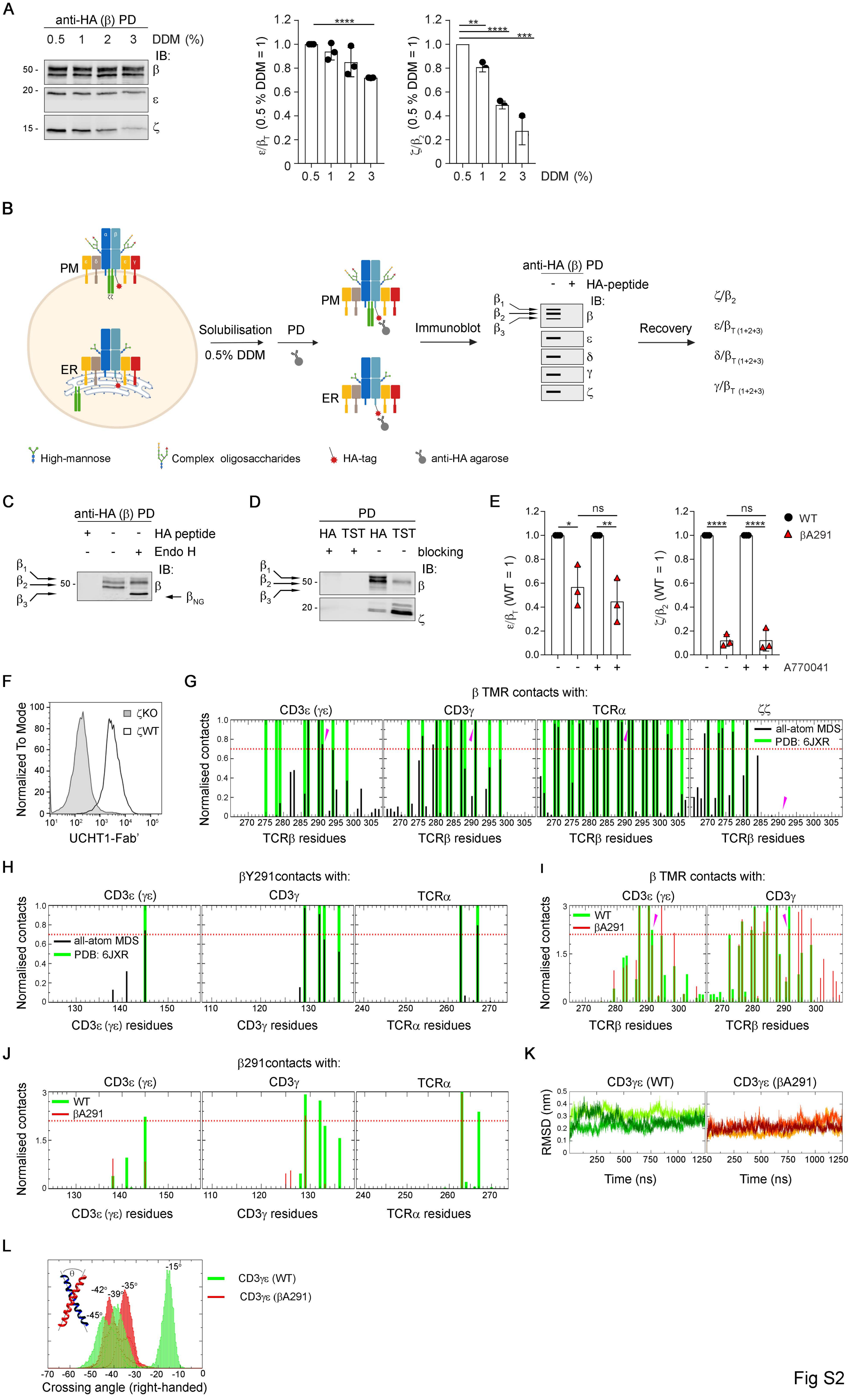
A
anti-HA (β) PD
DDM (%)
IB:
B
Solubilisation
0.5% DDM
PD
Immunoblot
Recovery
High-mannose
Complex oligosaccharides
HA-tag
anti-HA agarose
C
D
E
WT
βA291
A770041
F
Normalized To Mode
UCHT1-Fab'
ζKO
ζWT
G
β TMR contacts with:
TCRβ residues
all-atom MDS
PDB: 6JXR
H
βY291contacts with:
I
J
β291contacts with:
K
Time (ns)
L
Crossing angle (right-handed)
CD3γε (WT)
CD3γε (βA291)

Fig S2

Figure S2. βY291 contribution to TCR-CD3 quaternary structure cohesion, related to Figure 2

A Sensitivity of TCR-CD3 quaternary structure cohesion to DDM concentrations. $CD8^-$ J76 1G4-WT were solubilised with increasing concentrations of DDM and analysed by β-HA PD and IB for β, ε and ζ (**left**). Mean ± SD of ε/β_T (**middle**) and ζ/β_2 (**right**), n = 3, unpaired *t*-test (ε/β_T): $p < 0.0001$; (ζ/β_2): 0.5 vs. 1 % $p < 0.01$, 0.5 vs. 2 % $p < 0.0001$, 0.5 vs. 3 % $p < 0.001$. Note that at ≤ 0.5 % DDM there is no detectable change in cohesion of CD3 subunits with TCRαβ, further supporting that in TCR-CD3 extracted at 0.5 % DDM the subunits' stoichiometry in the octamer remains intact (Swamy et al., 2008). At ≥ 1 % DDM there is progressive loss of ζ followed by γε and δε. **B** Graphic scheme describing the DSA. The entire pool of the TCRαβ is captured by anti-HA (β-HA) PD which includes partial αβγεδε complex assembled in the endoplasmic reticulum (ER) (Alcover et al., 2018), αβγεδεζζ complex resident in the *trans*-Golgi (Alcover et al., 2018) or recycling at steady state in the ER (Alcover et al., 2018) and the largest fraction of αβγεδεζζ present at the plasma membrane (PM). The IB scheme on the right shows expected band patterns for β, ε, δ, γ and ζ derived from the ER and PM. Arrows indicate the different isoforms of TCRβ (β_1, β_2, β_3). To evaluate ζ recovery, ζ/β_2 ratio was calculated and the value for ζ/β_2 ratio from WT was set equal to one. To evaluate ε, δ, γ recovery, ε/β_T, δ/β_T and γ/β_T ratios were calculated and the ratios for WT were set equal to one. These represented the recovery of intact TCR-CD3 complex. Ratios < 1 indicate a lower recovery of ζ, ε, δ and γ hence a reduced cohesion of TCR-CD3 quaternary structure. See STAR Methods for a detailed description of the experimental procedure. **C** J76 wtc51 treated or not with endo H and analysed by β-HA PD and IB for β. Arrows indicate β isoforms, β_{NG} indicates non-glycosylated β isoform after removal of high-mannose carbohydrates by endo-H treatment. Data representative of 2 experiments. **D** J76-1G4-ζKO expressing inducible ζTST were solubilised and analysed by β-HA (lanes 1, 3) or ζTST (lanes 2, 4) PD and IB for β and ζ. Arrows indicate β isoforms. IB representative of 2 experiments. **E** $CD8^-$ J76 1G4-WT or 1G4-βA291 ± A770041 were solubilised and analysed by β-HA PD and IB for β, ε and ζ. Mean ± SD of ε/β_T (**left**) and ζ/β_2 (**right**), n = 3, unpaired *t*-test (ε/β_T): 1G4-WT vs. 1G4-βA291 $p < 0.05$, 1G4-WT+A770041 vs. 1G4-βA291+A770041 $p < 0.01$; (ζ/β_2): 1G4-WT vs. 1G4-βA291 $p < 0.0001$, 1G4-WT+A770041 vs. 1G4-βA291+A770041 $p < 0.0001$; ns = non-significant. **F** Representative FACS histograms of UCHT1-Fab' staining of J76-1G4WT-ζKO reconstituted with doxycycline inducible ζWT. Cells were induced or not for ζWT expression, labelled or not with CellTrace violet, mixed 1:1 and analysed for TCR-CD3 surface expression by FACS. **G** Normalised number of contacts of the WT β TMR with the rest of the TCR-CD3 TMRs in our all-atom molecular dynamics simulations (MDS) (related to Fig. **2D**). The contacts in the cryo-EM structure (PDB: 6JXR) are shown in green for comparison with the WT all-atom MDS (black). Magenta arrow indicates β291. Normalisation was done by dividing the number of contacts of each residue by the highest number of contacts. For all contacts analyses in Figs. **S2G** - **S2J**, a cut-off distance of 4 Å was used to define a contact and the red dotted line represents 70 % of the normalised contacts, a threshold used to measure the significance of contacts. **H** Normalised number of contacts of βY291 with the rest of the TCR-CD3 TMRs (related to Fig. **2D**). Comparison of protein-protein interactions between our WT all-atom MDS (black) and the cryo-EM structure (PDB: 6JXR) (green). Normalisation was done by dividing the number of contacts of each residue by the highest number of contacts. **I** Normalised number of contacts of βWT (green) and βA291 (red) with CD3γε TMR in our all-atom MDS. Magenta arrow indicates β291. Normalisation

was done by dividing the number of contacts of each residue by the number of simulation frames. **J** Normalised number of contacts of βY291 WT (green) and βA291 mutant (red) in our all-atom MDS. Normalisation was done by dividing the number of contacts of each residue by the number of simulation frames. **K** Root mean square deviations (RMSD) of the Cα atoms of CD3γε relative to their initial configuration during the WT and βA291 all-atom MDS. Each line represents RMSD obtained from one simulation ($n = 3$). **L** Comparison of crossing angle distribution of CD3γε TMR between WT and βA291, in 3 simulations. The most observed crossing angle values are labelled.

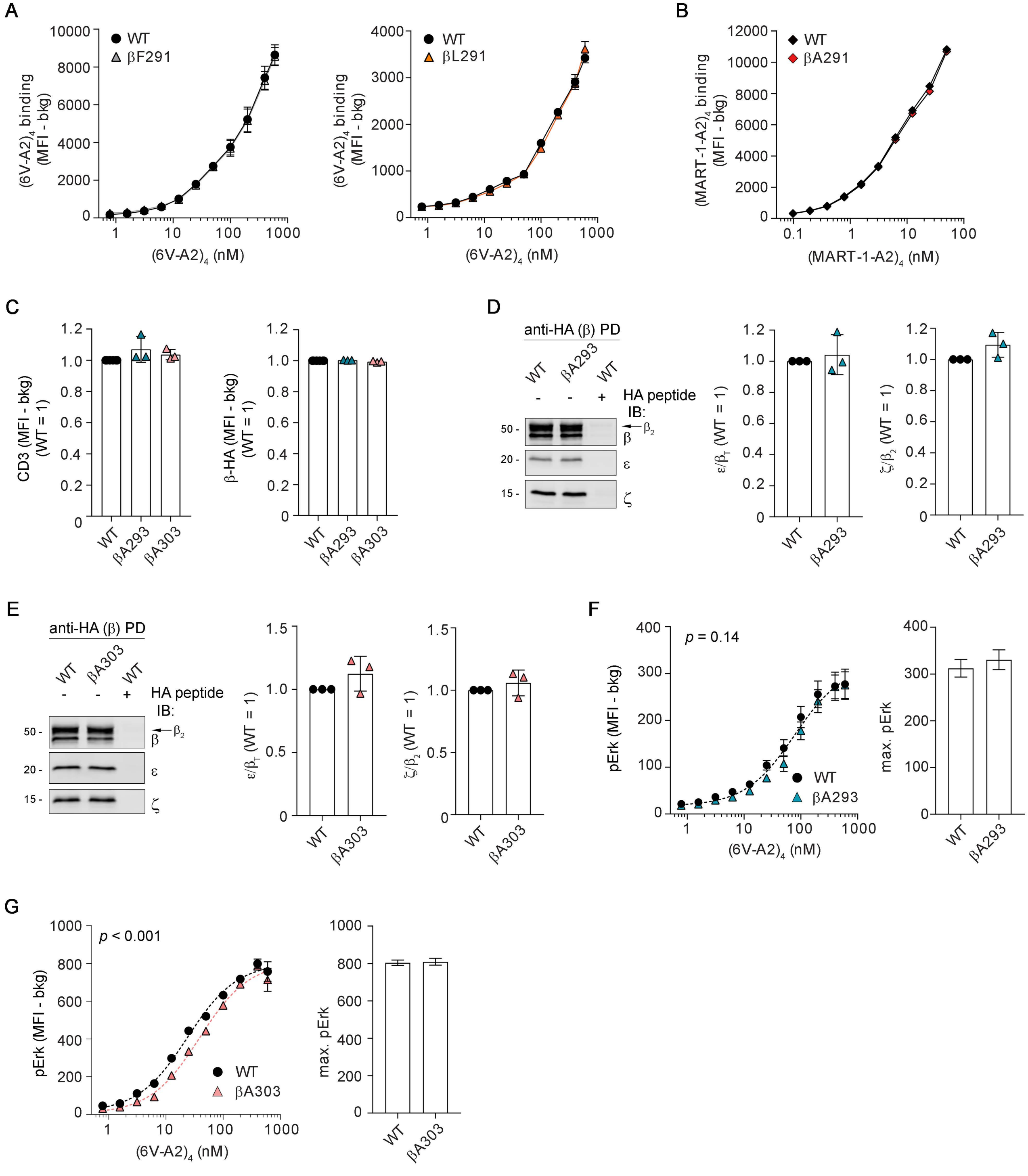

A
WT
βF291
(6V-A2)4 binding (MFI - bkg)
(6V-A2)4 (nM)
WT
βL291
B
WT
βA291
(MART-1-A2)4 binding (MFI - bkg)
(MART-1-A2)4 (nM)
C
CD3 (MFI - bkg) (WT = 1)
β-HA (MFI - bkg) (WT = 1)
WT
βA293
βA303
D
anti-HA (β) PD
HA peptide
IB:
β2
β
ε
ζ
ε/βT (WT = 1)
ζ/β2 (WT = 1)
E
F
p = 0.14
pErk (MFI - bkg)
max. pErk
G
p < 0.001

Fig S3

Figure S3. Loosening ζ association enhances signalling, related to Figure 3

A $(6V\text{-}A2)_4$ binding to $CD8^-$ J76 1G4-WT or 1G4-βF291 (**left**) or 1G4-βL291 (**right**) related to Figs. **3C** and **3D**. Cells were induced with different doses of doxycycline, labelled or not with CellTrace violet, mixed 1:1 and stimulated for 3 min with different doses (0.78 – 600 nM) of PE-conjugated $(6V\text{-}A2)_4$ and analysed by FACS. Plots show mean ± SD of 3 experiments measured in triplicates. **B** $(MART\text{-}1\text{-}A2)_4$ binding to $CD8^-$ J76 2H5-WT or 2H5-βA291 related to Fig. **3G**. Cells were induced with different doses of doxycycline, labelled or not with CellTrace violet, mixed 1:1 and stimulated for 3 min with different doses (0.05 – 50 nM) of PE-conjugated $(MART\text{-}1\text{-}A2)_4$ and analysed by FACS. Plot shows mean ± SD of 3 experiments measured in triplicates. **C** TCR-CD3 surface expression in $CD8^-$ J76 of 1G4-WT, 1G4-βA293 or 1G4-βA303. Cells were labelled or not with CellTrace violet, mixed 1:1 and analysed for CD3 surface expression and total TCRβ-HA by FACS. Gates for equal HA-tag expression level were applied and the CD3 and β-HA MFI were extracted from the TCRβ-HA^{low} gate, see STAR Methods. **Left**, mean ± SEM of CD3 MFI in HA^{low} gate, 3 experiments measured in duplicates, *t*-test (ns). **Right**, mean ± SEM of β-HA MFI in HA^{low} gate, 3 experiments measured in duplicates, *t*-test (ns). **D** $CD8^-$ J76 1G4-WT or 1G4-βA293 were solubilised and analysed by β-HA PD and IB for β, ε and ζ. **Left**, IB: 1 of 3 experiments. The arrow indicates β_2 isoform. **Middle**, mean ± SD of ε/β_T, n = 3, *t*-test (ns). **Right**, mean ± SD of ζ/β_2, n = 3, unpaired *t*-test (ns). **E** $CD8^-$ J76 1G4-WT or 1G4-βA303 were solubilised and analysed by β-HA PD and IB for β, ε and ζ. **Left**, IB: 1 of 3 experiments. The arrow indicates β_2 isoform. **Middle**, mean ± SD of ε/β_T, n = 3, *t*-test (ns). **Right**, mean ± SD of ζ/β_2, n = 3, unpaired *t*-test (ns). **F** pErk response of $CD8^-$ J76 1G4-WT or 1G4-βA293. Cells were induced with different doses of doxycycline, labelled or not with CellTrace violet, mixed 1:1 and stimulated for 3 min with different doses (0.78 – 600 nM) of PE-conjugated $(6V\text{-}A2)_4$ and analysed by FACS. **Left**, non-linear regression fit of $(6V\text{-}A2)_4$ nM vs. pErk MFI, n = 3, $R^2 = 0.52$ (WT), 0.60 (βA293). **Right**, mean ± SD of max. pErk, n = 3, F-test (ns). **G** pErk response of $CD8^-$ J76 1G4-WT or 1G4-βA303. Cells were induced with different doses of doxycycline, labelled or not with CellTrace violet, mixed 1:1 and stimulated for 3 min with different doses (0.78 – 600 nM) of PE-conjugated $(6V\text{-}A2)_4$ and analysed by FACS. **Left**, non-linear regression fit of $(6V\text{-}A2)_4$ nM vs. pErk MFI, n = 3, $R^2 = 0.96$ (WT), 0.95 (βA293). **Right**, mean ± SD of max. pErk, n = 3, F-test (ns).

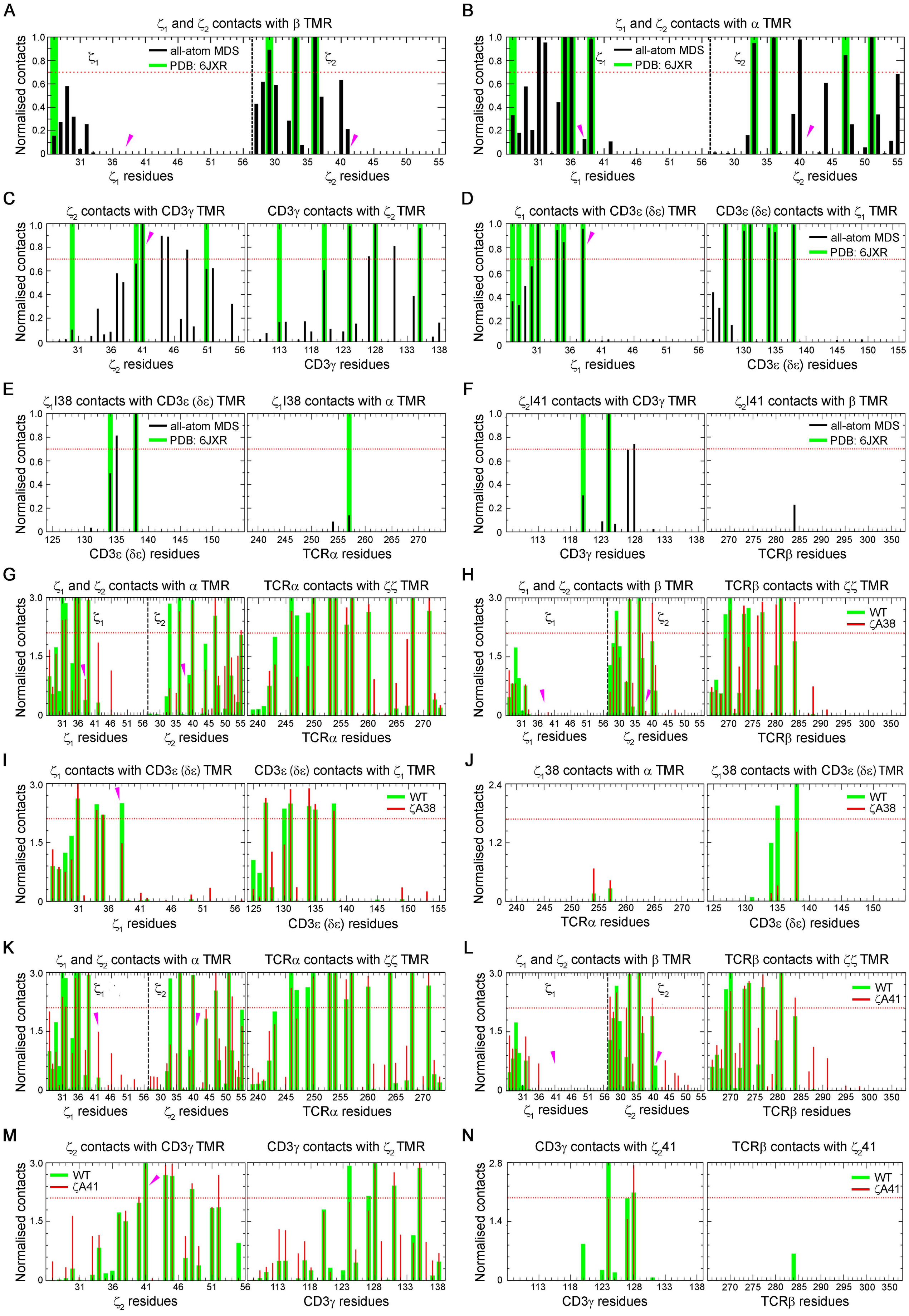

A
ζ1 and ζ2 contacts with β TMR
B
ζ1 and ζ2 contacts with α TMR
C
ζ2 contacts with CD3γ TMR
CD3γ contacts with ζ2 TMR
D
ζ1 contacts with CD3ε (δε) TMR
CD3ε (δε) contacts with ζ1 TMR
E
ζ1I38 contacts with CD3ε (δε) TMR
ζ1I38 contacts with α TMR
F
ζ2I41 contacts with CD3γ TMR
ζ2I41 contacts with β TMR
G
ζ1 and ζ2 contacts with α TMR
TCRα contacts with ζζ TMR
H
ζ1 and ζ2 contacts with β TMR
TCRβ contacts with ζζ TMR
I
ζ1 contacts with CD3ε (δε) TMR
CD3ε (δε) contacts with ζ1 TMR
J
ζ138 contacts with α TMR
ζ138 contacts with CD3ε (δε) TMR
K
ζ1 and ζ2 contacts with α TMR
TCRα contacts with ζζ TMR
L
ζ1 and ζ2 contacts with β TMR
TCRβ contacts with ζζ TMR
M
ζ2 contacts with CD3γ TMR
CD3γ contacts with ζ2 TMR
N
CD3γ contacts with ζ241
TCRβ contacts with ζ241
Normalised contacts
all-atom MDS
PDB: 6JXR
WT
ζA38
ζA41
ζ1 residues
ζ2 residues
CD3γ residues
CD3ε (δε) residues
TCRα residues
TCRβ residues

Fig S4

Figure S4. Loosening ζ association enhances signalling, related to Figure 4

A Normalised number of contacts of ζ_1 and ζ_2 with β TMR, in our WT all-atom MDS (black) and in the cryo-EM structure (PDB: 6JXR) (green), related to Fig. **4A**. Magenta arrow indicates ζ_1I38 (**left**) or ζ_2I41 (**right**). In Figs. **S4A** - **S4F**, when comparing the contacts in the cryo-EM structure to our WT all-atom MDS, normalisation was done by dividing the number of contacts of each residue by the highest number of contacts. For all contacts analyses in Figs. **S4A** - **S4N**, a cut-off distance of 4 Å was used to define a contact and the red dotted line represents 70 % of the normalised contacts, a threshold used to measure the significance of contacts. **B** Normalised number of contacts of ζ_1 and ζ_2 with α TMR, in our WT all-atom MDS (black) and in the cryo-EM structure (PDB: 6JXR) (green), related to Fig. **4B**. Magenta arrow indicates ζ_1I38 (**left**) or ζ_2I41 (**right**). **C** Normalised number of contacts of ζ_2 TMR with CD3γ TMR (**left**) and of CD3γ with ζ_2 TMR (**right**), related to Fig. **4C**. Comparison between protein-protein interactions resulted from our WT all-atom MDS (black) and the cryo-EM structure (PDB: 6JXR) (green). Magenta arrow indicates ζ_2I41. **D** Normalised number of contacts of ζ_1 with CD3ε (δε) TMR (**left**) and of CD3ε (δε) with ζ_1 TMR (**right**), related to Fig. **4D**. Comparison between protein-protein interactions resulted from our WT all-atom MDS (black) and the cryo-EM structure (PDB: 6JXR) (green). Magenta arrow indicates ζ_1I38. **E** Normalised number of interactions of ζ_1I38 with the rest of TCR-CD3 TMRs. Comparison between protein-protein interactions resulted from our WT all-atom MDS (black) and the cryo-EM structure (PDB: 6JXR) (green). **F** Normalised number of interactions of ζ_2I41 with the rest of TCR-CD3 TMRs. Comparison between protein-protein interactions resulted from our WT all-atom MDS (black) and the cryo-EM structure (PDB: 6JXR) (green). **G** Normalised number of contacts of ζ_1 and ζ_2 with α TMR (**left**) and of α TMR with ζζ TMR (**right**) in the WT (green) and ζA38 (red) all-atom MDS. Magenta arrow indicates ζ38 showing no significant contacts with α TMR in both WT and ζA38 simulations. However, one contact of ζ_2 (ζ_2Y33) with α TMR and two contacts of α TMR (αL247, αV249) with ζζ TMRs are reduced in the simulations carrying ζA38 substitution. For Figs. **S4G** - **S4N**, when comparing the contacts in the WT simulations to the contacts of the mutants, normalisation was done by dividing the number of contacts of each residue by the number of simulation frames. **H** Normalised number of contacts of ζ_1 and ζ_2 with β TMR (**left**) and of β TMR with ζζ TMR (**right**) in the WT (green) and ζA38 mutant (red) all-atom MDS. Magenta arrow indicates ζ38 showing no significant contacts with β TMR in both WT and ζA38 simulations. However, an increase in the interactions between ζ_2 (ζ_2F40) and β (βS276, βL280, βL284) were observed in the simulations carrying ζA38 substitution. This is likely to be the consequence of the increased ζζ loosening that allows ζ_2 to wobble and to come in contact with β TMR. **I** Normalised number of contacts of ζ_1 with CD3ε (δε) TMR (**left**) and of CD3ε (δε) with ζ_1 TMR (**right**) in the WT (green) and ζA38 mutant (red) all-atom MDS. Magenta arrow indicates $\zeta_1$38 showing that this residue reduced its interaction with CD3ε (δε) TMR when mutated to alanine (ζA38, in red), with a net effect of increasing flexibility of both ζ_1 and ζ_2 subunits ($\zeta_1 > \zeta_2$) relative to TCRαβ (see also Fig. **4F**). **J** Normalised number of contacts of α TMR (**left**) and of CD3ε (δε) TMR (**right**) with $\zeta_1$38 in the WT (green) and ζA38 mutant (red) all-atom MDS. A small number of contacts between $\zeta_1$38 and α TMR were observed in both WT and ζA38 simulations while all three residues of CD3ε (δε) TMR (εV134, εI135 and εI138) that interacted in the WT simulations reduced their contacts with ζ_1A38 (red). Normalisation is performed such that the number of contacts of TCRα TMR is compared to that of CD3ε (δε) residues. **K** Normalised number of contacts of ζ_1 and ζ_2 with α TMR (**left**) and of α TMR with ζζ TMR (**right**)

in the WT (green) and ζA41 mutant (red) all-atom MDS. Magenta arrow indicates ζ41 showing no significant contacts with α TMR in both WT and ζA41 simulations. However, one contact of ζ_2 (ζ_2Y33) with α TMR and two contacts of α TMR (αL247, αV249) with ζζ TMRs were reduced in the simulations carrying ζA41 substitution. However, one contact of ζ_2 (ζ_2R52) with α TMR increased on ζA41 substitution. **L** Normalised number of contacts of ζ_1 and ζ_2 with β TMR (**left**) and of β TMR with ζζ TMR (**right**) in the WT (green) and ζA41 mutant (red) all-atom MDS. Magenta arrow indicates ζ41 showing no significant contacts of $\zeta_1$41 and $\zeta_2$41 with β TMR in both WT and ζA41 simulations. However, two contacts of ζ_2 with β TMR (ζ_2L27 and ζ_2F40) and one of β TMR with ζζ TMRs (βL284) were increased in the simulations carrying ζA41 substitution. However, one residue of the β TMR (βS269) reduced contact with ζζ TMR during the ζA41 simulations. **M** Normalised number of contacts of ζ_2 with CD3γ TMR (**left**) and of CD3γ with ζ_2 TMR (**right**) in the WT (green) and ζA41 mutant (red) all-atom MDS. Magenta arrow indicates ζ41 showing that the mutated residue ζ_2A41 still maintained contact with CD3γ during the simulations compared to the WT. However, γV124, which strongly interacted with ζ_2I41 in the WT, reduced its interaction with ζ_2A41. One contact of ζ_2 (ζ_2R52) with CD3γ TMR increased in the simulations carrying the ζA41 substitution. **N** Normalised number of contacts of CD3γ TMR (**left**) and of β TMR (**right**) with $\zeta_2$41 in the WT (green) and ζA41 mutant (red) all-atom MDS. Two contacts of CD3γ (γV124 and γF127) with $\zeta_2$41 were reduced and one contact (γV128) was increased during ζA41 simulation. No significant contacts of β TMR with $\zeta_2$41 were observed in both WT and ζA41 simulations.

A

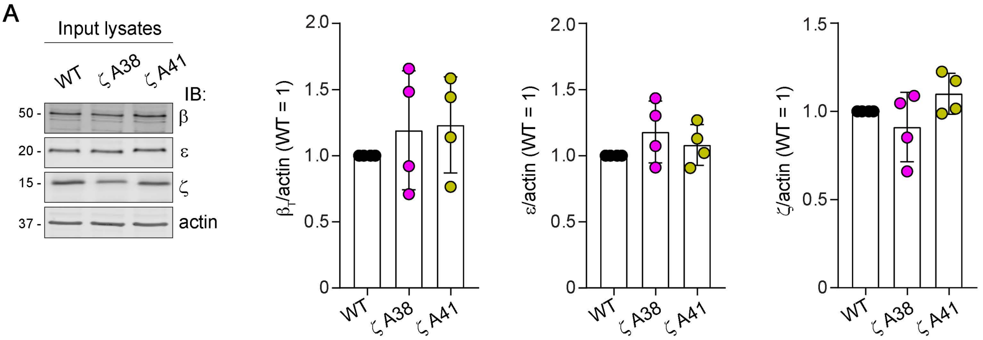

Input lysates
WT
ζ A38
ζ A41
IB:
50 -
β
20 -
ε
15 -
ζ
37 -
actin
βT/actin (WT = 1)
ε/actin (WT = 1)
ζ/actin (WT = 1)

B

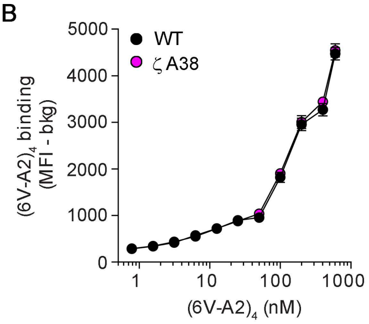

WT
ζ A38
(6V-A2)4 binding (MFI - bkg)
(6V-A2)4 (nM)

C

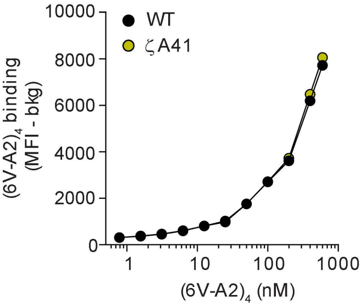

WT
ζ A41
(6V-A2)4 binding (MFI - bkg)
(6V-A2)4 (nM)

Fig S5

Figure S5. Loosening ζ association enhances signalling, related to Figure 5

A J76-1G4WT-ζKO expressing 1G4 ζWT or ζA38 or ζA41 were lysed with 0.5 % DDM and analysed by IB for β, ε, ζ and actin. **Left**, IB of input lysates of the experiment shown in Fig. **5B** (1 of 4 experiments). **Right**, mean ± SD of β_T/actin, ε/actin and ζ/actin normalized to WT, n = 4, unpaired *t*-test (ns). **B** $(6V\text{-}A2)_4$ binding to J76-1G4WT-ζKO expressing ζWT or ζA38 related to Fig. **5C**. Cells were induced with different doses of doxycycline, labelled or not with CellTrace violet, mixed 1:1 and stimulated for 3 min with different doses (0.78 – 600 nM) of PE-conjugated $(6V\text{-}A2)_4$ and analysed by FACS. Plot shows mean ± SD of 3 experiments measured in triplicates. **C** $(6V\text{-}A2)_4$ binding to J76-1G4WT-ζKO expressing ζWT or ζA41 related to Fig. **5D**. Cells were induced with different doses of doxycycline, labelled or not with CellTrace violet, mixed 1:1 and stimulated for 3 min with different doses (0.78 – 600 nM) of PE-conjugated $(6V\text{-}A2)_4$ and analysed by FACS. Plot shows mean ± SD of 3 experiments measured in triplicates.

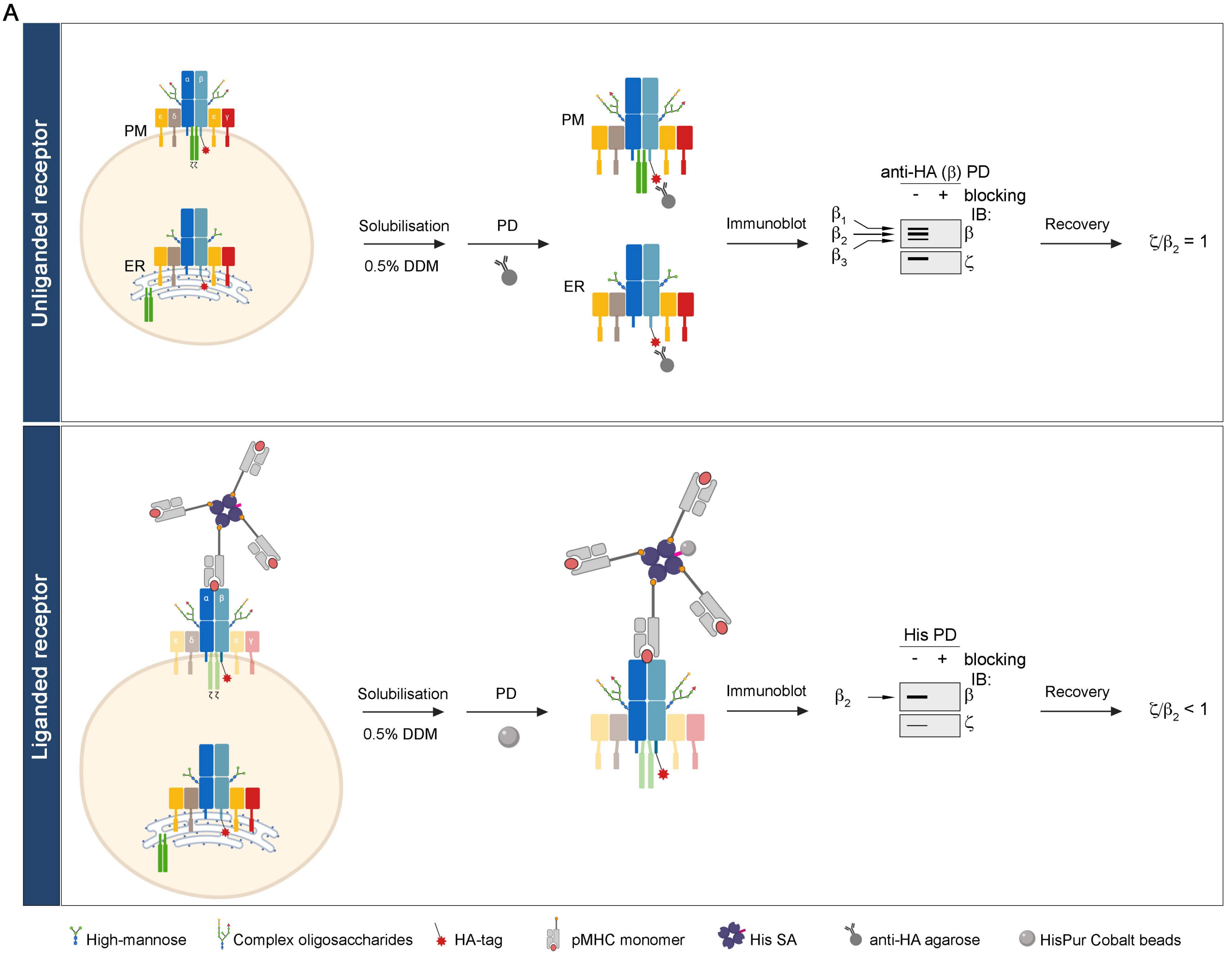

B

	K_d(nM)	CDR1α	CDR2α	CDR3α	CDR1β	CDR2β	CDR3β
1G4	1300	DRGSQS	IQSSQRE	CAVRPTSGGSYIPTFG	MNHEY	SVGAGI	CASSYVGNTGELFFG
QM-α	140	DRGSQS	IQS**W**QRE	CAVRPTSGGSYIPTFG	MNHEY	SV**AE**GI	CASSYVGNTG**LA**FFG
wtc51	15	DRGSQS	IQSSQRE	CAVRPTSGGSYIPTFG	MNHEY	SV**AIQT**	CASSYVGNTGELFFG

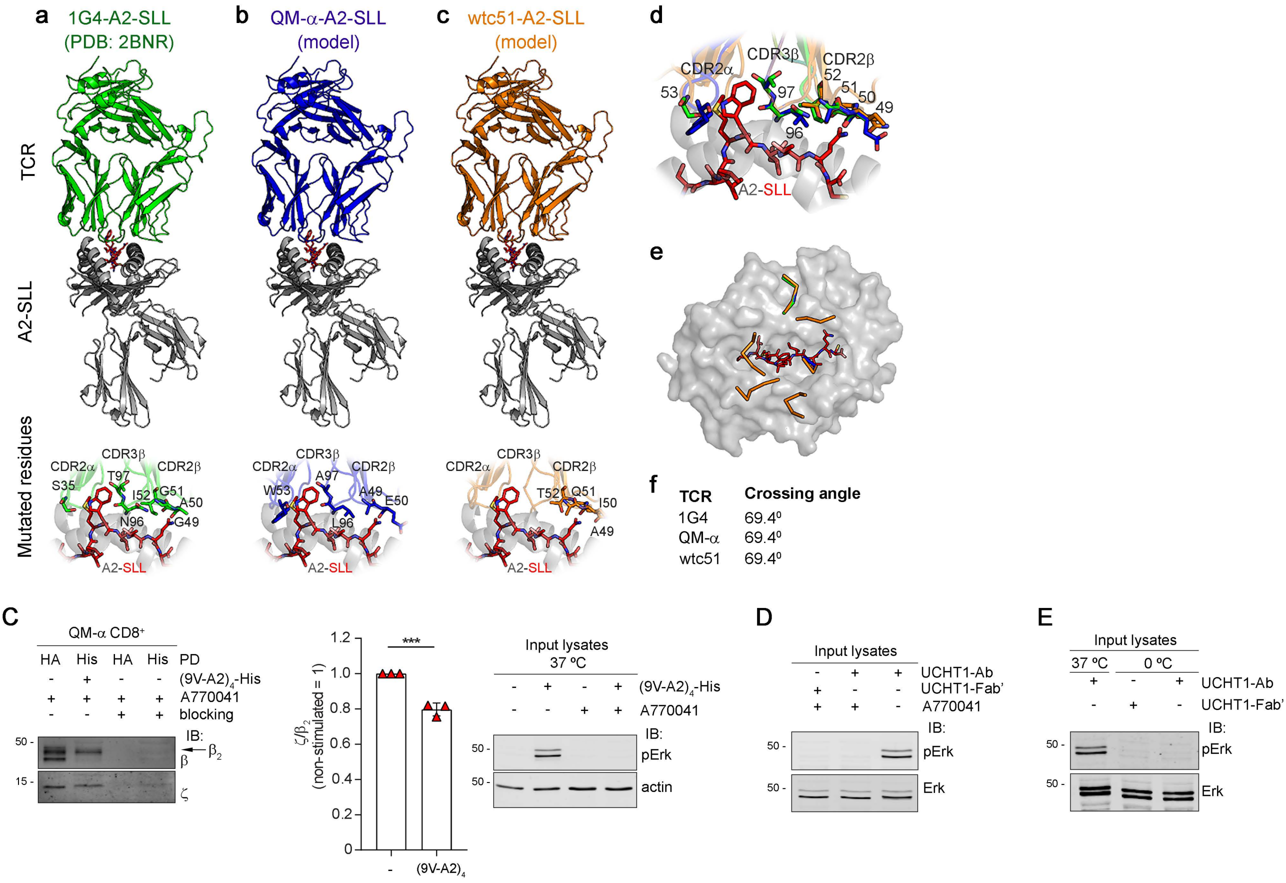

Fig S6

Figure S6. pMHC tetramer binding loosens αβ association with ζ, related to Figure 6

A Graphical scheme describing the experimental procedure used to compare the cohesion of unliganded receptor (**top**) and $(pMHC)_4$ ligated TCR-CD3 (liganded receptor, **bottom**). The unliganded receptor was captured by anti-HA (β-HA) PD described in Fig. **S2B**. To pull down the liganded receptor, cells were stimulated with tetramerised His-tagged streptavidin (His SA) and ligand excess was removed. Cells were solubilised with 0.5 % DDM and post-nuclear lysate was incubated with HisPur Cobalt beads to pull down $pMHC_4$-engaged TCR-CD3. The IB schemes on the right show expected bands pattern for β and ζ. Arrows indicate the different isoforms of TCRβ (β_1, β_2, β_3). To evaluate ζ recovery, ζ/β_2 ratio was calculated and the value for ζ/β_2 ratio from non-stimulated samples was set equal to one. This value represented the recovery of intact TCR-CD3 complex and was compared to ζ/β_2 ratio from $(pMHC)_4$ stimulated samples. A ratio < 1 indicates a lower recovery of ζ revealing a reduced cohesion of TCR-CD3 quaternary structure. See STAR Methods for a detailed description of the experimental procedure. **B** Structure and modelling of 1G4-WT and affinity enhanced 1G4 mutants (QM-α and wtc51) used in this study. **Top**, table shows binding affinities of the 1G4 and affinity enhanced TCR mutants (QM-α and wtc51) with sequence alignment highlighting the CDR loops with mutated residues (bold and underlined). **a**, structural overview of the 1G4 TCR (green)-A2 (grey)-SLL (red sticks) tri-molecular complex structure (PDB: 2BNR). Positions of the TCR residues (green sticks) in relation to A2-SLL that are mutated in the affinity enhanced TCRs are shown below. **b**, structural overview of the QM-α TCR (blue)-A2-SLL tri-molecular complex structure (mutations modelled using PDB: 2BNR). Positions of the TCR residues (blue sticks) in relation to A2-SLL that are mutated in the affinity enhanced TCR are shown below. **c**, structural overview of the wtc51 TCR (orange)-A2-SLL tri-molecular complex structure (mutations modelled using PDB: 2BNR). Positions of the TCR residues (orange sticks) in relation to A2-SLL that are mutated in the affinity enhanced TCRs are shown below. **d**, overlay of the mutated residues in the CDR loops comparing 1G4 (green sticks), QM-α (blue sticks) and wtc51 (orange sticks) TCRs. **e**, overlay of the positions of the CDR loops comparing 1G4 (green ribbon), QM-α (blue ribbon) and wtc51 (orange ribbon). **f**, analysis of the TCR crossing angles for each TCR. **C** J76 QM-α treated with A770041 and stimulated or not with $(9V\text{-}A2)_4$-His. **Left**, β-HA (lanes 1, 3) or His (lanes 2, 4) PD and IB for β and ζ (1 of 3 experiments). The arrow indicates β_2 isoform. **Middle**, mean ± SD of ζ/β_2, n = 3, unpaired *t*-test $p < 0.001$. **Right**, pErk IB: 1 of 3 experiments. **D** J76 1G4 ± A770041 were incubated with or w/o UCHT1-Fab' or UCHT1-Ab. pErk IB of the experiment shown in Fig. **6G** (1 of 3 experiments). **E** J76 1G4 were cooled on ice for 20 min and incubated for 5 min on ice with UCHT1-Fab' or UCHT1-Ab. pErk IB of the experiment shown in Fig. **6H** (1 of 3 experiments).

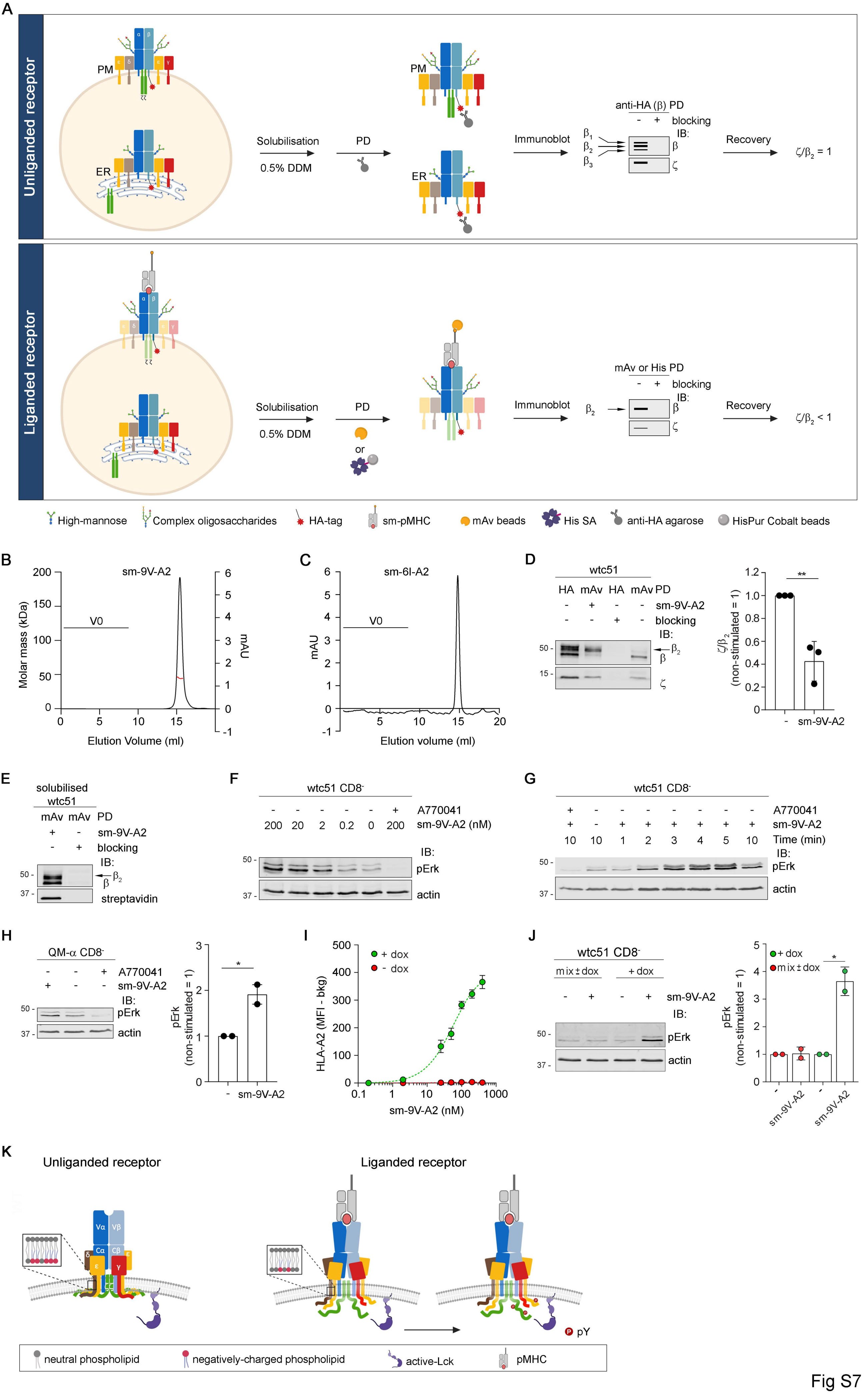
A
Unliganded receptor
PM
ER
Solubilisation
0.5% DDM
PD
Immunoblot
anti-HA (β) PD
blocking
IB:
Recovery
ζ/β2 = 1
Liganded receptor
mAv or His PD
or
ζ/β2 < 1
High-mannose
Complex oligosaccharides
HA-tag
sm-pMHC
mAv beads
His SA
anti-HA agarose
HisPur Cobalt beads
B
sm-9V-A2
Molar mass (kDa)
mAU
Elution Volume (ml)
V0
C
sm-6I-A2
Elution volume (ml)
D
wtc51
HA mAv HA mAv PD
sm-9V-A2
blocking
(non-stimulated = 1)
E
solubilised wtc51
streptavidin
F
wtc51 CD8-
A770041
sm-9V-A2 (nM)
pErk
actin
G
Time (min)
H
QM-α CD8-
pErk (non-stimulated = 1)
I
+ dox
- dox
HLA-A2 (MFI - bkg)
sm-9V-A2 (nM)
J
mix ± dox
+ dox
K
Unliganded receptor
Liganded receptor
neutral phospholipid
negatively-charged phospholipid
active-Lck
pMHC
pY

Fig S7

Figure S7. Monovalent pMHC in solution triggers TCR-CD3 untying and intracellular signalling, related to Figure 7

A Graphical scheme describing the experimental procedure used to compare the cohesion of unliganded receptor (**top**) and soluble, monovalent, mono-dispersed (sm)-pMHC ligated TCR-CD3 (liganded receptor, **bottom**). The unliganded receptor was captured by anti-HA (β-HA) PD described in Fig. **S2B**. To pull down the liganded receptor cells were stimulated with biotinylated sm-pMHC and ligand excess was removed. Cells were then solubilised with 0.5 % DDM and post-nuclear lysate was incubated with His-tagged streptavidin (His SA) followed by pull down with HisPur Cobalt beads or post-nuclear lysate was incubated with monomeric Avidin beads (mAv). The IB schemes on the right show expected bands pattern for β and ζ. Arrows on the left indicate the different isoforms of TCRβ (β_1, β_2, β_3). To evaluate ζ recovery, ζ/β_2 ratio was calculated and the value for ζ/β_2 ratio from the non-stimulated sample was set equal to one. This value represented the recovery of intact TCR-CD3 complex and was compared to ζ/β_2 ratio from sm-pMHC stimulated samples. A ratio < 1 indicates a lower recovery of ζ revealing a reduced cohesion of TCR-CD3 quaternary structure. See STAR Methods for a detailed description of the experimental procedure. **B** Size-exclusion chromatography-multi-angle-light scattering analysis of sm-9V-A2 showing a single, homogeneous peak of 44.6 kDa. The red line indicates the Molar mass (kDa). V0: void volume. **C** Gel-filtration chromatogram of sm-6I-A2 showing a single, homogeneous peak. V0: void volume. **D** J76 wtc51 stimulated or not with sm-9V-A2. **Left**, β-HA (lanes 1, 3) or monomeric Avidin (mAv) (lanes 2, 4) PD and IB for β and ζ (1 of 3 experiments). The arrow indicates β_2 isoform. **Right**, mean ± SD of ζ/β_2, n = 3, unpaired *t*-test $p < 0.01$. **E** $CD8^-$ J76 wtc51 were lysed, incubated or not with sm-9V-A2 and subjected to PD by anti-HA or monomeric Avidin (mAv). Monomeric Avidin (mAv) PD and IB for β and streptavidin IRDye 800CW. The arrow indicates β_2 isoform. **F** $CD8^-$ J76 wtc51 ± A770041 were stimulated or not with the indicated concentrations of sm-9V-A2 for 5 minutes. pErk IB representative of 2 experiments. **G** $CD8^-$ J76 wtc51 ± A770041 were stimulated or not with sm-9V-A2 for the indicated time points. pErk IB representative of 2 experiments. **H** $CD8^-$ J76 QM-α ± A770041 were stimulated or not with sm-9V-A2. **Left**, pErk IB (1 of 2 experiments). **Right**, mean ± SEM of pErk, n = 2, unpaired *t*-test $p = 0.05$. **I** Sm-9V-A2 binding to $CD8^-$ J76 wtc51. Cells were induced or not with doxycycline (dox), labelled with CellTrace violet or left untreated, mixed 1:1 and stimulated for 5 min with different doses (0.02 – 400 nM) of sm-9V-A2 at 37 °C. Cells were rapidly washed with FACS buffer, stained with anti-HLA-A2 and analysed by FACS. Non-linear regression fit of sm-9V-A2 (nM) vs. HLA-A2 (MFI), n = 3. **J** $CD8^-$ J76 wtc51 not doxycycline-induced (-dox) for TCR expression were reacted or not with 200 nM sm-9V-A2 for 5 minutes at 37 °C, washed and mixed with equal number of dox-induced $CD8^-$ J76 wtc51 (mix ± dox), see STAR Methods for a detailed protocol. Dox-induced (+ dox) $CD8^-$ J76 wtc51 stimulated or not with 200 nM sm-9V-A2 for 5 minutes at 37 °C served as control. **Left**, pErk IB (1 of 2 experiments). **Right**, mean ± SD of pErk of mixed dox-induced and not dox-induced ± sm-9V-A2 cells (mix ± dox), n = 2 experiments in duplicates, unpaired *t*-test: ns (mix ± dox), $p < 0.05$ (+ dox). **K** Graphical scheme describing the "TCR-CD3 allosteric relaxation" mechanism uncovered in this study. Briefly we suggest that in absence of force, co-receptor, clustering or PTPs exclusion, monovalent pMHC binding to TCR-CD3 allosterically regulates a cascade of conformational changes that relaxes the quaternary structure of TCR-CD3 TMRs. In the proposed model, conformational changes occurring at the pMHC binding site propagate to CαCβ ECDs at the site where they contact the CD3 subunits. These rearrangements are

transmitted to the CD3 TMRs, resulting in a reduced cohesion of TCR-CD3 TMRs, ITAMs exposure and their phosphorylation by active Lck. Moreover, we envisage the possibility that pMHC-induced reconfiguration of the octamer's TMRs leads to a local redistribution of negatively-charged lipids which could reduce hydrophobic and electrostatic forces that help holding CD3 tails within the plasma membrane.

Table S1. 1G4 amino acid sequence, related to Figure S1A and to the STAR methods section

1G4 WT αβ self-cleavable single polypeptide (TCRβ-spacer-HA-spacer-F2A-L1-TCRα-L2-Flag)
MSIGLLCCAALSLLWAGPVNAGVTQTPKFQVLKTGQSMTLQCAQDMNHEYMSWYRQDPGMGLRLIHYSVG<br>AGITDQGEVPNGYNVSRSTTEDFPLRLLSAAPSQTSVYFCASSYVGNTGELFFGEGSRLTVLEDLKNVFPPEVA<br>VFEPSEAEISHTQKATLVCLATGFYPDHVELSWWVNGKEVHSGVSTDPQPLKEQPALNDSRYCLSSRLRVSA<br>TFWQNPRNHFRCQVQFYGLSENDEWTQDRAKPVTQIVSAEAWGRADCGFTSESYQQGVLSATILYEILLGKA<br>TLYAVLVSALVLMAMVKRKDFSRGGSGGGSGGGSGGGSGGGSGASYPYDVPDYAGAGGSGGGSGGGSGG<br>GSGGGSGVKQTLNFDLLKLAGDVESNPGPEFMETLLGLLILWLQLQWVSSKQEVTQIPAALSVPEGENLVLN<br>CSFTDSAIYNLQWFRQDPGKGLTSLLLIQSSQREQTSGRLNASLDKSSGRSTLYIAASQPGDSATYLCAVRPTS<br>GGSYIPTFGRGTSLIVHPYIQNPDPAVYQLRDSKSSDKSVCLFTDFDSQTNVSQSKDSDVYITDKTVLDMRSM<br>DFKSNSAVAWSNKSDFACANAFNNSIIPEDTFFPSPESSCDVKLVEKSFETDTNLNFQNLSVIGFRILLLKVAGF<br>NLLMTLRLWSSSGDYKDDDDK

Table S2. gRNA and Primers, related to the STAR methods section

CRISPR gRNA	
CD3ζ	CAGGCACAGTTGCCGATTACAGG
Gateway cloning	
1G4 β-HA-F2A-α-FLAG FW	GGGGACAAGTTTGTACAAAAAAGCAGGCTTAATGAGCATCGGCCT CCTGTGCTGTGCAGCC
1G4 β-HA-F2A-α-FLAG REV	GGGGACCACTTTGTACAAGAAAGCTGGGTTTTATTTGTCG TCGTCGTCTTTGTAGTCTCC
CD3ζ-TST FW	ACAAGTTTGTACAAAAAAGCAGGCTTCACCATGAAGTGGAAGGCG CTTTTCAC
CD3ζ-TST REV	ACCACTTTGTACAAGAAAGCTGGGTTTTATTTTTCGAACTGCGGGT GGCTC
Amplification of 1G4 WT from cDNA	
TCRα FW	GGATCCATGGAGACCCTCTTGGGCCTGCTT
TCRα REV	TCTAGAGCTGGACCACAGCCGCAGCGT
TCRβ FW	GGATCCATGAGCATCGGCCTCCTGTGCTGT
TCRβ REV	GGATCCATGAGCATCGGCCTCCTGTGCTGT

Table S3. Protein sequences used in the all atom simulations, related to to the STAR methods section

Chain	Residue range	TMR sequences used
CD3-ζ_1	27-57	LDPKLCYLLDGILFIYGVILTALFLRVKFSR
CD3-ζ_2	27-55	LDPKLCYLLDGILFIYGVILTALFLRVKF
TCR-α	239-273	DTNLNFQNLSVIGFRILLLKVAGFNLLMTLRLWSS
TCR-β	266-307	TSESYQQGVLSATILYEILLGKATLYAVLVSALVLMAMVKRK
CD3-δ	98-129	ELDPATVAGIIVTDVIATLLLALGVFCFAGHE
CD3-ε (δε dimer)	125-155	MDVMSVATIVIVDICITGGLLLLVYYWSKNR
CD3-γ	109-138	ELNAATISGFLFAEIVSIFVLAVGVYFIAG
CD3-ε (γε dimer)	125-156	MDVMSVATIVIVDICITGGLLLLVYYWSKNRK

Table S4. Bilayer lipid concentrations used in the all atom simulations, related to the STAR methods section

Lipid concentration (%)	POPC	POPS	POPE	SM	CHOL	PIP_2	PIP_3
Inner leaflet	10	20	40	-	20	8	2
Outer leaflet	50	-	10	20	20	-	-
