# Supplementary material for: Allosteric activation of T cell antigen receptor signaling by quaternary structure relaxation
Source: Cell Rep. 2021 Jul 13;36(2):109375. doi: 10.1016/j.celrep.2021.109375 (PMC8293630; doi:10.1016/j.celrep.2021.109375)
Supplement: Document S2. Article plus supplemental information [file mmc2.pdf]

Cell Reports

Article

Allosteric activation of T cell antigen receptor signaling by quaternary structure relaxation

Graphical abstract

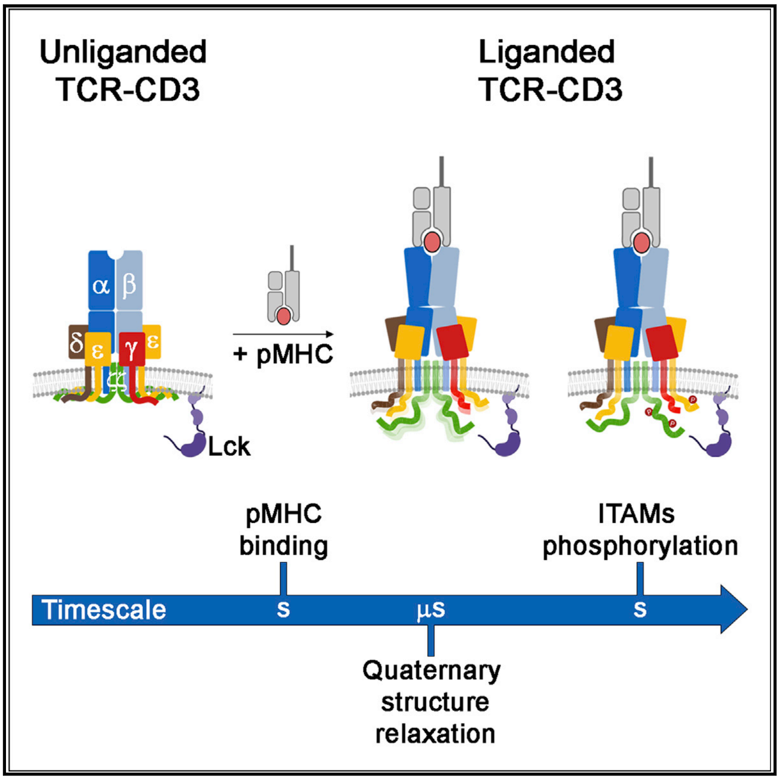

Highlights

- Mutations in TCRβ and CD3ζ TMRs that reduce their interaction augment signaling
- pMHC and anti-CD3 binding to TCR-CD3 induce similar quaternary structure relaxation
- Soluble monovalent pMHC alone signals and reduces TCRαβ cohesion with CD3ζ
- Allosteric changes in TCR-CD3 dynamics instigate T cell activation

Authors

Anna-Lisa Lanz, Giulia Masi, Nicla Porciello, ..., Mark S.P. Sansom, Antreas C. Kalli, Oreste Acuto

Correspondence

mark.sansom@bioch.ox.ac.uk (M.S.P.S.), a.kalli@leeds.ac.uk (A.C.K.), oreste.acuto@path.ox.ac.uk (O.A.)

In brief

T cell antigen receptor is central in adaptive immunity; however, the mechanism that couples ligand binding and intracellular signaling is still controversial. Here, Lanz et al. have taken an interdisciplinary approach demonstrating that binding of soluble monovalent pMHC to TCR-CD3 allosterically reduces TCRαβ cohesion with CD3ζ and initiates signal transduction.

Lanz et al., 2021, Cell Reports *36*, 109375
July 13, 2021 © 2021 The Authors.
https://doi.org/10.1016/j.celrep.2021.109375

CellPress

Cell Reports

CellPress OPEN ACCESS

Article

Allosteric activation of T cell antigen receptor signaling by quaternary structure relaxation

Anna-Lisa Lanz,[1,8,9] Giulia Masi,[1,8,10] Nicla Porciello,[1,8] André Cohnen,[1,8,11] Deborah Cipria,[1] Dheeraj Prakaash,[2] Štefan Bálint,[3] Roberto Raggiaschi,[1] Donatella Galgano,[1,12] David K. Cole,[4,5] Marco Lepore,[5] Omer Dushek,[6] Michael L. Dustin,[3] Mark S.P. Sansom,[7,*] Antreas C. Kalli,[2,7,*] and Oreste Acuto[1,13,*]

[1]T-cell signalling laboratory, Sir William Dunn School of Pathology, University of Oxford, Oxford OX1 3RE, UK
[2]Leeds Institute of Cardiovascular and Metabolic Medicine, University of Leeds, Leeds LS2 9JT, UK
[3]Kennedy Institute of Rheumatology, University of Oxford, Oxford OX3 7FY, UK
[4]Division Infection & Immunity, Cardiff University, Cardiff CF14 4XN, UK
[5]Immunocore Ltd., Abingdon OX14 4RY, UK
[6]Sir William Dunn School of Pathology, University of Oxford, Oxford OX1 3RE, UK
[7]Department of Biochemistry, University of Oxford, Oxford OX1 3QU, UK
[8]These authors contributed equally
[9]Present address: Department of Pediatrics, Ludwigs-Maximilians Universität, Munich, Germany
[10]Present address: Enara Bio, Oxford OX4 4GA, UK
[11]Present address: Bayer AG, Müllerstrasse 178, 13353 Berlin, Germany
[12]Present address: Department of Medicine, Karolinska Institutet, S-14186 Stockholm, Sweden
[13]Lead contact
*Correspondence: mark.sansom@bioch.ox.ac.uk (M.S.P.S.), a.kalli@leeds.ac.uk (A.C.K.), oreste.acuto@path.ox.ac.uk (O.A.)
https://doi.org/10.1016/j.celrep.2021.109375

SUMMARY

The mechanism of T cell antigen receptor (TCR-CD3) signaling remains elusive. Here, we identify mutations in the transmembrane region of TCRβ or CD3ζ that augment peptide T cell antigen receptor (pMHC)-induced signaling not explicable by enhanced ligand binding, lateral diffusion, clustering, or co-receptor function. Using a biochemical assay and molecular dynamics simulation, we demonstrate that the gain-of-function mutations loosen the interaction between TCRαβ and CD3ζ. Similar to the activating mutations, pMHC binding reduces TCRαβ cohesion with CD3ζ. This event occurs prior to CD3ζ phosphorylation and at 0°C. Moreover, we demonstrate that soluble monovalent pMHC alone induces signaling and reduces TCRαβ cohesion with CD3ζ in membrane-bound or solubilised TCR-CD3. Our data provide compelling evidence that pMHC binding suffices to activate allosteric changes propagating from TCRαβ to the CD3 subunits, reconfiguring interchain transmembrane region interactions. These dynamic modifications could change the arrangement of TCR-CD3 boundary lipids to license CD3ζ phosphorylation and initiate signal propagation.

INTRODUCTION

T cell antigen receptor (TCR-CD3) signaling drives thymocyte maturation and T cell responses upon recognition of highly polymorphic major histocompatibility complex (MHC) proteins presenting myriad of short peptides (p) that originated from the degradation of self and foreign proteins (Stritesky et al., 2012). Despite high physical and chemical diversity in binding interfaces, TCR-CD3 ensures responses of exceptional specificity and sensitivity (Davis et al., 2007), with weak affinity (0.1–100 μM) and short half-life ($t_{1/2}$) (<0.5 to several seconds) (Aleksic et al., 2012; Cole et al., 2007; Stone et al., 2009). To accomplish this task, TCR-CD3 uses a clonally distributed αβ disulfide-linked dimer (TCR) with immunoglobulin (Ig)-like variable domains Vα and Vβ. VαVβ forms a pMHC binding site of six loops homologous to antibody complementarity determining regions (CDRs) 1, 2, and 3 (Garboczi et al., 1996; Garcia et al., 1996). Germline-encoded CDR1 and CDR2 have limited variability, whereas CDR3s are hypervariable. VαVβ orientates diagonally relative to the long axis of the peptide-binding groove (Garboczi et al., 1996; Garcia et al., 1996), with CDR3s contacting mainly the peptide and CDR1s and CDR2s contacting mainly the MHC (Baker et al., 2012; Garcia et al., 2012; Marrack et al., 2008). Vα and Vβ are connected to Ig-like constant domains, namely, Cα and Cβ, that are linked to the transmembrane regions (TMRs) through a stalk, called connecting peptide (CP). pMHC binding is signaled intracellularly by four non-covalently associated subunits (γ, δ, ε, and ζ), called CD3, organized into three dimers, namely, γε, δε and ζζ, with the latter disulphide linked (Call et al., 2002). ε, γ, and δ exhibit an Ig-like extracellular domain (ECD) connected to TMRs by short CPs, whereas ζ features a ≈10-residue-long ECD. A recent TCR-CD3 cryoelectron microscopy (cryo-EM) structure at 3.7 Å

This is an open access article under the CC BY license (http://creativecommons.org/licenses/by/4.0/).

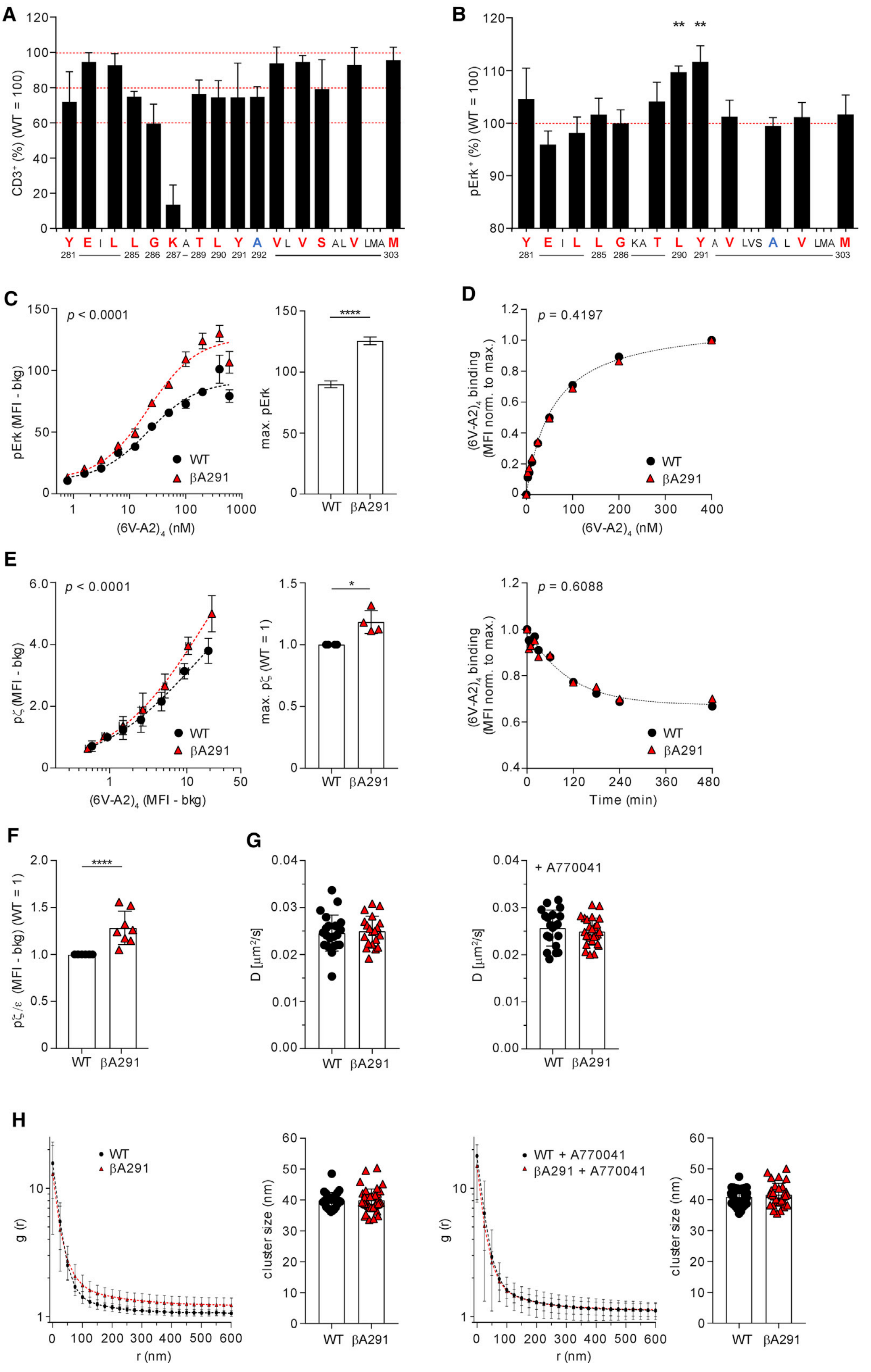

(legend on next page)

(Dong et al., 2019) reconciles with mutational and nuclear magnetic resonance (NMR) studies (Call et al., 2002; He et al., 2015; Mariuzza et al., 2020; Natarajan et al., 2016) but reveals other features. VαVβ projects forward with Cα interfacing with CD3δ ECD, Cβ interfacing with both CD3γε and CD3δ ECDs, and CD3γ and CD3ε (of δε) ECDs contacting each other. The TMRs of ζζ ($\zeta_1\zeta_2$) and αβ interact with each other, δε contacts α and ζ_1, and γε contacts β and ζ_2. The highly interlaced structure suggests a mutualistic contribution of each dimer to TCR-CD3 topology and cohesion. The intrinsically disordered intracellular tails of ε, γ, δ ,and ζ, invisible in the cryo-EM structure, contain immunoreceptor tyrosine-based activation motifs (ITAMs) that become phosphorylated by constitutively active Lck kinase (Nika et al., 2010) within $\leq$1 s after pMHC binding (Acuto et al., 2008; Huse et al., 2007). The tails are anchored to the plasma membrane (PM) by basic amino acid residues and ITAM tyrosines that interact with negatively charged lipids and hydrophobic lipid core, respectively (Deford-Watts et al., 2009; Xu et al., 2008), perhaps preventing ITAM access of unliganded receptors by Lck. Early studies suggested that binding of agonist anti-CD3 antibody (Ab) induces conformational changes in TCR-CD3 that expose CD3 cytoplasmic tails (Gil et al., 2002). However, crystallographic studies of TCRαβ ECD found extensive conformational changes in CDRs when bound to pMHC (Baker et al., 2012; Garcia et al., 2012) but no unambiguous or consistent changes beyond the TCRαβ binding site. This finding led to suggest TCR-CD3 signaling models independent of conformational changes or in which pMHC binding was insufficient to induce conformational changes. These models posited that signaling is induced by TCR-CD3 clustering (Cochran et al., 2001; Yokosuka et al., 2005), co-receptors (CD8/CD4) (Delon et al., 1998), or segregation of CD45 tyrosine phosphatase (Davis and van der Merwe, 2006). Alternatively, mechanosensing-based models suggested that force generated by PM movements acts on pMHC-bound TCR-CD3 to induce conformational changes and signaling (Kim et al., 2009; Liu et al., 2014). It was also proposed that TCR-CD3 clustering by pre-existing pMHC dimers induces conformational changes in CD3ε, but not directly in TCRαβ (Gil et al., 2002; Minguet et al., 2007). Nevertheless, one crystal structure (Kjer-Nielsen et al., 2003) and a fluorescence-based study (Beddoe et al., 2009) showed that pMHC induced conformational changes in Cα. Deuterium exchange (Hawse et al., 2012) and NMR investigations (Natarajan et al., 2017; Rangarajan et al., 2018) inferred changes in conformational dynamics of soluble TCRαβ ECD when bound to pMHC. These changes mapped to where Cα and Cβ interface with ECDs of CD3 subunits (He et al., 2015; Natarajan et al., 2016). These studies could not rule in or out models proposed thus far nor did they prove that allosteric effects propagate from αβ to the CD3 subunits for signaling to occur. To challenge this impasse, we conceived a genetic perturbation analysis to help discriminate between models requiring or not molecular flexibility (i.e., conformational changes). Toward this goal, we questioned the functional role of αβ TMR that establishes a key physical connection between the pMHC-binding module and the CD3 signaling subunits. If TMRs are only required for TCR-CD3 solvation within the lipid bilayer and quaternary structure topology, TMR mutations should not change TCR-CD3 intrinsic signaling capability. In contrast, this could happen in mechanisms based on allosteric interaction or force. We gathered compelling evidence for TMR mutations in TCRβ and CD3ζ that loosen quaternary structure cohesion and surprisingly augment signaling output. We also found that soluble monomeric pMHC agonists reduce TCR-CD3 quaternary structure cohesion and induce signal transduction, independently of co-receptor, clustering, or force. We propose that allosteric activation of TCR-CD3 by pMHC binding is the prime mover of T cell activation.

RESULTS

Gain-of-function mutations in β TMR

To question whether structural alterations in αβ TMR affected signaling, we used 1G4, an HLA-A2-restricted TCR specific for the 157-165 peptide from the NY-ESO-1 tumor antigen (Chen et al., 2005). Most residues of β TMR were individually replaced by alanine or leucine and the corresponding mutants tested for reconstituting TCR-CD3 surface expression in TCRβ-deficient Jurkat cells (J31.13) (Figure 1A). As reported earlier, βK287 mutation drastically reduced TCR-CD3 surface expression (Alcover et al., 1990). However, alanine substitutions at βY281, βL285, βG286, βT289, βL290, βY291, and βS296 and leucine at βA292 showed only a ≈20%–40% decrease of surface expression. Next, the majority of mutants showing 0%–40% reduction of surface expression was co-expressed together with wild-type (WT) 1G4 TCRα in J31.13, and Erk activation (pErk) was monitored after stimulation with 6V-HLA-A2 tetramer $(6V\text{-}A2)_4$ (Figure 1B). Although no mutation significantly reduced Erk activation, both βA290 and βA291 significantly increased pErk. A gain of function was unexpected, even more so as βA290 and βA291 reduced TCR-CD3 surface expression (data in Figure 1B are not normalized for TCR-CD3 surface expression).

Figure 1. Gain-of-function mutations in β TMR

(A) CD3 surface expression of 1G4-WT and mutants. x ± SD of $CD3^+$ cells, n = 3–8. Ala (red) and Leu (blue) substitution.

(B) pErk response of 1G4-WT and β mutants stimulated with $(6V\text{-}A2)_4$. x ± SEM of $pErk^+$ cells; n = 3–6; unpaired t test, p = 0.0011 (βA290), p = 0.0092 (βA291).

(C) pErk response of $CD8^-$ J76 1G4-WT and 1G4-βA291 stimulated with $(6V\text{-}A2)_4$. Left, non-linear regression fit of $(6V\text{-}A2)_4$ nM versus pErk MFI, n = 3, R^2 = 0.82 (WT), 0.89 (βA291); EC_{50} = 19.5 ± 5.5 (WT), 19.0 ± 3.1 (βA291). Right, x ± SD of max. pErk, n = 3, F-test p < 0.0001. See also Figure S1E.

(D) $(6V\text{-}A2)_4$ binding to 1G4-WT or 1G4-βA291. Top, $(6V\text{-}A2)_4$ dose-dependent association, n = 3, non-linear regression fit, R^2 = 0.98 (WT), 0.97 (βA291), F-test (ns). Bottom, $(6V\text{-}A2)_4$ dissociation rate, n = 5, non-linear regression fit, R^2 = 0.84 (WT), 0.72 (βA291), F-test (ns).

(E) pζ response of J76 1G4-WT or 1G4-βA291 stimulated with $(6V\text{-}A2)_4$. Left, non-linear regression fit of $(6V\text{-}A2)_4$ MFI versus pζ MFI, n = 3, R^2 = 0.95 (WT), 0.96 (βA291). Right, x ± SD of max. pζ, n = 4, unpaired t test p = 0.0078. See also Figure S1F.

(F) Basal pζ in J76 1G4-WT or 1G4-βA291. pζ MFI normalized to surface CD3 MFI, n = 8, unpaired t test p < 0.0001. See also Figures S1H and S1I.

(G) FRAP of 1G4-WT or 1G4-βA291 treated (right) or not (left) with A770041. x ± SD of diffusion coefficient, D (μm^2/s), n ≥ 20 cells, t test (ns).

(H) Lateral distribution by dSTORM of 1G4-WT or 1G4-βA291 treated (right) or not (left) with A770041. Plots represent pair auto-correlation analysis (g), x ± SD of ≥ 25 cells. Histograms show DBSCAN cluster analysis, x ± SD of cluster size per cell, t test (ns).

βA291 heightens basal and ligand-induced signaling

To validate this apparently paradoxical observation, we focused on βA291 and modified the experimental set up to improve data robustness. Thus, α and β of 1G4 were expressed as a single self-cleavable polypeptide (Figure S1A) from a doxycycline (dox)-inducible promoter in a TCRαβ-deficient Jurkat cell line (J76). J76 expressed maximum levels of surface TCR-CD3 after 16–18 h of dox treatment and were tested soon after to reduce the potential risk of phenotypic drift of cells expressing 1G4 carrying βA291 (hereafter, referred to as 1G4-βA291). As in 31.13 cells, 1G4-βA291 expressed in J76 showed reduced surface expression (≈30%) (*cf.* Figure 1A with Figure S1B). However, in most experiments, we lowered the dox concentration when inducing 1G4-WT to reduce the difference in surface expression with 1G4-βA291 (to <5%) (Figure S1C). Moreover, in most flow cytometry analyses, J76 expressing 1G4-WT or mutant were barcoded by labeling with CellTrace violet, mixed before stimulation, and analyzed simultaneously. These stratagems simplified and made more robust the computation of differences in signaling output between the WT and mutant. Erk activation was retained as a sensitive and reliable readout of TCR-CD3 signal transduction and propagation as it depends on a cascade of early signaling steps, including ITAM phosphorylation, ZAP-70 activation, LAT signalosome assembly, and PLCγ1 activation that generates IP_3 (for intracellular [Ca^{2+}] increase) and DAG required for Ras activation by Ras-GRP (Acuto et al., 2008). Titration of $(6V\text{-}A2)_4$ showed a shift in pErk response by 1G4-βA291 toward higher sensitivity and revealed significantly higher Erk activation (Figure 1C). This result was not due to a higher Erk activation ceiling in 1G4-βA291-expressing cells (Figure S1D) nor to augmented binding of $(6V\text{-}A2)_4$ to 1G4-βA291 (Figure 1D, top and bottom panels), but it was consistent with the dose-response plot showing unchanged EC_{50} between 1G4-βA291 and 1G4-WT (Figure 1C and STAR Methods for computation). The higher maximal response of 1G4-βA291 was compatible with a faster proofreading rate (k_p) for a receptor operating in a kinetic proofreading regimen (McKeithan, 1995). Indeed, fitting the data of Figure 1C into a minimal model of kinetic proofreading (Dushek et al., 2011) showed that k_p for 1G4-βA291 was considerably higher than that for 1G4-WT (Figure S1E), consistent with βA291 enhancing TCR-CD3 intrinsic signaling capability (i.e., higher ligand potency). The gain of function was observed in CD8-deficient J76 (Figure 1C), ruling out that the βA291 enhanced the TCR-CD3 interaction with co-receptor. Augmented signaling was also evident for ζ phosphorylation (pζ) (Figures 1E and S1F), the earliest intracellular signaling event. Remarkably, anti-CD3ε (UCHT1) Ab stimulation of 1G4-βA291 also heightened pζ (Figure S1G), a triggering modality that by-passes pMHC binding, further supporting that βA291 enhanced TCR-CD3 signaling. These data suggested that βA291 might increase constitutive TCR-CD3 signaling detected by measuring pζ in non-stimulated cells. Indeed, basal pζ was significantly higher in cells expressing 1G4-βA291 as compared to 1G4-WT (Figures 1F and S1H), and it was TCR signal specific as it disappeared after treatment by A770041 (Stachlewitz et al., 2005), a highly specific Lck inhibitor (Figure S1I). We then asked if βA291 increased signaling by influencing TCR-CD3 lateral diffusion and/or distribution. However, fluorescence recovery after photo-bleaching (FRAP) found no significant difference in the diffusion coefficient (D) between 1G4-βA291 and 1G4-WT (Figure 1G, left panel), which remained unchanged after A770041 treatment (Figure 1G, right panel). Direct stochastic optical reconstruction microscopy (dSTORM) super-resolution microscopy found no statistically significant difference in the cluster size distribution formed by 1G4-βA291 and 1G4-WT (histograms in Figure 1H). Although not statistically significant, the reproducible small increase of larger cluster frequency for 1G4-βA291 disappeared after A770041 treatment (*cf.* auto-correlation function plots in left and right panels of Figure 1H), indicating it to be secondary to 1G4-βA291 heightened basal signaling (Figure 1F) rather than βA291 causing it. Finally, we questioned the potential cause(s) of mildly reduced 1G4-βA291 surface expression. We excluded that βA291 reduced β protein expression (Figure S1J) and considered that heightened basal signaling might decrease receptor surface expression by increasing its downregulation rate. However, exposure to A770041 for several hours increased surface expression of both 1G4-βA291 and 1G4-WT in a similar proportion (≈20%) but did not reduce their difference (Figure S1K). These data led us to consider if βA291 modified the stability of TCR-CD3 quaternary structure that could reduce export to the PM due to increased negative triage by protein quality-control systems (Feige and Hendershot, 2013) of mutant versus WT.

βY291 contribution to TCR-CD3 quaternary structure cohesion

Non-ionic detergents used at a high concentration to quantitatively extract TCR-CD3 can dissociate TCRαβ from the CD3 modules (Testi et al., 1989). Presumably, this can be attributed to extensive substitution of natural boundary lipids by the detergent, with possible interference with TMR inter-helical interactions critical for TCR-CD3 quaternary structure cohesion (Alcover et al., 1990; Call et al., 2002). However, 0.5% of the non-ionic detergent n-dodecyl-β-D-maltopyranoside (DDM) allows quantitative extraction of stoichiometrically intact TCR-CD3 (Swamy et al., 2008; Figure S2A). Thus, if βA291 altered TCRαβ cohesion with CD3 by unsettling TMR inter-helical interactions, 0.5% DDM extraction may show lower recovery of intact 1G4-βA291 with respect to 1G4-WT. Figure S2B illustrates the experimental set up of chemically probing TCR-CD3 cohesion by DDM that we named DDM stability assay (DSA) (see STAR Methods for details). Membrane solubilisation by 0.5% DDM and pull-down (PD) of total β (mostly associated with α; Alcover et al., 2018) by the β-hemagglutinin (HA) tag was followed by quantitative immunoblot (IB) for β (with anti-HA Ab) and for each CD3 subunit. Anti-HA IB identified three β isoforms (named β_1, β_2, and β_3; Figure 2A). β_3 was the endo-H-sensitive endoplasmic reticulum (ER)-resident β isoform (Figure S2C) that is assembled with α, γε, and δε but not with ζζ (Alcover et al., 2018), as confirmed by β_3 being undetected in CD3ζ PD (Figure S2D). β_1 and β_2 were both endo-H-resistant (Figure S2C), although β_2 was the only β isoform associated with ζζ (Figure S2D). Thus, to evaluate the effect of βA291 on TCR-CD3 complex cohesion, we used the IB signals of β isoforms, namely, ζζ and ε (which includes γε and δε). When ζ/β_2 was set equal to 1 for 1G4-WT (i.e., 100% recovery of intact TCR-CD3), reduced

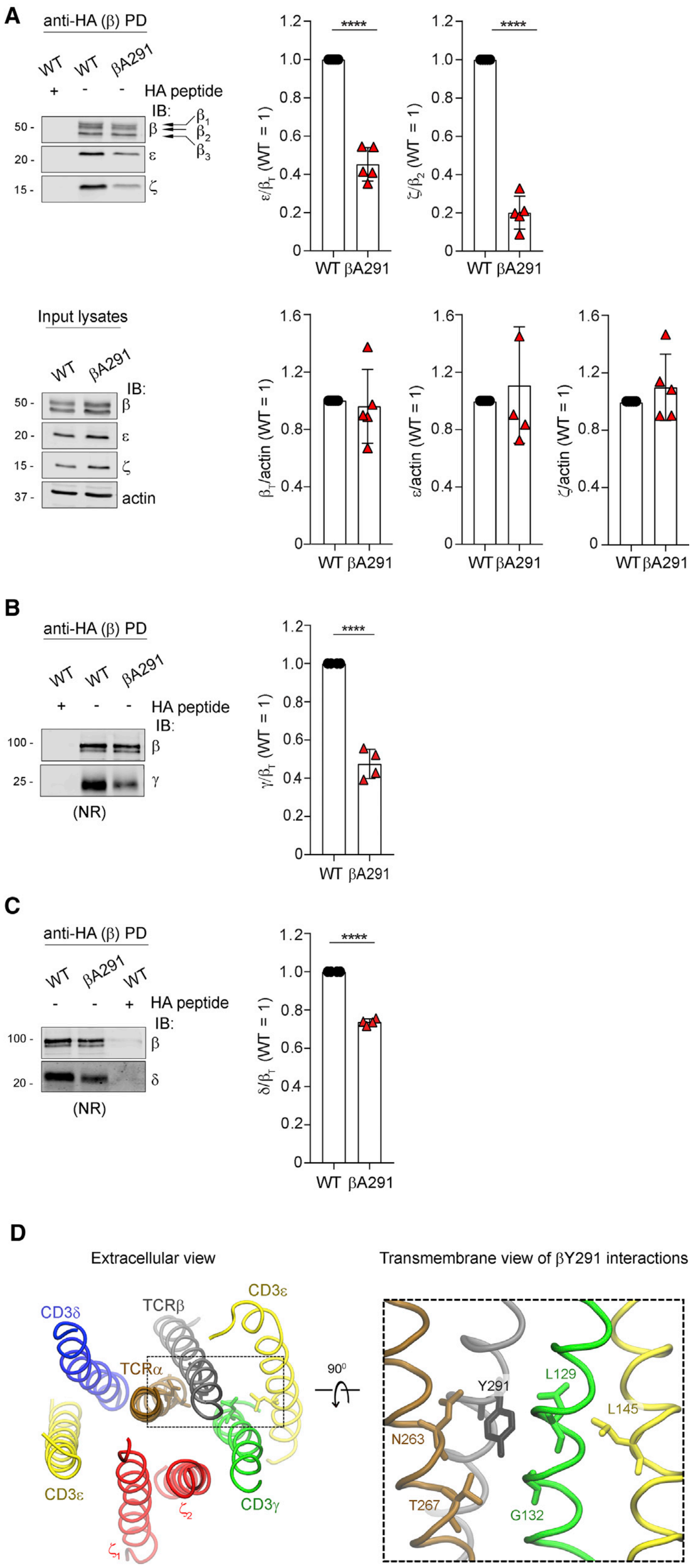

Figure 2. βY291 contribution to TCR-CD3 quaternary structure cohesion

(A) Anti-HA (β-HA) pull-down (PD), and IB of 1G4-WT or 1G4-βA291. Top panels: left, representative IB, arrows indicate β-isoforms; right, x ± SD of ε/β_T and ζ/β_2, n = 5, unpaired t test $p < 0.0001$. Bottom panels: input lysates, left, representative IB; right, x ± SD of β_T/actin, ε/actin and ζ/actin, n = 5, unpaired t test (ns).

(B) β-HA PD and IB of 1G4-WT or 1G4-βA291. NR (non-reducing) conditions. Left, representative IB. Right, x ± SD of γ/β_T, n = 4, unpaired t test $p < 0.0001$.

(C) β-HA PD and IB of 1G4-WT or 1G4-βA291. NR conditions. Left, representative IB. Right, x ± SD of δ/β_T, n = 4, unpaired t test $p < 0.0001$.

(D) All-atom MDS of TCR-CD3 TMRs. TCRα (ochre), TCRβ (gray), CD3δ (blue), CD3ε (yellow), CD3γ (green), ζ (red). Left, snapshot of TCR-CD3 TMRs. Right, βY291 interactions with TCR-CD3 TMRs. Liquorice sticks show significant contacts of βY291 with TCRα, CD3γ, and CD3ε. See also Figure S2H.

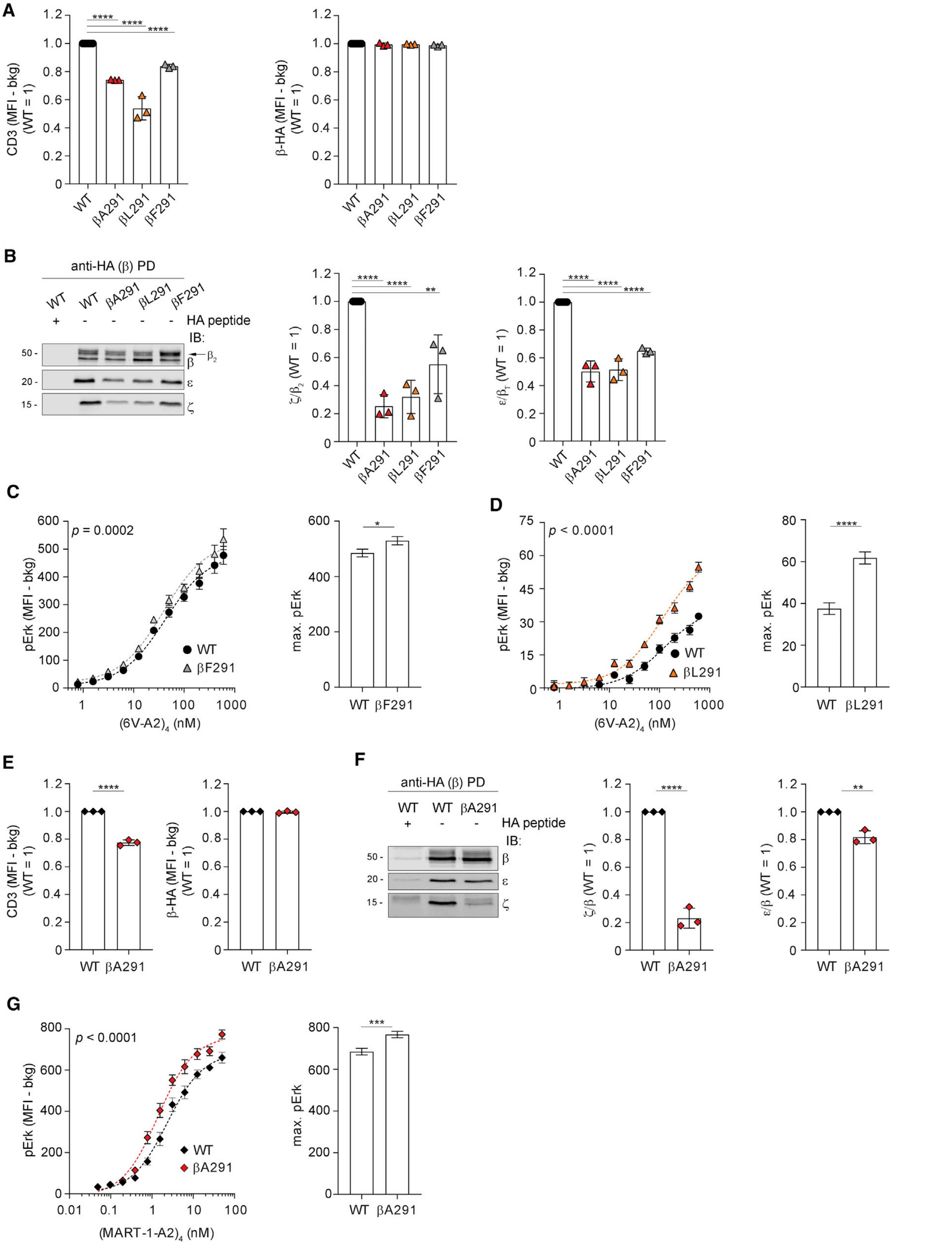

(legend on next page)

cohesion between ζζ and αβ should result in $\zeta/\beta_2 < 1$ (Figure S2B). The DSA showed that ζ/β_2 for 1G4-βA291 was 0.2, indicating only 20% recovery of intact TCR-CD3 (or 80% loss of ζζ recovery) after DDM solubilization (Figure 2A). To determine the effect of βA291 on γε and δε cohesion with αβ, we used instead the sum of β_1, β_2, and β_3 (total β (β_T) IB signals that represented cytoplasmic and PM αβ, of which most is associated with ε (Alcover et al., 2018). ε/β_T for 1G4-βA291 was 0.5, indicating 50% reduced recovery of ε (Figure 2A). These ratios did not change after A770041 treatment during dox induction of αβ expression (Figure S2E), excluding that reduced recovery concerned the pool of 1G4-βA291 with increased basal pζ (Figure 1F). The considerable reduction of ζ (80%) and ε (50%) recovery for 1G4-βA291 could not be the consequence of severance of the same magnitude of αβ from ζζ (or from δε and γε) before export to the PM and/or at the PM, as only 20%–30% reduction of TCR-CD3 expression was observed by flow cytometry and incompatible with increased pMHC-induced signaling. Indeed, when TCRαβ is no longer in contact with ζζ, TCRαβδεγε alone cannot be exported to the T cell surface (Figure S2F; Alcover et al., 2018). Therefore, in the PM natural lipid environment, βA291 only slightly perturbed TCR-CD3 quaternary structure cohesion (as the molecular dynamics simulations [MDSs] show, see below), moderately reducing surface expression. However, substitution of natural boundary lipids by DDM severely corroded TCR-CD3 cohesion in 1G4-βA291 and provoked partial physical detachment of ζ and ε from αβ during the solubilization. IB for γ and δ revealed that βA291 affected both γε and δε cohesion with the rest of the complex, although asymmetrically, as it reduced γε and δε recovery of 40% and 10%, respectively (Figures 2B and 2C). In the cryo-EM structure, βY291 (note that Dong et al. 2019 refer to βY291 as βY292) contacts mostly γε, and therefore, βA291 can be expected to primarily affect the interaction between αβ and γε in accordance with the DSA. However, βY291 makes no contacts with ζζ and δε (Dong et al., 2019) (see MDS below). Therefore, the DSA revealed a more complex picture, with βA291 presumably indirectly affecting the interaction of both ζζ and δε with the rest of the complex. To further understand the structural role of βA291, we used the TMR atomic coordinates of the cryo-EM structure of the TCR-CD3 octamer (PDB: 6JXR) (Dong et al., 2019) to carry out all-atom MDSs with βWT and βA291 in an asymmetric lipid bilayer, mimicking the lipid environment of TCR-CD3, thus adding dynamical insight into TCR-CD3 cohesion. Simulations for 1,250 ns confirmed considerable contacts of βWT with ε (of γε), γ, α, and ζζ but not with δε (Figures 2D and S2G) and revealed one new contact of β with ζζ as well as significant reduction in six β contacts with ε (γε), five with γ, and four with α (Figure S2G). Specifically, significant contacts were observed of βY291 with αN263, αT267, γL129, γG132, and εL145 (Figures 2D, right panel, and S2H) and with γV133 and γI136, although the latter two were not significant (Figure S2H). No contacts of βY291 with ζζ were seen (Figure S2G). Simulations of the TMR octamer carrying βA291 indicated new and augmented contacts of β with ε (γε) and γ (Figure S2I). In addition, although βA291 still contacted γL129, it completely lost interaction with γG132, γV133, and γI136 (Figure S2J, middle panel). Likely, these changes were secondary to spatial re-adjustments due to the loss of the bulky tyrosine side chain. No contacts of βA291 with ζζ were observed. Overall, the simulations suggested that βA291 reshuffled contacts with γε, with the net effect of increasing local compaction (Figure S2K), as indicated by a stabilization of their α helix crossing angle (Figure S2L). This result seemed to contradict the DSA data of βA291 severely affecting the ζζ interaction with the rest of the complex. Although a 1,250-ns timescale is relatively long for all-atom simulations of membrane proteins, it might be insufficient to capture re-adjustments of interchain contacts that possibly occur at larger timescales. βA291 might affect the role of interfacial lipids in cementing α helix interactions (Gupta et al., 2017) that, when challenged with DDM, might cause crumbling of TMR cohesion in the mutant, despite augmented compaction by βA291 elsewhere. However, reduced export to the PM was a good indicator that βA291 (and other β and ζTMR mutants, see below) promoted some instability of the complex, causing dynamical exposure of hydrophobic site and/or retention signals, detected and negatively triaged by protein quality-control systems (Feige and Hendershot, 2013). Comprehensively, these data suggested a positive correlation between TCR-CD3 reduced quaternary structure cohesion and activation of signal transduction.

Loosening ζ association enhances signaling

To corroborate this hypothesis, we investigated the phenotype of additional mutations in β and ζ TMRs. We found that similar to βA291, also βF291 and βL291 mildly reduced TCR-CD3 surface expression, despite no decrease in β expression (Figure 3A). Both mutations reduced the interaction of β with ζ and ε (Figure 3B)

Figure 3. Loosening ζ association enhances signaling

(A) TCR-CD3 expression of 1G4-WT, 1G4-βA291, 1G4-βL291, and 1G4-βF291 in $CD8^-$ J76. Left, x ± SEM of CD3 MFI in HA^{low}gate, n = 3, unpaired t test $p < 0.0001$. Right, x ± SEM of β-HA MFI in HA^{low}gate, n = 3, t test (ns).

(B) β-HA PD and IB of 1G4-WT or 1G4-β mutants. Left, representative IB, the arrow indicates β_2 isoform. Middle, x ± SD of ζ/β_2, n = 3, unpaired t test WT versus βA291, WT versus βL291 $p < 0.0001$, WT versus βF291 $p < 0.01$. Right, x ± SD of ε/β_T, n = 3, unpaired t test $p < 0.0001$.

(C) pErk response of $CD8^-$ J76 1G4-WT or 1G4-βF291 stimulated with $(6V\text{-}A2)_4$. Left, non-linear regression fit of $(6V\text{-}A2)_4$ nM versus pErk MFI, n = 3, $R^2 = 0.915$ (WT), 0.910 (βF291). Right, x ± SD of max. pErk, n = 3, F-test $p < 0.05$. See also Figure S3A (left).

(D) pErk response of $CD8^-$ J76 1G4-WT or 1G4-βL291 stimulated with $(6V\text{-}A2)_4$. Left, non-linear regression fit of $(6V\text{-}A2)_4$ nM versus pErk MFI, n = 3, $R^2 = 0.827$ (WT), 0.910 (βL291). Right, x ± SD of max. pErk, n = 3, F-test $p < 0.0001$. See also Figure S3A (right).

(E) TCR-CD3 expression in $CD8^-$ J76 2H5-WT or 2H5-βA291. Left, x ± SEM of CD3 MFI in HA^{low}gate, n = 3, unpaired t test $p < 0.0001$. Right, x ± SEM of β-HA MFI in HA^{low}gate, n = 3, t test (ns).

(F) β-HA PD and IB of 2H5-WT or 2H5-βA291. Left, representative IB. Middle, x ± SD of ζ/β, n = 3, unpaired t test $p < 0.0001$. Right, x ± SD of ε/β, n = 3, unpaired t test $p < 0.01$.

(G) pErk response of $CD8^-$ J76 2H5-WT or 2H5-βA291 stimulated with $(MART\text{-}1\text{-}A2)_4$. Left, non-linear regression fit of $(MART\text{-}1\text{-}A2)_4$ nM versus pErk MFI, n = 3, $R^2 = 0.94$ (WT), 0.94 (βA291). Right, x ± SD of max. pErk, n = 3, F-test $p < 0.001$. See also Figure S3B.

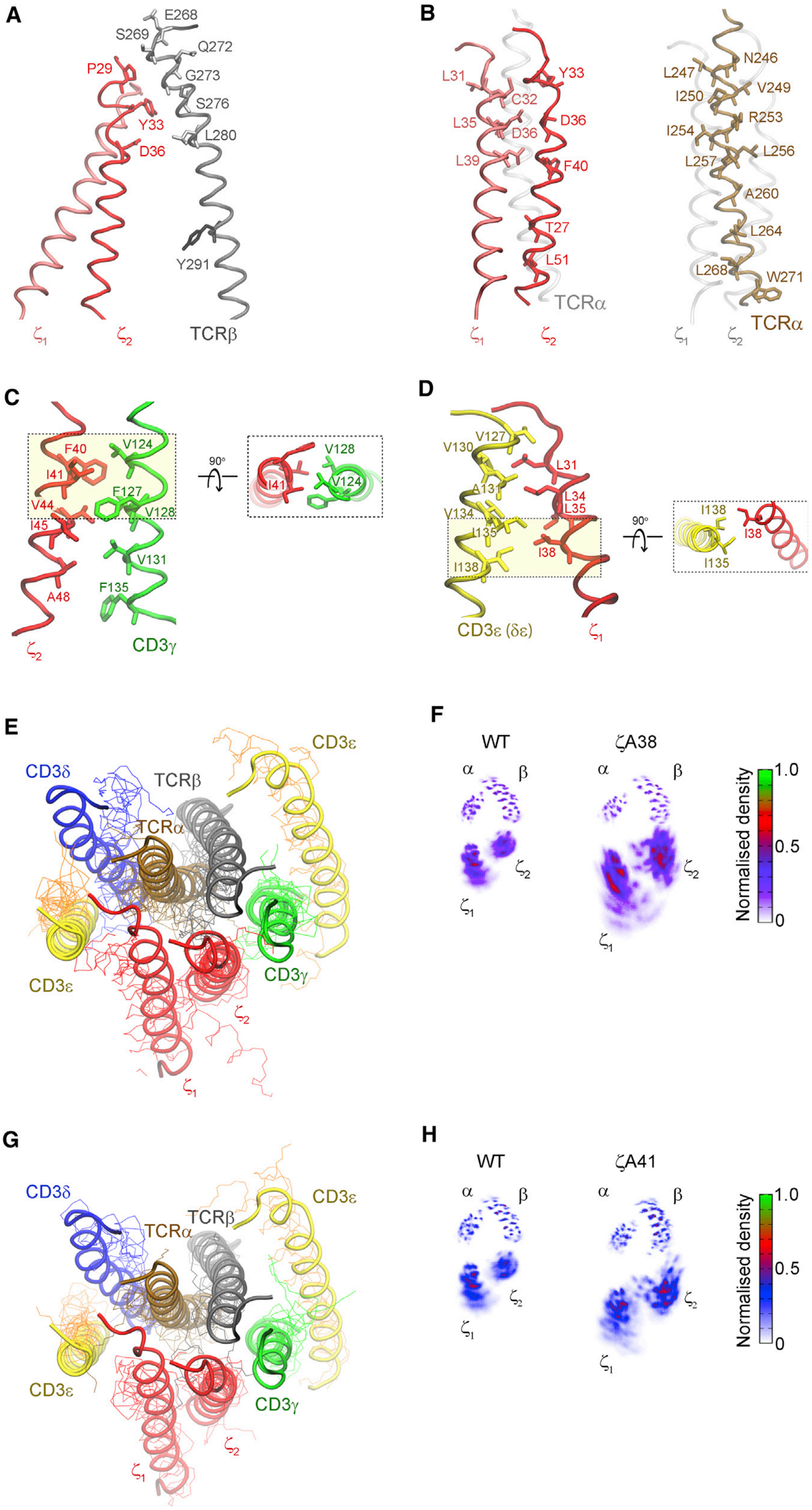

(legend on next page)

and augmented pErk maximal response to $(6V\text{-}A2)_4$ (Figures 3C and 3D), whose binding remained unchanged (Figure S3A). These three readouts ranked according to βL291 ≥ βA291 > βF291 > WT, presumably reflecting conservative or non-conservative replacements, and hence indicating a direct correlation between increased quaternary structure loosening and heightened signaling. We then tested the effect of βA291 in 2H5, a HLA-A2-restricted TCR specific for the MART-1 tumor antigen (Circosta et al., 2009). Similar to 1G4-βA291, 2H5-βA291 showed reduced surface expression (Figure 3E) and TCR-CD3 cohesion (Figure 3F) and augmented pErk for equal $(MART\text{-}1\text{-}A2)_4$ binding (Figures 3G and S3B). Conversely, mutation of β TMR residues not involved in critical contacts (Dong et al., 2019), such as βA293 and βA303, showed no significant change of 1G4 surface expression (Figure S3C) or TCR-CD3 cohesion (Figures S3D and S3E) and no increase in $(6V\text{-}A2)_4$-induced pErk (Figures S3F and S3G).

βY291 did not contact ζ, but its mutation augmented basal (Figure 1F) and ligand-induced pζ (Figure 1E) and signal propagation. This was reminiscent of an allosteric interaction revealed by mutations (Changeux and Christopoulos, 2016; Volkman et al., 2001), e.g., βA291 inducing local re-adjustments but also distal functional effects, such as favoring exposure of ζ cytosolic tail to active-Lck. To investigate this possibility, we tested whether mutations in ζ TMR residues susceptible to loosen ζζ contacts with subunits other than αβ phenocopied mutations at βY291. TCR-CD3 cryo-EM structure and MDS indicated that ζ_1 and ζ_2 TMRs contacted only the N-terminal moiety of β TMR (Figures 4A and S4A) and α TMR throughout (Figures 4B and S4B). However, ζ_2 and ζ_1 also contacted γ (Figures 4C and S4C) and ε (of the δε) (Figures 4D and S4D), respectively. Specifically, MDS revealed that ζ_1I38 contacted two residues of ε (of δε) (Figures 4D and S4E, left panel) and ζ_2I41 contacted two residues of γ (Figures 4C and S4F, left panel), whereas ζ_2I38 and ζ_1I41 bulged toward the membrane lipids and made no contact with the complex. Thus, ζ_1I38 and ζ_2I41 were deemed capable of partially disturbing ζ_1 and ζ_2 interactions with ε (of δε) and γ but perhaps not with αβ. To verify this prediction, 1,250 ns all-atom simulations of TCR-CD3 octamer TMRs composed of ζ WT and ζA41 and ζA38 mutants were carried out. At the end of the simulations, snapshot alignment of the mutated and WT TMRs showed distortion in the contacts of ζ_1 with δε (Figure 4E) and ζ_2 with γ (Figure 4G). As a consequence, ζζ containing ζ_1A38 (3 out of 3 simulations) or ζ_2A41 (2 out of 3 simulations) increased fluctuation relative to αβ as compared to ζζ WT. This can be appreciated from the average spatial distribution plots of the Cα atoms of ζζ relative to the Cα atoms of αβ that showed broader density for both mutants (Figures 4F and 4H), although this was more pronounced for ζ_1A38. These results were indicative of ζA38 and ζA41 increasing ζζ flexibility relative to αβ. Both mutants maintained some ζζ contacts with the rest of the complex (Figures S4G–S4N). These results prompted us to test if, similar to the βA291 mutations, these ζ mutations also showed reduced surface expression, complex cohesion by DSA, and enhanced signaling. The data showed that ζA38 or ζA41 reduced 1G4 surface expression by ≈30%, for similar ζ expression (Figure 5A). The DSA showed that ζA38 and ζA41 reduced the ζ/β_2 ratio to ≈0.05 and ≈0.25 (95% and 75% loss of ζ recovery) respectively, without apparently affecting ε cohesion with αβ. (Figures 5B and S5A). Thus, the DSA agreed with loosening of the ζζ interaction with αβ as predicted by the atomistic simulations. Perhaps, weakening direct interactions with CD3 TMRs and causing higher ζζ mobility, ζA38 and ζA41 indirectly weakened the ζζ interaction with αβ as well, as revealed by DDM extraction. Simulations of larger timescales may provide clearer insights into dynamic alterations of ζζ by βA291 producing direct effects on γε and later indirect ones on ζζ. Importantly, similar to βA291, both ζ mutations conferred to 1G4 heightened the pErk response to $(6V\text{-}A2)_4$, with a higher maximum than that of 1G4-WT (Figures 5C and 5D) for equal $(6V\text{-}A2)_4$ binding (Figures S5B and S5C). We concluded that reduced cohesion between αβ and ζζ caused heightened signaling, rather than the mutations of βY291 per se. This conclusion strengthened the idea that reducing TCR-CD3 cohesion populated the active signaling state of TCR-CD3, i.e., it lowered the activation energy between two presumed functional states, inactive and active, with the latter initiating transmembrane signaling. These data made it unlikely that TCR-CD3 TMRs are just structural elements required for TCR-CD3 membrane solvation and architecture, as conformational change-independent models would imply. Rather, by analogy with allosterically regulated proteins that can be switched on or off by mutations distal from their active site(s) (Changeux and Christopoulos, 2016; Volkman et al., 2001) and considering recent NMR studies (He et al., 2020; Natarajan et al., 2016, 2017; Rangarajan et al., 2018), our data suggested that pMHC binding activated an allosteric cascade that loosened TCR-CD3 cohesion, including interactions with ζζ TMRs serving

Figure 4. Loosening ζ association enhances signaling

(A) Snapshot from all-atom MDS of TCR-CD3 TMRs. Contacts between TCRβ (gray), ζ_1 (light red), and ζ_2 (dark red) TMRs. See also Figure S4A. βY291 is represented as liquorice stick for reference and does not contact ζζ.

(B) Snapshot from all-atom MDS of TCR-CD3 TMRs. Left, ζ_1 (light red) and ζ_2 (dark red) residues contacting TCRα TMR (in transparency). Right, TCRα (ochre) residues contacting ζ_1 and ζ_2 TMRs (in transparency). See also Figure S4B.

(C) Snapshot from all-atom MDS of TCR-CD3 TMRs. Left, contacts between ζ_2 (red) and CD3γ (green) TMRs. Right, top view of ζ_2I41 (red) contacts with CD3γ (green). See also Figures S4C and S4F.

(D) Snapshot from all-atom MDS of TCR-CD3 TMRs. Left, contacts between ζ_1 (red) and CD3ε (δε) (yellow) TMRs. Right, top view of ζ_1I38 (red) contacts with CD3ε (δε) (yellow). See also Figures S4D and S4E.

(E) Snapshot from all-atom MDS of TCR-CD3 TMRs carrying ζA38 (lines) aligned to ζWT (cartoon) at the end of 1,250 ns MDS. TCRα (ochre), TCRβ (gray), CD3δ (blue), CD3ε (yellow), CD3γ (green), ζ (red). See also Figures S4G–S4J.

(F) Normalized spatial distributions of the Cα atoms of ζζ relative to the Cα atoms of TCRαβ in ζWT and ζA38.

(G) Snapshot from all-atom MDS of TCR-CD3 TMRs carrying ζA41 (lines) aligned to ζWT (cartoon) at the end of 1,250 ns MDS. TCRα (ochre), TCRβ (gray), CD3δ (blue), CD3ε (yellow), CD3γ (green), ζ (red). See also Figures S4K–S4N.

(H) Normalized spatial distributions of the Cα atoms of ζζ relative to the Cα atoms of TCRαβ in ζWT and ζA41.

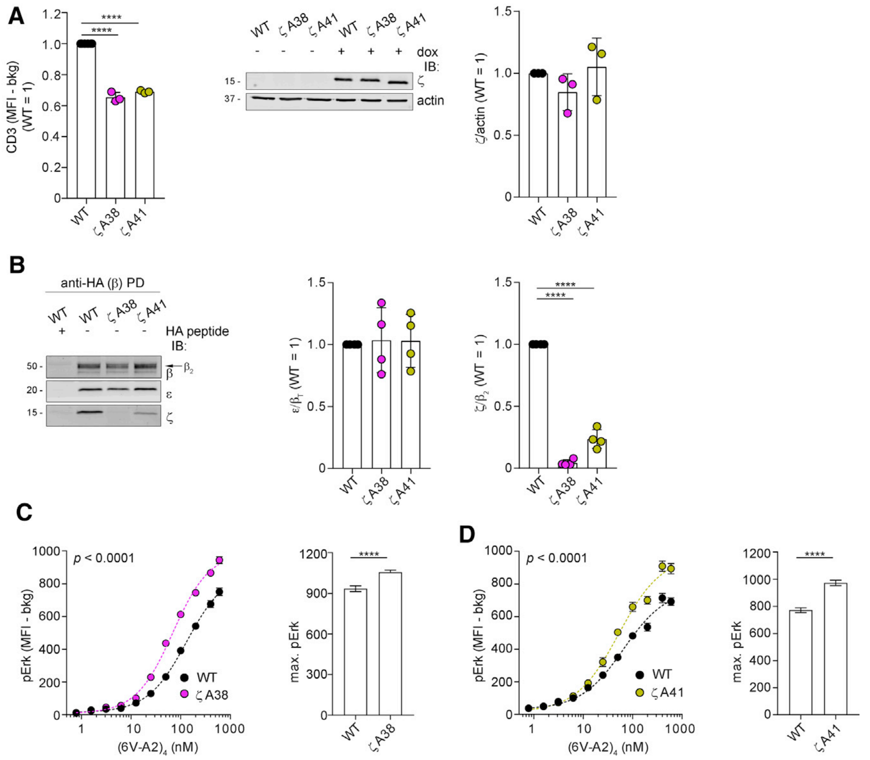

Figure 5. Loosening ζ association enhances signaling

(A) TCR-CD3 expression in J76-1G4WT-ζKO expressing ζWT or ζA38 or ζA41. Left, x ± SEM of CD3 MFI in HA^{low} gate, n = 3, unpaired t test $p < 0.0001$. Middle, representative IB. Right, x ± SD of ζ/actin, n = 3, unpaired t test (ns).

(B) β-HA PD and IB of 1G4-WT carrying ζWT or ζA38 or ζA41. Left, representative IB, the arrow indicates β_2 isoform. Middle, x ± SD of ε/β_T, n = 4, unpaired t test (ns). Right, x ± SD of ζ/β_2, n = 4, unpaired t test $p < 0.0001$. See also Figure S5A.

(C) pErk response of J76-1G4WT-ζKO expressing ζWT or ζA38 stimulated with $(6V\text{-}A2)_4$. Left, non-linear regression fit of $(6V\text{-}A2)_4$ nM versus pErk MFI, n = 3, R^2 = 0.98 (WT), 0.99 (ζA38). Right, x ± SD of max. pErk, n = 3, F-test $p < 0.0001$. See also Figure S5B.

(D) pErk response of J76-1G4WT-ζKO expressing ζWT or ζA41 stimulated with $(6V\text{-}A2)_4$. Left, non-linear regression fit of $(6V\text{-}A2)_4$ nM versus pErk MFI, n = 3, R^2 = 0.96 (WT), 0.97 (ζA41). Right, x ± SD of max. pErk, n = 3, F-test $p < 0.0001$. See also Figure S5C.

as a second-to-last relay before licensing ζ ITAM phosphorylation. These considerations prompted us to investigate this possibility.

pMHC tetramer binding loosens αβ association with ζ

Conformational changes produced by pMHC binding may have distal effects that reduce contacts of CαCβ with δε and/or γε ECDs, eventually propagating to TMR contacts, including ζζ TMR, and resulting in reduced TCR-CD3 cohesion. If correct, the DSA should show reduced recovery of ζζ in ligand-engaged TCR-CD3 versus unengaged TCR-CD3. To test this idea, we aimed to capture DDM-solubilized pMHC-engaged TCR-CD3 and compare subunit recovery with unengaged TCR-CD3. Thus, 1G4-WT-expressing J76 was briefly stimulated with $(9V\text{-}A2)_4$, tetramerized with His-tagged streptavidin [$(9V\text{-}A2)_4$-His] (Figure S6A), ligand excess was removed, and cells rapidly solubilized with 0.5% DDM. Post-nuclear lysates were incubated with His-Cobalt beads for capturing $(9V\text{-}A2)_4$-His-bound 1G4. This experimental setting failed to capture sufficient $(9V\text{-}A2)_4$-His-bound TCR-CD3, likely because detergent solubilization interfered with the avidity gain due to tetramer-induced clustering in the membrane milieu, by making the dissociation rate of individual 9V-A2 in the tetramer closer to that of a 9V-A2 monomer alone (i.e., solution k_{off} of 0.33 s^{-1} at 25°C; Aleksic et al., 2010). To overcome this limitation, we initially used wtc51 (Irving et al., 2012), a 1G4 variant harboring four mutations in βCDR2 (Figure S6B) that confer a 15 nM diffusion constant (K_d) for NY-ESO-$1_{157\text{-}165}$-HLA-A2 (k_{off} of 0.0013s^{-1} at 25°C). Computational modeling showed that NY-ESO-$1_{157\text{-}165}$-HLA-A2 adopts a canonical orientation onto wtc51 VαVβ, which is almost indistinguishable from 1G4-WT (Figure S6B). $(9V\text{-}A2)_4$-His induced robust wtc51-mediated Erk activation (Figure 6A, left panel) and allowed specific capture of engaged wtc51 (Figure 6A, middle panel, lanes 2 and 4) to be compared with unliganded wtc51 isolated by anti-HA β PD (Figure 6A, middle panel, lanes 1 and 3). β_2 was the only isoform bound to $(9V\text{-}A2)_4$-His (Figure 6A, middle panel, lanes 2 and 4), consistent with it being the only one associated to ζ and present at the cell surface (Figure S2D). Therefore, the ζ/β_2 ratio was used to assess if pMHC binding had reduced cohesion of ζ within TCR-CD3 (Figure 6A, right panel). The data showed that ζ/β_2 in liganded wtc51 was 0.5 (Figure 6A, right panel), in agreement with pMHC binding causing relaxation of TCR-CD3 cohesion. This effect was independent of pζ, as identical results were obtained after A770041 treatment (Figure 6A middle and right panels). Allosteric interaction typically occurs in tens of μs to few ms (Volkman et al., 2001), similar to the timescale observed by NMR for pMHC binding to induce conformational changes in Cβ (Natarajan et al., 2017). As pMHC binding dwell times are of a much longer timescale (e.g., hundreds of ms to min), allosterically induced conformational changes should be observable at non-physiological lower temperatures. Consistently, almost identical reduction of the ζ/β_2 ratio was observed when $(9V\text{-}A2)_4$ was reacted with cells at 0°C (Figure 6B middle and right panels). To exclude that our observations were biased by the particular mutations introduced in βCDR2 and/or by the non-physiological affinity of wtc51, we used QM-α TCR, a 1G4 variant carrying mutations in αCDR2, βCDR2, and βCDR3 (Figure S6B; Irving et al., 2012), which confer a 140 nM K_d (k_{off}, 0.015 s^{-1} at 25°C) for NY-ESO-$1_{157\text{-}165}$-HLA-A2 (Irving et al., 2012), within the physiological range of TCR-pMHC binding affinity (Aleksic et al., 2012; Cole et al., 2007, 2017; Stone et al., 2009). Molecular modeling showed that

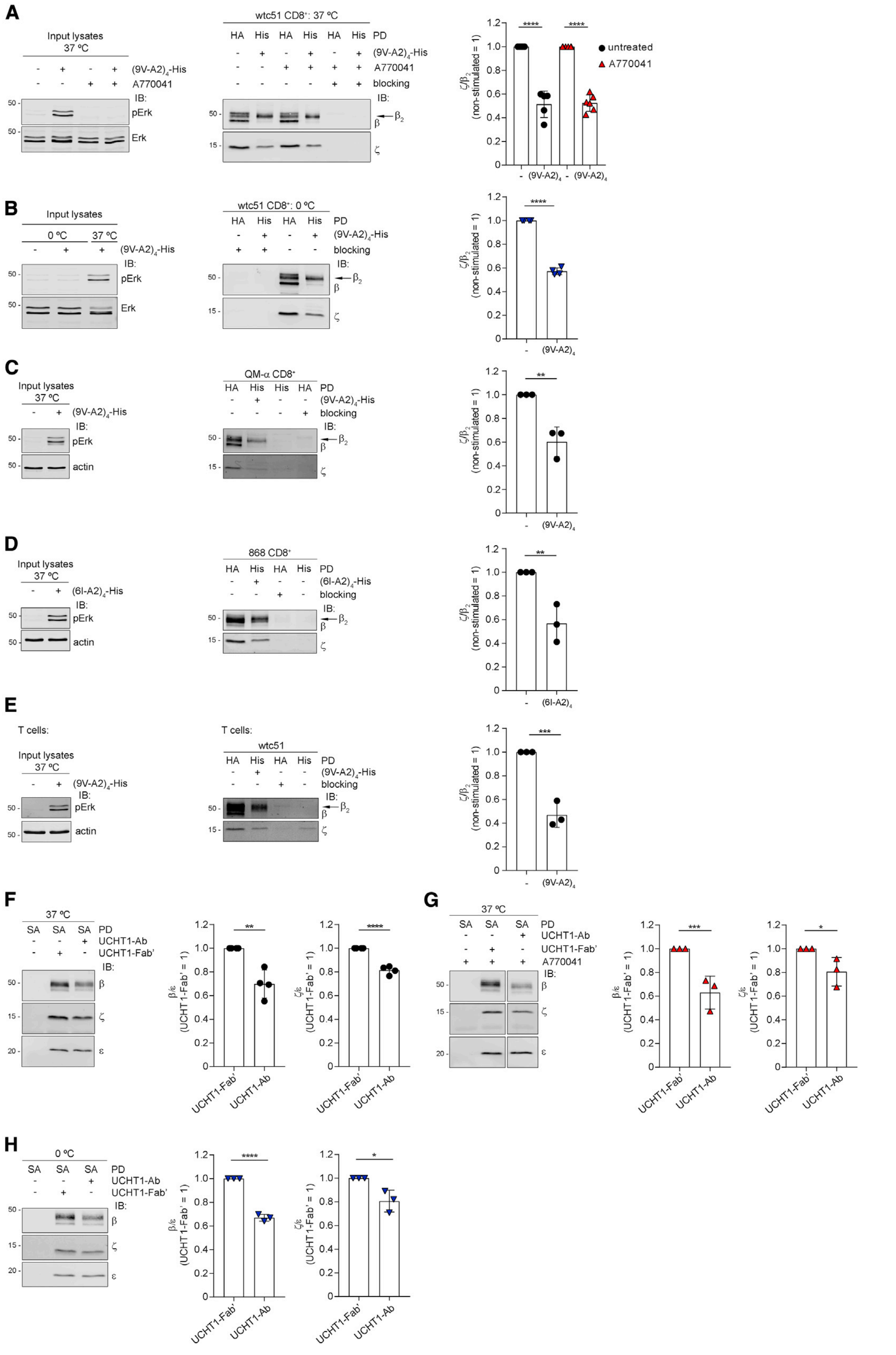

(legend on next page)

QM-α and 1G4-WT have superimposable canonical orientation when bound to NY-ESO-$1_{157\text{-}165}$-HLA-A2 (Figure S6B). Figure 6C showed that binding of $(9V\text{-}A2)_4$-His to QM-α induced strong Erk activation (Figure 6C, left panel) and reduced ζ/β_2 ratio (40%), which remained unchanged after A770041 treatment (Figure S6C). These observations were extended to 868, a TCR isolated from an HIV elite controller (Varela-Rohena et al., 2008). 868 recognizes the spontaneously mutated HIV p17 Gag-derived peptide SLYNTIATL (6I) presented by HLA-A2, with a K_d of 53 nM (k_{off}, 0.0013s^{-1} at 4°C; Cole et al., 2017). Being a natural TCR directed at a viral antigen, 868 was ideal to validate the data obtained with *in-vitro*-modified TCRs against a tumor antigen. Binding of tetramerized ligand $(6I\text{-}A2)_4$-His stimulated strong Erk activation (Figure 6D, left panel) and weakened 868 quaternary structure cohesion, as shown by the reduced ζ/β_2 ratio (Figure 6D, middle and right panels). The occurrence of the same effect (i.e., structural changes) in three different TCRs by mere coincidence is highly unlikely but rather the consequence of the same cause, namely, ligand-induced conformational changes that modify critical contacts maintaining TCR-CD3 complex cohesion (Alcover et al., 1990; Call et al., 2002; Dong et al., 2019). Reduced ζ cohesion was also observed in wtc51 when expressed in primary human T cells stimulated with $(9V\text{-}A2)_4$-His (Figure 6E), excluding non-physiological behavior of TCR-CD3 in the PM of Jurkat cells. To date, it remains unclear whether anti-CD3ε Abs used in clinical settings activate TCR-CD3 by mechanisms distinct from that of pMHC. To address this question, we slightly modified the DSA (STAR Methods). We used monobiotinylated Fab' of UCHT1 anti-CD3ε as a proxy for minimally or non-stimulated receptor and monobiotinylated UCHT1 Ab to stimulate and capture TCR-CD3 with streptavidin for IB analysis. Because TCR-CD3 was captured by CD3ε, β/ε and ζ/ε ratios were used to assess TCR-CD3 cohesion. UCHT1 Ab binding reduced β/ε and ζ/ε ratios and hence the cohesion of ε with β but less so with ζ (Figure 6F). Similar observations were made in cells pre-treated with A770041 (Figures 6G and S6D) or reacted with UCHT1 at 0°C (Figures 6H and S6E). Taken together, these observations and the TMR mutant phenotype strongly suggested that TCR-CD3 signals intracellularly by an allosteric interaction propagating from the $\alpha\beta$ binding site to the CD3 subunits and modifying critical contacts within the TMRs.

Soluble monovalent pMHC triggers TCR-CD3 untying and intracellular signaling

pMHC tetramers induced conformational change and signaling without force. However, pMHC tetramers necessarily induced fast TCR-CD3 clustering and therefore cannot allow to discern if receptor aggregation was responsible for allosteric activation, as previously suggested (Minguet et al., 2007). We therefore reacted wtc51-expressing J76 with biotinylated soluble monovalent (sm)-9V-A2 (Figure 7A). Monodispersion of (sm)-9V-A2 was controlled by size-exclusion chromatography-multi-angle-light scattering (SEC-MALS), just before use (Figure S7B, only fractions within the sm-9V-A2 peak were used). Following DDM solubilization, sm-9V-A2-bound TCR-CD3 was captured by His-streptavidin/His-Cobalt beads (Figure S7A) and ζ recovery examined. The ζ/β_2 ratio was considerably reduced in sm-9V-A2-bound versus unbound wtc51 and was unaffected by A770041 treatment (Figure 7A, IBs and histograms) or by the absence of the CD8 co-receptor (Figure 7B). To definitively exclude potential sm-pMHC cross-linking after solubilization by streptavidin used for capturing ligand-bound TCR-CD3, we used instead a monomeric Avidin (mAv). However, this condition did not change the result (Figure S7D). A similar ζ/β_2 reduction was observed for 868 TCR reacted with soluble monodispersed sm-6I-A2 (Figures 7C for DSA and Figure S7C for SEC). Figure 7E (IBs and histograms) shows that sm-9V-A2 reduced ζ recovery also in wtc51 expressed in primary T cells, excluding a bias of Jurkat cell PM. A more stringent test for TCR-CD3 allosteric regulation was to assess whether sm-pMHC promotes quaternary structure untying after solubilization. sm-9V-A2 bound to wtc51 TCR at 0°C in post-nuclear lysates with 0.5% DDM, as revealed by streptavidin IB (Figure S7E), considerably reduced the ζ/β_2 ratio (Figure 7F). Thus, TCR-CD3 loosening by pMHC binding did not require intact PM, and as it should be expected for an allosteric change, it relied essentially on protein-protein interactions. Moreover, because it occurred in isolated TCR-CD3, these data further corroborated the idea that the allosteric change was independent of force, clustering, and co-receptor. If the conformational change induced by sm-pMHC was functionally relevant, it should also induce intracellular signaling. Previous work could not demonstrate that binding of sm-pMHC in solution elicited a $[Ca^{2+}]_i$ increase unless the co-receptor was expressed

Figure 6. pMHC tetramer binding loosens $\alpha\beta$ association with ζ

(A) J76 wtc51 stimulated with $(9V\text{-}A2)_4$-His ± A770041. Left, representative pErk IB (n = 4). Middle, PD with anti-HA Ab (HA) (lanes 1, 3, 5) or His-Cobalt beads (His) (lanes 2, 4, 6) followed by IB for β and ζ. Blocking was with HA peptide or imidazole. The arrow indicates β_2 isoform. Right, x ± SD of ζ/β_2, n = 5–6, unpaired t test $p < 0.0001$.

(B) J76 wtc51 stimulated with $(9V\text{-}A2)_4$-His at 0°C. Left, representative pErk IB (n = 4). Middle, β-HA (lanes 1, 3) or His (lanes 2, 4) PD and IB for β and ζ. The arrow indicates β_2 isoform. Right, x ± SD of ζ/β_2, n = 4, unpaired t test $p < 0.0001$.

(C) J76 QM-α stimulated with $(9V\text{-}A2)_4$-His. Left, representative pErk IB (n = 3). Middle, β-HA (lanes 1, 4) or His (lanes 2, 3) PD and IB for β and ζ. The arrow indicates β_2 isoform. Right, x ± SD of ζ/β_2, n = 3, unpaired t test $p < 0.01$.

(D) J76 868 stimulated with $(6I\text{-}A2)_4$-His. Left, representative pErk IB (n = 3). Middle, β-HA (lanes 1, 3) or His (lanes 2, 4) PD and IB for β and ζ. The arrow indicates β_2 isoform. Right, x ± SD of ζ/β_2, n = 3, unpaired t test $p < 0.01$.

(E) Primary T cells expressing wtc51 stimulated with $(9V\text{-}A2)_4$-His. Left, representative pErk IB (n = 3). Middle, β-HA (lanes 1, 3) or His (lanes 2, 4) PD and IB for β and ζ. The arrow indicates β_2 isoform. Right, x ± SD of ζ/β_2, n = 3, unpaired t test $p < 0.001$.

(F) J76 1G4 ± UCHT1-Fab' or UCHT1-Ab. Left, streptavidin (SA)-mediated PD and IB for β, ζ, and ε. Right, x ± SD of β/ε and ζ/ε, n = 4, unpaired t test p = 0.0023, $p < 0.0001$.

(G) J76 1G4 ± A770041 incubated or not with UCHT1-Fab' or UCHT1-Ab. Left, SA-mediated PD and IB for β, ζ ,and ε.Right, x ± SD of β/ε and ζ/ε, n = 3, unpaired t test p = 0.0007, p = 0.0494. See also Figure S6D.

(H) J76 1G4 ± UCHT1-Fab' or UCHT1-Ab at 0°C. Left, SA-mediated PD and IB for β, ζ and ε. Right, x ± SD of β/ε and ζ/ε, n = 3, unpaired t test $p < 0.0001$, p = 0.0218. See also Figure S6E.

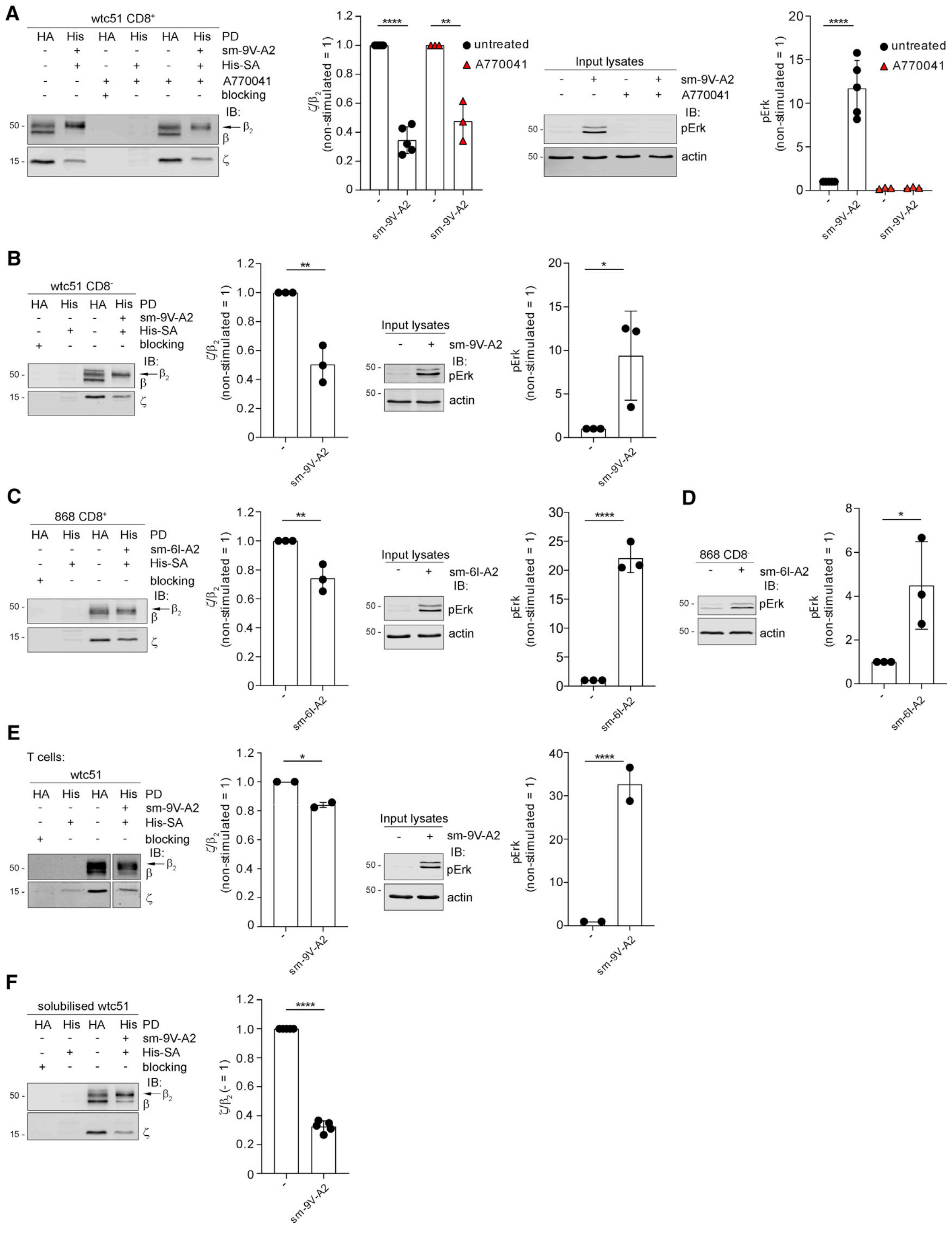

Figure 7. Monovalent pMHC in solution triggers TCR-CD3 untying and intracellular signaling

(A) J76 wtc51 ± A770041 stimulated or not with sm-9V-A2 were lysed and subjected to PD with anti-HA Ab or Talon beads. First panel, anti-HA (β-HA) (lanes 1, 3, 5) or Talon beads (His) (lanes 2, 4, 6) PD and IB for β and ζ. The arrow indicates β_2 isoform. Second panel, x ± SD of ζ/β_2, $n \geq 3$, unpaired t test $p < 0.0001$ and $p < 0.01$. Third panel, representative pErk IB. Fourth panel, x ± SD of pErk, $n \geq 3$, unpaired t test $p < 0.0001$.

(B) $CD8^-$ J76 wtc51 stimulated or not with sm-9V-A2 were processes as in (A). First panel, β-HA (lanes 1, 3) or His (lanes 2, 4) PD and IB for β and ζ. The arrow indicates β_2 isoform. Second panel, x ± SD of ζ/β_2, n = 3, unpaired t test $p < 0.01$. Third panel, representative pErk IB. Fourth panel, x ± SD of pErk, n = 3, unpaired t test $p < 0.05$.

(legend continued on next page)

(Delon et al., 1998). However, we found that sm-9V-A2, controlled by SEC-MALS for being monodispersed (Figure S7B), did induce robust pErk in both CD8-efficient (Figure 7A) and CD8-deficient (Figure 7B) J76 cells expressing wtc51 and erased by A770041 (Figure 7A). Erk activation by sm-9V-A2 was dose dependent (Figure S7F), with as little as 3 nM inducing 50% of the maximum and occurred at 2 min after sm-9V-A2 addition (Figure S7G), similar to $(9V\text{-}A2)_4$ stimulation of 1G4-WT (Paster et al., 2015), although $(9V\text{-}A2)_4$ generally induced a stronger pErk response. We obtained similar data with 868 in the presence or absence of a CD8 co-receptor (Figures 7C, 7D, and S7C) and with QM-α without CD8 (Figure S7H). Non-specific adsorption of sm-pMHC onto the J76 cell membrane during the stimulation assay was negligible even at the highest sm-9V-A2 concentration (Figure S7I). This made it unlikely that signaling was the consequence of sm-9V-A2 non-specific adsorption to the cell surface and ligand cross-presentation rather than direct stimulation by soluble sm-9V-A2. Moreover, we experimentally tested whether even this negligible amount of non-specifically bound sm-9V-A2 on J76 cells could be stimulatory. However, we did not detect any Erk activation (Figure S7J). Multiple reasons can explain why our data apparently conflict with previous observations. First and foremost, we used TCRs of reduced k_{off} (higher-affinity range) for pMHC, including a natural one (868). sm-pMHC ligands of low-medium affinity range (μM) can be expected to induce low/non-sustained $[Ca^{2+}]_i$ increase, whose ramp-up requires a complex cascade of additional events (Irvine et al., 2002; Lewis, 2020), including co-receptor implication (Delon et al., 1998; Minguet et al., 2007). Also, sm-pMHC engages TCR-CD3 without immediately clustering it, contrary to pMHC tetramers that provide this critical signaling-reinforcing effect (see Discussion). Moreover, membrane-tethered pMHC has lower degree of freedom than soluble pMHC, a property that sensibly increases pMHC on-rate (Huppa et al., 2010; O'Donoghue et al., 2013). Comprehensively, our genetic, biochemical, MDS, and functional data constitute substantial evidence that TCR-CD3 is a genuine allosteric device. We name this model "TCR-CD3 allosteric relaxation" (Figure S7K) as a mechanism sufficient to incite initial T cell activation solely by pMHC binding.

DISCUSSION

To gather insight into TCR-CD3 signaling, we used a genetic perturbation approach and found that TMR mutations that loosened cohesion between TCRαβ and CD3ζ increased the agonist's potency. This phenotype mimicked pMHC agonist binding that also reduced cohesion between TCRαβ and CD3ζ. These convergent results suggested that weakening of TCR-CD3 TMR contacts is a key step in an allosteric mechanism initiated by pMHC binding and culminating in ITAM phosphorylation. We favor the idea that conformational changes occurring upon pMHC binding propagate to CαCβ ECDs, where they contact the CD3 subunits (Beddoe et al., 2009; He et al., 2020; Natarajan et al., 2017; Rangarajan et al., 2018). The ECDs and TMRs of TCRαβ, CD3δε, and CD3γε show an extended intra-dimer interface (Dong et al., 2019). Moreover, CD3δε and CD3γε interact much more with TCRαβ than with each other. It is therefore conceivable that pMHC binding induces reshuffling of contacts between ECD dimers, loosening CD3δε and CD3γε from TCRαβ. This may result in slight rotation and/or translation of CD3δε and/or CD3γε vis-à-vis TCRαβ. The mechanical rigidity of ε, δ, and γ CPs (Alcover et al., 2018) could transmit these movements to the respective TMRs, resulting in local rearrangements of helix-helix packing and perhaps of interfacial lipids (Gupta et al., 2017; Figure S7K). Consistently, mutations of ε Cys-Cys loop affect TCR-CD3 signaling (Wang et al., 2009). Ligand-activated relaxed TCR-CD3 might reduce contacts between TMRs of αβ and ζζ, making ζζ prone to detaching from the rest of the complex by DDM during membrane extraction. As our data suggest, TMR quaternary structure relaxation activated by pMHC (or anti-CD3 Ab) or by TMR mutations promotes ITAM accessibility by active-Lck (Nika et al., 2010), which would require conformational changes of membrane-tethered CD3 intracellular tails (Xu et al., 2008). These are intrinsically disordered, and hence, they may lack mechanical rigidity required to respond to TMR movements. We suggest that subtle untying of TMRs may reduce the grip of CD3 intracellular tails to the membrane and favor ITAM tyrosine exposure (Figure S7K). Phosphatidylserine (PS) (Xu et al., 2008) and PIP2 (Chouaki Benmansour et al., 2018) appear to keep CD3 tails retracted onto the PM. A possibility is that TMR octamer rearrangement permits fast exchange of PIP2 and PS with neutral lipids that may reduce CD3ζ and ε tail interaction with the lipid bilayer (Figure S7K), augmenting ITAM tyrosine exposure to active-Lck. Changes in cholesterol interacting with TMR helices (Swamy et al., 2016; Wang et al., 2016) and/or ITAM tyrosines might be part of this mechanism. Agonist anti-CD3ε mAb produced a similar gain of function in TMR mutants, in agreement with CD3ε ECD lying on a conformational trajectory activated by pMHC.

We showed that binding of sm-pMHC to membrane-bound or detergent-solubilized TCR-CD3 suffices to induce TCR-CD3 relaxation and signal transduction. Stimulation of TCR-CD3 by sm-pMHC alone agrees with a genuine allosteric mechanism, as hinted by our genetic perturbation analysis. Allosteric activation occurred without co-receptor or clustering or force, making

(C) J76 868 stimulated or not with sm-6I-A2 were processed as in (A). First panel, β-HA (lanes 1, 3) or His (lanes 2, 4) PD and IB for β and ζ. The arrow indicates β_2 isoform. Second panel, x ± SD of ζ/β_2, n = 3, unpaired t test $p < 0.01$. Third panel, representative pErk IB. Fourth panel, x ± SD of pErk, n = 3, unpaired t test $p < 0.0001$.

(D) $CD8^-$ J76 868 stimulated or not with sm-6I-A2. Left, representative pErk IB. Right, x ± SD of pErk, n = 3, unpaired t test $p < 0.05$.

(E) Primary T cells expressing wtc51 stimulated or not with sm-9V-A2 were processed as in (A). First panel, β-HA (lanes 1, 3) or His (lanes 2, 4) PD and IB for β and ζ. The arrow indicates β_2 isoform. Second panel, x ± SEM of ζ/β_2, n = 2, unpaired t test $p < 0.05$. Third panel, representative pErk IB. Fourth panel, x ± SEM of pErk, n = 2, unpaired t test $p < 0.0001$.

(F) J76 wtc51 were lysed, incubated or not with sm-9V-A2 ,and subjected to PD by anti-HA or Talon beads. Left, β-HA (lanes 1, 3) or His (lanes 2, 4) PD and IB for β and ζ. The arrow indicates β_2 isoform. Right, x ± SD of ζ/β_2, n = 5, unpaired t test $p < 0.0001$.

actomyosin-induced membrane movements required for mechanosensing (Das et al., 2015; Kim et al., 2009; Liu et al., 2014) dispensable for signal ignition. A K_d of 7 μM could not allow to show quaternary structure relaxation of 9V-A2-bound versus free 1G4 TCR. However, this was possible with 1G4 variants wtc51 and QM-α capable of binding 9V-A2 with a K_d of 15 nM and 140 nM, which is within the physiological K_d range for pMHC agonists (Aleksic et al., 2012; Cole et al., 2007; Stone et al., 2009). A third example was 868, a cytotoxic T lymphocyte (CTL)-derived anti-HIV strong TCR pMHC binder. However, the DSA may limit the conclusions that can be drawn about weaker ligand interactions, and future work is required to address this conclusively. Nonetheless, allosterically regulated signaling by TCR-CD3 should be valid for the entire range of pMHC affinities, as independent studies showed that allosteric changes in TCRαβ occurred upon pMHC binding of K_d ranging between 5 and 0.04 μM (Beddoe et al., 2009; Natarajan et al., 2017; He et al., 2020; Rangarajan et al., 2018).

Allosterically induced interactions propagate at distances of nanometers in tens of μs to 1–2 ms (Kern and Zuiderweg, 2003; Natarajan et al., 2017; Rangarajan et al., 2018), which is much faster than the fastest half-lives (≈200 ms) of pMHC-TCRαβ interactions (e.g., positively selecting pMHC; Hong et al., 2018). Thus, it is conceivable that TCRαβ binding of very weak or strong agonists can activate TCR-CD3 by allosteric regulation. Allosterically regulated receptor tyrosine kinases (RTKs) and G-protein coupled receptors (GPCRs) function with allosteric modality when binding ligands of a wide difference in affinity (Freed et al., 2017; Furness et al., 2016; Lane et al., 2017).

Early work did not detect a $[Ca^{2+}]_i$ increase by the pMHC monomer (Boniface et al., 1998), unless CD8 was co-engaged (Delon et al., 1998). This apparent conflict with our data can be reconciled by considering differences in sensitivity of the outputs measured (i.e., $[Ca^{2+}]_i$ versus pErk) and increased pMHC dwell-time binding in our experiments. Moreover, membrane-tethered pMHCs have considerably increased k_{on} (and little or no change in k_{off}) compared to soluble pMHC (Huppa et al., 2010; O'Donoghue et al., 2013), indicating more effective entropically -driven signaling by the former. Moreover, $[Ca^{2+}]_i$ high amplitude and duration require sustained TCRαβ engagement (Irvine et al., 2002; Lewis, 2020) achieved by higher lateral ordering of signaling complexes in micro-clusters at the immunological synapse (Varma et al., 2006). Co-receptors and clustering allow recording robust intracellular signaling required for full T cell activation, which may not be required for just igniting TCR-CD3 signal transduction as sm-pMHC alone does.

Reduced k_{off} of the TCR-pMHC interaction is observed when subjected to ≈10–20 pN pulling force, meaning "catch-bond" formation (Liu et al., 2014). However, mechanical force generated by membrane fluctuations and/or dedicated actin protrusions occur on a timescale of several seconds, which is two to three orders of magnitude slower than allosteric changes. Thus, pMHC-induced initial signal transduction may occur without mechanical force. Interestingly, recent biophysical data suggest that force developed between membrane-tethered TCR-CD3 and pMHC is of low magnitude (≈ 2pN) (Göhring et al., 2021). Perhaps, low-amplitude force may extend purely allosterically induced interactions.

Changes in conformational dynamics have long-range consequences of functional relevance, a mechanism known as dynamic allostery relying on conformational entropy (Tzeng and Kalodimos, 2012). Conformational entropy cannot be frequently observed in proteins' crystalline state, as it is unlikely to capture a higher-energy (activated) state. However, NMR can correlate fast local conformational changes (ps, ns) occurring at distant sites over timescales of μs to ms and distances of nm (Kern and Zuiderweg, 2003; Natarajan et al., 2017). Dynamic allostery may apply to TCRαβ, as pMHC binding induces changes in conformational dynamics at distal H3, H4 helices, and FG loop of Cβ and Cα AB loop (Beddoe et al., 2009; He et al., 2020; Natarajan et al., 2017; Rangarajan et al., 2018), with the latter having been captured thus far in only a single crystal structure (Kjer-Nielsen et al., 2003).

Our data should help reconcile controversies about the TCR-CD3 signaling mechanism. Thus, pMHC co-engagement by TCR and co-receptor is conditional on initial TCR-CD3 signaling (Casas et al., 2014; Jiang et al., 2011), and catch-bonding appears to depend in part on TCR signaling (Hong et al., 2018). We found evidence for enhanced basal signaling inducing clustering that is erased by Lck inhibition. These data suggest that all these events are instigated by an initial mechanism of allosteric nature induced solely by pMHC binding. Thus, co-receptor engagement, receptor clustering, shielding from PTPs, and actomyosin-driven mechanical force may stabilize/potentiate initial allosterically induced signaling by helping reduce physical and chemical noise and augmenting/stabilizing signals of narrow amplitude and duration initiated by sparse engagement of pMHC (Brameshuber et al., 2018).

Binding of different CDR interfaces might activate different conformational changes. If so, ligand potency may reflect combinations of binding kinetics and conformational trajectories. Alternatively, different ligand-receptor pairs may induce similar conformational trajectories. Future functional studies with TCR-CD3 binding pMHC with non-noncanonical orientations (Adams et al., 2011), MDSs, native nanodiscs, and genetic perturbation should help clarify this question and provide further insights on the mechanism that triggers TCR-CD3 signaling.

Limitations of the study

We have shown that signaling is activated by pMHC-induced changes of TCR-CD3 quaternary structure. For this study, we had to use TCRs of reduced k_{off} for pMHC, as limitations of DSA did not allow us to show the same effect for weak agonists. However, structural studies have shown allosteric changes occurring upon CαCβ binding to medium-range agonists. Moreover, conformational change propagation occurs at two orders of magnitude faster than the dwell-time of the weakest agonists, such as that of self-pMHC. Although this finding supports our conclusions, further work is required to formally demonstrate their validity regardless of TCR affinity for pMHC.

STAR★METHODS

Detailed methods are provided in the online version of this paper and include the following:

- KEY RESOURCES TABLE

- RESOURCE AVAILABILITY
  - Lead contact
  - Materials availability
  - Data and code availability
- EXPERIMENTAL MODEL AND SUBJECT DETAILS
  - Cell lines
  - Primary T cells
- METHOD DETAILS
  - DNA constructs and cloning
  - CRISPR/Cas9-mediated ζ knock-out
  - TCR-CD3 expression
  - Preparation of pMHC monomers and tetramers
  - Preparation of UCHT1-Fab' fragment
  - Flow cytometry
  - Mathematical modeling
  - Biochemical analysis of the TCR-CD3 complex
  - Ligand-induced TCR-CD3 quaternary structure changes
  - Microscopy
  - Atomistic Molecular Dynamics Simulations
  - Modeling of the 1G4 TCR affinity mutants
- QUANTIFICATION AND STATISTICAL ANALYSIS
  - Flow cytometry
  - Pull-down and Immunoblotting
  - Microscopy
  - Simulations
  - Modeling
  - Significance

SUPPLEMENTAL INFORMATION

Supplemental information can be found online at https://doi.org/10.1016/j.celrep.2021.109375.

ACKNOWLEDGMENTS

We acknowledge the following: grant WT200844/Z/16/Z to O.A., WT092970MA and WT208361/Z/17/Z to M.S.P.S., WT100262Z/12/Z to M.L.D., WT207537/Z/17/Z to O.D., WTSBF002\1031, Springboard Award from the Academy of Medical Sciences (GB) to A.C.K, ERC-2014-AdG 670930 to S.B., Care-for-Rare Postgraduate Fellowship to A.-L.L., and High-Performance Computing facilities, University of Leeds. We thank Juliane Cohen, Christine Ralf, and Salvatore Valvo for technical support; John Orban, Brian Baker, Ian Wilson, Gerhard Schütz, Pedro Carvalho, Marco Fritzsche, Hai-Tao He, Immanuel Luescher, Cheng Zhu, and Andrew Sewell for discussions, suggestions, and reading the manuscript; and C.R. and J.C. and Ana Maria Vallés for manuscript editing. We apologize to colleagues whose work could not be adequately cited here because of space limitation.

AUTHOR CONTRIBUTIONS

Conceptualization, O.A.; methodology, A.-L.L., G.M., N.P., A.C., D.C., R.R., D.K.C., M.L., S.B., A.C.K., D.P., O.D., M.L.D., M.S.P.S., and O.A.; software, A.C.K. and O.D.; formal data analysis, A.-L.L., G.M., N.P., A.C., D.C., D.P., S.B., O.D., M.S.P.S., and O.A.; investigation, A.-L.L., G.M., N.P., A.C., D.C., A.C.K., S.B., D.G., D.K.C., D.P., M.S.P.S., and O.A.; resources, S.B.; writing – original draft, O.A.; writing – review and editing, O.A., A.-L.L., G.M., N.P., A.C., D.P., A.C.K., M.S.P.S., D.K.C., M.L., M.L.D., and D.C.; visualization, G.M., A.-L.L., N.P., A.C.K., A.C., D.C., D.P., D.K.C., S.B., and O.A.; supervision, A.C.K., M.S.P.S., M.L.D., and O.A.; funding acquisition, A.C.K., M.S.P.S., M.L.D., and O.A.

DECLARATION OF INTERESTS

The authors declare no competing interests.

Received: October 7, 2020
Revised: May 5, 2021
Accepted: June 18, 2021
Published: July 13, 2021

REFERENCES

Acuto, O., Di Bartolo, V., and Michel, F. (2008). Tailoring T-cell receptor signals by proximal negative feedback mechanisms. Nat. Rev. Immunol. *8*, 699–712.

Adams, J.J., Narayanan, S., Liu, B., Birnbaum, M.E., Kruse, A.C., Bowerman, N.A., Chen, W., Levin, A.M., Connolly, J.M., Zhu, C., et al. (2011). T cell receptor signaling is limited by docking geometry to peptide-major histocompatibility complex. Immunity *35*, 681–693.

Alcover, A., Mariuzza, R.A., Ermonval, M., and Acuto, O. (1990). Lysine 271 in the transmembrane domain of the T-cell antigen receptor beta chain is necessary for its assembly with the CD3 complex but not for alpha/beta dimerization. J. Biol. Chem. *265*, 4131–4135.

Alcover, A., Alarcón, B., and Di Bartolo, V. (2018). Cell Biology of T Cell Receptor Expression and Regulation. Annu. Rev. Immunol. *36*, 103–125.

Aleksic, M., Dushek, O., Zhang, H., Shenderov, E., Chen, J.L., Cerundolo, V., Coombs, D., and van der Merwe, P.A. (2010). Dependence of T cell antigen recognition on T cell receptor-peptide MHC confinement time. Immunity *32*, 163–174.

Aleksic, M., Liddy, N., Molloy, P.E., Pumphrey, N., Vuidepot, A., Chang, K.M., and Jakobsen, B.K. (2012). Different affinity windows for virus and cancer-specific T-cell receptors: implications for therapeutic strategies. Eur. J. Immunol. *42*, 3174–3179.

Altman, J.D., and Davis, M.M. (2003). MHC-peptide tetramers to visualize antigen-specific T cells. Curr. Protoc. Immunol. *Chapter 17*, Unit 17.3.

Baker, B.M., Scott, D.R., Blevins, S.J., and Hawse, W.F. (2012). Structural and dynamic control of T-cell receptor specificity, cross-reactivity, and binding mechanism. Immunol. Rev. *250*, 10–31.

Bates, M., Huang, B., Dempsey, G.T., and Zhuang, X. (2007). Multicolor super-resolution imaging with photo-switchable fluorescent probes. Science *317*, 1749–1753.

Beddoe, T., Chen, Z., Clements, C.S., Ely, L.K., Bushell, S.R., Vivian, J.P., Kjer-Nielsen, L., Pang, S.S., Dunstone, M.A., Liu, Y.C., et al. (2009). Antigen ligation triggers a conformational change within the constant domain of the alphabeta T cell receptor. Immunity *30*, 777–788.

Boniface, J.J., Rabinowitz, J.D., Wülfing, C., Hampl, J., Reich, Z., Altman, J.D., Kantor, R.M., Beeson, C., McConnell, H.M., and Davis, M.M. (1998). Initiation of signal transduction through the T cell receptor requires the multivalent engagement of peptide/MHC ligands [corrected]. Immunity *9*, 459–466.

Brameshuber, M., Kellner, F., Rossboth, B.K., Ta, H., Alge, K., Sevcsik, E., Göhring, J., Axmann, M., Baumgart, F., Gascoigne, N.R.J., et al. (2018). Monomeric TCRs drive T cell antigen recognition. Nat. Immunol. *19*, 487–496.

Call, M.E., Pyrdol, J., Wiedmann, M., and Wucherpfennig, K.W. (2002). The organizing principle in the formation of the T cell receptor-CD3 complex. Cell *111*, 967–979.

Casas, J., Brzostek, J., Zarnitsyna, V.I., Hong, J.S., Wei, Q., Hoerter, J.A., Fu, G., Ampudia, J., Zamoyska, R., Zhu, C., and Gascoigne, N.R. (2014). Ligand-engaged TCR is triggered by Lck not associated with CD8 coreceptor. Nat. Commun. *5*, 5624.

Changeux, J.P., and Christopoulos, A. (2016). Allosteric Modulation as a Unifying Mechanism for Receptor Function and Regulation. Cell *166*, 1084–1102.

Chen, J.L., Stewart-Jones, G., Bossi, G., Lissin, N.M., Wooldridge, L., Choi, E.M., Held, G., Dunbar, P.R., Esnouf, R.M., Sami, M., et al. (2005). Structural and kinetic basis for heightened immunogenicity of T cell vaccines. J. Exp. Med. *201*, 1243–1255.

CellPress
OPEN ACCESS

Chouaki Benmansour, N., Ruminski, K., Sartre, A.M., Phelipot, M.C., Salles, A., Bergot, E., Wu, A., Chicanne, G., Fallet, M., Brustlein, S., et al. (2018). Phosphoinositides regulate the TCR/CD3 complex membrane dynamics and activation. Sci. Rep. *8*, 4966.

Circosta, P., Granziero, L., Follenzi, A., Vigna, E., Stella, S., Vallario, A., Elia, A.R., Gammaitoni, L., Vitaggio, K., Orso, F., et al. (2009). T cell receptor (TCR) gene transfer with lentiviral vectors allows efficient redirection of tumor specificity in naive and memory T cells without prior stimulation of endogenous TCR. Hum. Gene Ther. *20*, 1576–1588.

Cochran, J.R., Aivazian, D., Cameron, T.O., and Stern, L.J. (2001). Receptor clustering and transmembrane signaling in T cells. Trends Biochem. Sci. *26*, 304–310.

Cole, D.K., Pumphrey, N.J., Boulter, J.M., Sami, M., Bell, J.I., Gostick, E., Price, D.A., Gao, G.F., Sewell, A.K., and Jakobsen, B.K. (2007). Human TCR-binding affinity is governed by MHC class restriction. J. Immunol. *178*, 5727–5734.

Cole, D.K., Fuller, A., Dolton, G., Zervoudi, E., Legut, M., Miles, K., Blanchfield, L., Madura, F., Holland, C.J., Bulek, A.M., et al. (2017). Dual Molecular Mechanisms Govern Escape at Immunodominant HLA A2-Restricted HIV Epitope. Front. Immunol. *8*, 1503.

Das, D.K., Feng, Y., Mallis, R.J., Li, X., Keskin, D.B., Hussey, R.E., Brady, S.K., Wang, J.H., Wagner, G., Reinherz, E.L., and Lang, M.J. (2015). Force-dependent transition in the T-cell receptor β-subunit allosterically regulates peptide discrimination and pMHC bond lifetime. Proc. Natl. Acad. Sci. USA *112*, 1517–1522.

Davis, S.J., and van der Merwe, P.A. (2006). The kinetic-segregation model: TCR triggering and beyond. Nat. Immunol. *7*, 803–809.

Davis, M.M., Krogsgaard, M., Huse, M., Huppa, J., Lillemeier, B.F., and Li, Q.J. (2007). T cells as a self-referential, sensory organ. Annu. Rev. Immunol. *25*, 681–695.

Deford-Watts, L.M., Tassin, T.C., Becker, A.M., Medeiros, J.J., Albanesi, J.P., Love, P.E., Wülfing, C., and van Oers, N.S. (2009). The cytoplasmic tail of the T cell receptor CD3 epsilon subunit contains a phospholipid-binding motif that regulates T cell functions. J. Immunol. *183*, 1055–1064.

DeLano, W.L. (2002). The PyMOL Molecular Graphics System. Version 1 (Schrödinger LLC).

Delon, J., Grégoire, C., Malissen, B., Darche, S., Lemaître, F., Kourilsky, P., Abastado, J.P., and Trautmann, A. (1998). CD8 expression allows T cell signaling by monomeric peptide-MHC complexes. Immunity *9*, 467–473.

Dong, D., Zheng, L., Lin, J., Zhang, B., Zhu, Y., Li, N., Xie, S., Wang, Y., Gao, N., and Huang, Z. (2019). Structural basis of assembly of the human T cell receptor-CD3 complex. Nature *573*, 546–552.

Dushek, O., Aleksic, M., Wheeler, R.J., Zhang, H., Cordoba, S.P., Peng, Y.C., Chen, J.L., Cerundolo, V., Dong, T., Coombs, D., and van der Merwe, P.A. (2011). Antigen potency and maximal efficacy reveal a mechanism of efficient T cell activation. Sci. Signal. *4*, ra39.

Emsley, P., and Cowtan, K. (2004). Coot: model-building tools for molecular graphics. Acta Crystallogr. D Biol. Crystallogr. *60*, 2126–2132.

Essmann, U., Perera, L., Berkowitz, M.L., Darden, T., Lee, H., and Pedersen, L.G. (1995). A smooth particle mesh Ewald method. J. Chem. Phys. *103*, 8577–8593.

Ester, M., Kriegel, H.-P., Sander, J., and Xu, X. (1996). A Density-Based Algorithm for Discovering Clusters in Large Spatial Databases with Noise. In KDD-96 Proceedings of the Second International Conference on Knowledge Discovery and Data Mining (AAAI Press), pp. 226–231.

Feige, M.J., and Hendershot, L.M. (2013). Quality control of integral membrane proteins by assembly-dependent membrane integration. Mol. Cell *51*, 297–309.

Freed, D.M., Bessman, N.J., Kiyatkin, A., Salazar-Cavazos, E., Byrne, P.O., Moore, J.O., Valley, C.C., Ferguson, K.M., Leahy, D.J., Lidke, D.S., and Lemmon, M.A. (2017). EGFR Ligands Differentially Stabilize Receptor Dimers to Specify Signaling Kinetics. Cell *171*, 683–695.e18.

Furness, S.G.B., Liang, Y.L., Nowell, C.J., Halls, M.L., Wookey, P.J., Dal Maso, E., Inoue, A., Christopoulos, A., Wootten, D., and Sexton, P.M. (2016). Ligand-Dependent Modulation of G Protein Conformation Alters Drug Efficacy. Cell *167*, 739–749.e11.

Garboczi, D.N., Ghosh, P., Utz, U., Fan, Q.R., Biddison, W.E., and Wiley, D.C. (1996). Structure of the complex between human T-cell receptor, viral peptide and HLA-A2. Nature *384*, 134–141.

Garcia, K.C., Degano, M., Stanfield, R.L., Brunmark, A., Jackson, M.R., Peterson, P.A., Teyton, L., and Wilson, I.A. (1996). An alphabeta T cell receptor structure at 2.5 A and its orientation in the TCR-MHC complex. Science *274*, 209–219.

Garcia, K.C., Gapin, L., Adams, J.J., Birnbaum, M.E., Scott-Browne, J.P., Kappler, J.W., and Marrack, P. (2012). A closer look at TCR germline recognition. Immunity *36*, 887–888.

Gil, D., Schamel, W.W., Montoya, M., Sánchez-Madrid, F., and Alarcón, B. (2002). Recruitment of Nck by CD3 epsilon reveals a ligand-induced conformational change essential for T cell receptor signaling and synapse formation. Cell *109*, 901–912.

Göhring, J., Kellner, F., Schrangl, L., Platzer, R., Klotzsch, E., Stockinger, H., Huppa, J.B., and Schütz, G.J. (2021). Temporal analysis of T-cell receptor-imposed forces via quantitative single molecule FRET measurements. Nat. Commun. *12*, 2502.

Gupta, K., Donlan, J.A.C., Hopper, J.T.S., Uzdavinys, P., Landreh, M., Struwe, W.B., Drew, D., Baldwin, A.J., Stansfeld, P.J., and Robinson, C.V. (2017). The role of interfacial lipids in stabilizing membrane protein oligomers. Nature *541*, 421–424.

Hawse, W.F., Champion, M.M., Joyce, M.V., Hellman, L.M., Hossain, M., Ryan, V., Pierce, B.G., Weng, Z., and Baker, B.M. (2012). Cutting edge: Evidence for a dynamically driven T cell signaling mechanism. J. Immunol. *188*, 5819–5823.

He, Y., Rangarajan, S., Kerzic, M., Luo, M., Chen, Y., Wang, Q., Yin, Y., Workman, C.J., Vignali, K.M., Vignali, D.A., et al. (2015). Identification of the Docking Site for CD3 on the T Cell Receptor β Chain by Solution NMR. J. Biol. Chem. *290*, 19796–19805.

He, Y., Agnihotri, P., Rangarajan, S., Chen, Y., Kerzic, M.C., Ma, B., Nussinov, R., Mariuzza, R.A., and Orban, J. (2020). Peptide-MHC Binding Reveals Conserved Allosteric Sites in MHC Class I- and Class II-Restricted T Cell Receptors (TCRs). J. Mol. Biol. *432*, 166697.

Heemskerk, M.H., Hoogeboom, M., de Paus, R.A., Kester, M.G., van der Hoorn, M.A., Goulmy, E., Willemze, R., and Falkenburg, J.H. (2003). Redirection of antileukemic reactivity of peripheral T lymphocytes using gene transfer of minor histocompatibility antigen HA-2-specific T-cell receptor complexes expressing a conserved alpha joining region. Blood *102*, 3530–3540.

Hess, B., Bekker, H., Berendsen, H.J.C., and Fraaije, J.G.E.M. (1997). LINCS: a linear constraint solver for molecular simulations. J. Comput. Chem. *18*, 1463–1472.

Hong, J., Ge, C., Jothikumar, P., Yuan, Z., Liu, B., Bai, K., Li, K., Rittase, W., Shinzawa, M., Zhang, Y., et al. (2018). A TCR mechanotransduction signaling loop induces negative selection in the thymus. Nat. Immunol. *19*, 1379–1390.

Hoover, W.G. (1985). Canonical dynamics: Equilibrium phase-space distributions. Phys. Rev. A Gen. Physiol. *31*, 1695–1697.

Huang, B., Wang, W., Bates, M., and Zhuang, X. (2008). Three-dimensional super-resolution imaging by stochastic optical reconstruction microscopy. Science *319*, 810–813.

Humphrey, W., Dalke, A., and Schulten, K. (1996). VMD: visual molecular dynamics. J. Mol. Graph *14*, 33–38, 27–38.

Huppa, J.B., Axmann, M., Mörtelmaier, M.A., Lillemeier, B.F., Newell, E.W., Brameshuber, M., Klein, L.O., Schütz, G.J., and Davis, M.M. (2010). TCR-peptide-MHC interactions in situ show accelerated kinetics and increased affinity. Nature *463*, 963–967.

Huse, M., Klein, L.O., Girvin, A.T., Faraj, J.M., Li, Q.J., Kuhns, M.S., and Davis, M.M. (2007). Spatial and temporal dynamics of T cell receptor signaling with a photoactivatable agonist. Immunity *27*, 76–88.

Irvine, D.J., Purbhoo, M.A., Krogsgaard, M., and Davis, M.M. (2002). Direct observation of ligand recognition by T cells. Nature *419*, 845–849.

Irving, M., Zoete, V., Hebeisen, M., Schmid, D., Baumgartner, P., Guillaume, P., Romero, P., Speiser, D., Luescher, I., Rufer, N., and Michielin, O. (2012). Interplay between T cell receptor binding kinetics and the level of cognate peptide presented by major histocompatibility complexes governs CD8+ T cell responsiveness. J. Biol. Chem. *287*, 23068–23078.

Jiang, N., Huang, J., Edwards, L.J., Liu, B., Zhang, Y., Beal, C.D., Evavold, B.D., and Zhu, C. (2011). Two-stage cooperative T cell receptor-peptide major histocompatibility complex-CD8 trimolecular interactions amplify antigen discrimination. Immunity *34*, 13–23.

Kern, D., and Zuiderweg, E.R. (2003). The role of dynamics in allosteric regulation. Curr. Opin. Struct. Biol. *13*, 748–757.

Kim, S.T., Takeuchi, K., Sun, Z.Y., Touma, M., Castro, C.E., Fahmy, A., Lang, M.J., Wagner, G., and Reinherz, E.L. (2009). The alphabeta T cell receptor is an anisotropic mechanosensor. J. Biol. Chem. *284*, 31028–31037.

Kjer-Nielsen, L., Clements, C.S., Purcell, A.W., Brooks, A.G., Whisstock, J.C., Burrows, S.R., McCluskey, J., and Rossjohn, J. (2003). A structural basis for the selection of dominant alphabeta T cell receptors in antiviral immunity. Immunity *18*, 53–64.

Lane, J.R., May, L.T., Parton, R.G., Sexton, P.M., and Christopoulos, A. (2017). A kinetic view of GPCR allostery and biased agonism. Nat. Chem. Biol. *13*, 929–937.

Lee, J., Cheng, X., Swails, J.M., Yeom, M.S., Eastman, P.K., Lemkul, J.A., Wei, S., Buckner, J., Jeong, J.C., Qi, Y., et al. (2016). CHARMM-GUI Input Generator for NAMD, GROMACS, AMBER, OpenMM, and CHARMM/OpenMM Simulations Using the CHARMM36 Additive Force Field. J. Chem. Theory Comput. *12*, 405–413.

Lewis, R.S. (2020). Store-Operated Calcium Channels: From Function to Structure and Back Again. Cold Spring Harb. Perspect. Biol. *12*, a035055.

Liu, B., Chen, W., Evavold, B.D., and Zhu, C. (2014). Accumulation of dynamic catch bonds between TCR and agonist peptide-MHC triggers T cell signaling. Cell *157*, 357–368.

Mariuzza, R.A., Agnihotri, P., and Orban, J. (2020). The structural basis of T-cell receptor (TCR) activation: An enduring enigma. J. Biol. Chem. *295*, 914–925.

Marrack, P., Scott-Browne, J.P., Dai, S., Gapin, L., and Kappler, J.W. (2008). Evolutionarily conserved amino acids that control TCR-MHC interaction. Annu. Rev. Immunol. *26*, 171–203.

McKeithan, T.W. (1995). Kinetic proofreading in T-cell receptor signal transduction. Proc. Natl. Acad. Sci. USA *92*, 5042–5046.

Minguet, S., Swamy, M., Alarcón, B., Luescher, I.F., and Schamel, W.W. (2007). Full activation of the T cell receptor requires both clustering and conformational changes at CD3. Immunity *26*, 43–54.

Natarajan, A., Nadarajah, V., Felsovalyi, K., Wang, W., Jeyachandran, V.R., Wasson, R.A., Cardozo, T., Bracken, C., and Krogsgaard, M. (2016). Structural Model of the Extracellular Assembly of the TCR-CD3 Complex. Cell Rep. *14*, 2833–2845.

Natarajan, K., McShan, A.C., Jiang, J., Kumirov, V.K., Wang, R., Zhao, H., Schuck, P., Tilahun, M.E., Boyd, L.F., Ying, J., et al. (2017). An allosteric site in the T-cell receptor Cβ domain plays a critical signalling role. Nat. Commun. *8*, 15260.

Nika, K., Soldani, C., Salek, M., Paster, W., Gray, A., Etzensperger, R., Fugger, L., Polzella, P., Cerundolo, V., Dushek, O., et al. (2010). Constitutively active Lck kinase in T cells drives antigen receptor signal transduction. Immunity *32*, 766–777.

O'Donoghue, G.P., Pielak, R.M., Smoligovets, A.A., Lin, J.J., and Groves, J.T. (2013). Direct single molecule measurement of TCR triggering by agonist pMHC in living primary T cells. eLife *2*, e00778.

Pageon, S.V., Nicovich, P.R., Mollazade, M., Tabarin, T., and Gaus, K. (2016). Clus-DoC: a combined cluster detection and colocalization analysis for single-molecule localization microscopy data. Mol. Biol. Cell *27*, 3627–3636.

Parrinello, M., and Rahman, A. (1981). T Polymorphic transitions in single crystals: A new molecular dynamics method. J. Appl. Phys. *52*, 7182–7190.

Paster, W., Bruger, A.M., Katsch, K., Grégoire, C., Roncagalli, R., Fu, G., Gascoigne, N.R., Nika, K., Cohnen, A., Feller, S.M., et al. (2015). A THEMIS:SHP1 complex promotes T-cell survival. EMBO J. *34*, 393–409.

Rangarajan, S., He, Y., Chen, Y., Kerzic, M.C., Ma, B., Gowthaman, R., Pierce, B.G., Nussinov, R., Mariuzza, R.A., and Orban, J. (2018). Peptide-MHC (pMHC) binding to a human antiviral T cell receptor induces long-range allosteric communication between pMHC- and CD3-binding sites. J. Biol. Chem. *293*, 15991–16005.

Ryan, M.D., King, A.M., and Thomas, G.P. (1991). Cleavage of foot-and-mouth disease virus polyprotein is mediated by residues located within a 19 amino acid sequence. J. Gen. Virol. *72*, 2727–2732.

San José, E., Sahuquillo, A.G., Bragado, R., and Alarcón, B. (1998). Assembly of the TCR/CD3 complex: CD3 epsilon/delta and CD3 epsilon/gamma dimers associate indistinctly with both TCR alpha and TCR beta chains. Evidence for a double TCR heterodimer model. Eur. J. Immunol. *28*, 12–21.

Shelby, S.A., Holowka, D., Baird, B., and Veatch, S.L. (2013). Distinct stages of stimulated FcεRI receptor clustering and immobilization are identified through superresolution imaging. Biophys. J. *105*, 2343–2354.

Stachlewitz, R.F., Hart, M.A., Bettencourt, B., Kebede, T., Schwartz, A., Ratnofsky, S.E., Calderwood, D.J., Waegell, W.O., and Hirst, G.C. (2005). A-770041, a novel and selective small-molecule inhibitor of Lck, prevents heart allograft rejection. J. Pharmacol. Exp. Ther. *315*, 36–41.

Stone, J.D., Chervin, A.S., and Kranz, D.M. (2009). T-cell receptor binding affinities and kinetics: impact on T-cell activity and specificity. Immunology *126*, 165–176.

Stone, M.B., Shelby, S.A., Núñez, M.F., Wisser, K., and Veatch, S.L. (2017). Protein sorting by lipid phase-like domains supports emergent signaling function in B lymphocyte plasma membranes. eLife *6*, e19891.

Stritesky, G.L., Jameson, S.C., and Hogquist, K.A. (2012). Selection of self-reactive T cells in the thymus. Annu. Rev. Immunol. *30*, 95–114.

Swamy, M., Dopfer, E.P., Molnar, E., Alarcón, B., and Schamel, W.W. (2008). The 450 kDa TCR Complex has a Stoichiometry of alphabetagammaepsilon-deltaepsilonzetazeta. Scand. J. Immunol. *67*, 418–420, author reply 421.

Swamy, M., Beck-Garcia, K., Beck-Garcia, E., Hartl, F.A., Morath, A., Yousefi, O.S., Dopfer, E.P., Molnár, E., Schulze, A.K., Blanco, R., et al. (2016). A Cholesterol-Based Allostery Model of T Cell Receptor Phosphorylation. Immunity *44*, 1091–1101.

Testi, R., Alcover, A., Spagnoli, G., Reinherz, E.L., and Acuto, O. (1989). CD3Ti+ human thymocyte-derived clones displaying a differential response to activation via CD3Ti and CD2. Cell. Immunol. *122*, 350–364.

Tischer, D.K., and Weiner, O.D. (2019). Light-based tuning of ligand half-life supports kinetic proofreading model of T cell signaling. eLife *8*, e42498.

Tzeng, S.R., and Kalodimos, C.G. (2012). Protein activity regulation by conformational entropy. Nature *488*, 236–240.

Van Der Spoel, D., Lindahl, E., Hess, B., Groenhof, G., Mark, A.E., and Berendsen, H.J. (2005). GROMACS: fast, flexible, and free. J. Comput. Chem. *26*, 1701–1718.

Varela-Rohena, A., Molloy, P.E., Dunn, S.M., Li, Y., Suhoski, M.M., Carroll, R.G., Milicic, A., Mahon, T., Sutton, D.H., Laugel, B., et al. (2008). Control of HIV-1 immune escape by CD8 T cells expressing enhanced T-cell receptor. Nat. Med. *14*, 1390–1395.

Varma, R., Campi, G., Yokosuka, T., Saito, T., and Dustin, M.L. (2006). T cell receptor-proximal signals are sustained in peripheral microclusters and terminated in the central supramolecular activation cluster. Immunity *25*, 117–127.

Veatch, S.L., Machta, B.B., Shelby, S.A., Chiang, E.N., Holowka, D.A., and Baird, B.A. (2012). Correlation functions quantify super-resolution images and estimate apparent clustering due to over-counting. PLoS ONE *7*, e31457.

Volkman, B.F., Lipson, D., Wemmer, D.E., and Kern, D. (2001). Two-state allosteric behavior in a single-domain signaling protein. Science *291*, 2429–2433.

Wang, Y., Becker, D., Vass, T., White, J., Marrack, P., and Kappler, J.W. (2009). A conserved CXXC motif in CD3epsilon is critical for T cell development and TCR signaling. PLoS Biol. *7*, e1000253.

CellPress
OPEN ACCESS

Wang, F., Beck-García, K., Zorzin, C., Schamel, W.W., and Davis, M.M. (2016). Inhibition of T cell receptor signaling by cholesterol sulfate, a naturally occurring derivative of membrane cholesterol. Nat. Immunol. *17*, 844–850.

Waterhouse, A.M., Procter, J.B., Martin, D.M., Clamp, M., and Barton, G.J. (2009). Jalview Version 2—a multiple sequence alignment editor and analysis workbench. Bioinformatics *25*, 1189–1191.

Xu, C., Gagnon, E., Call, M.E., Schnell, J.R., Schwieters, C.D., Carman, C.V., Chou, J.J., and Wucherpfennig, K.W. (2008). Regulation of T cell receptor activation by dynamic membrane binding of the CD3epsilon cytoplasmic tyrosine-based motif. Cell *135*, 702–713.

Yokosuka, T., Sakata-Sogawa, K., Kobayashi, W., Hiroshima, M., Hashimoto-Tane, A., Tokunaga, M., Dustin, M.L., and Saito, T. (2005). Newly generated T cell receptor microclusters initiate and sustain T cell activation by recruitment of Zap70 and SLP-76. Nat. Immunol. *6*, 1253–1262.

Yousefi, O.S., Günther, M., Hörner, M., Chalupsky, J., Wess, M., Brandl, S.M., Smith, R.W., Fleck, C., Kunkel, T., Zurbriggen, M.D., et al. (2019). Optogenetic control shows that kinetic proofreading regulates the activity of the T cell receptor. eLife *8*, e42475.

Zech, T., Ejsing, C.S., Gaus, K., de Wet, B., Shevchenko, A., Simons, K., and Harder, T. (2009). Accumulation of raft lipids in T-cell plasma membrane domains engaged in TCR signalling. EMBO J. *28*, 466–476.

STAR★METHODS

KEY RESOURCES TABLE

REAGENT or RESOURCE	SOURCE	IDENTIFIER
Antibodies		
Mouse anti-human AF647 CD3ζ pY142 mAb	BD Bioscience	Cat # 558489; RRID:AB_647152
Mouse anti-human PE CD3ζ pY142 mAb	BD Bioscience	Cat# 558448, RRID:AB_647237
Mouse anti-human AF647 CD3ε mAb	BioLegend	Cat# 300422, RRID:AB_493092
Mouse anti-human BV421 CD3ε mAb	BioLegend	Cat# 300434, RRID:AB_10962690
Mouse anti-human PE CD3ε mAb	BioLegend	Cat# 300456, RRID:AB_2564150
Mouse biotinylated anti-human CD3ε mAb	BioLegend	Cat# 300404, RRID:AB_314058
Rabbit anti-human AF647 pErk mAb	Cell Signaling Technology	Cat# 4375, RRID:AB_10706777
Rabbit anti-pTyr416 Src polyclonal Ab	Cell Signaling Technology	Cat# 2101, RRID:AB_331697
Rabbit anti-human ERK 1/2 polyclonal Ab	Cell Signaling Technology	Cat# 9107, RRID:AB_10695739
Rabbit anti-HA mAb	Cell Signaling Technology	Cat# 3724, RRID:AB_1549585
Rabbit anti-human CD3 γ mAb	Abcam	Cat # 134096
Rabbit anti-human CD3 δ polyclonal Ab	GeneTex	Cat# GTX105811, RRID:AB_11166991
Mouse AF488 anti-HA mAb	Cell Signaling Technology	Cat# 2350, RRID:AB_491023
Mouse AF647 anti-HA mAb	Cell Signaling Technology	Cat# 3444, RRID:AB_10693329
Rat anti-human CD3ε mAb	Cell Signaling Technology	Cat# 4443, RRID:AB_560945
Mouse anti-CD3ζ mAb	Santa Cruz Biotechnology	Cat# sc-1239, RRID:AB_627020
Rabbit anti-CD3ζ pY142 mAb	Abcam	Cat# ab68235, RRID:AB_11156649
Mouse anti-Actin mAb	Merck Millipore	Cat# MAB1501, RRID:AB_2223041
Mouse anti-pTyr mAb	Merck Millipore	Cat# 05-321, RRID:AB_309678
Mouse anti-human CD3ε mAb	BioXCell	Cat# BE0231, RRID:AB_2687713
Mouse anti-HLA-A, B, C mAb	BioLegend	Cat# 311423, RRID:AB_1877080
Mouse anti-human APC HLA-A2 mAb	BioLegend	Cat# 343308, RRID:AB_2561567
Rabbit anti-CD3ζ polyclonal Ab	San José et al., 1998	N/A
Bacterial and virus strains		
E.coli DH5α Competent Cells	Thermo Fisher	Cat # 18265-017
E.coli Stbl3 Competent Cells	Invitrogen	Cat # C7373-03
Chemicals, peptides, and recombinant proteins		
10X Tris/Glycine/SDS	Biorad	Cat # 161-0772
16% Formaldehyde (w/v), Methanol-free	Thermo Fisher	Cat # 28908
2-Mercaptoethylamine-*HCl*	Thermo Fisher	Cat # 20408
Annexin-V-AF647	BioLegend	Cat # 640912
Annexin-V-PE	BioLegend	Cat # 640908
Alexa Fluor 488 C_5 Maleimide	Thermo Fisher	Cat # A10254
Alexa Fluor 647 C_2 Maleimide	Thermo Fisher	Cat # A20347
Amicon Ultra 15 mL centrifugal filter units	Millipore	Cat # UFC901008
Anti-HA-conjugated agarose beads	Sigma Aldrich	Cat # AL2095
A770041 Lck inhibitor	Axon Medchem	Cat # 1698
BamH1	New England Biolabs	Cat # R0136
BD Cytofix buffer	BD Biosciences	Cat # 554655
BD PhosFlow Perm/Wash buffer 1	BD Biosciences	Cat # 55785
Benzonase Nuclease	Millipore	Cat # 71206-25KUN
BirA kit	Avidity	Cat # BirA500
Bovine serum albumin (BSA)	Sigma Aldrich	Cat # A4503-100G

(*Continued on next page*)

CellPress
OPEN ACCESS

Continued

REAGENT or RESOURCE	SOURCE	IDENTIFIER
Bug Buster protein extraction reagent	Millipore	Cat # 70584-4
Catalase	Sigma Aldrich	Cat # C100
CellTrace^{TM} Violet	Thermo Fisher	Cat # C34557
Centricon® Plus-70 centrifugal filter units	Millipore	Cat # UFC703008
Cysteamine MEA	Sigma Aldrich	Cat # 30070
Disposable PD 10 desalting column	G&E Healthcare	Cat # 17-0851-01
DMEM	Sigma Aldrich	Cat # D6429
DMSO	Sigma Aldrich	Cat # D8418-100ML
Doxycycline hyclade	Sigma Aldrich	Cat # D9891-10G
DpnI	New England Biolabs	Cat # R0176
EasySep Human CD8 T cell isolation kit	STEMCELL	Cat # 17953
EcoR1	New England Biolabs	Cat # R0101
Endoglycosidase H (endo H)	New England Biolabs	Cat # P0702S
EZ-Link Maleimide-PEG2 biotin	Thermo Fisher	Cat # 21901BID
Fetal bovine serum (FBS)	GIBCO	Cat # 10500-064
Glucose	Sigma Aldrich	Cat # G8270
Glucose Oxidase	Sigma Aldrich	Cat # G2133
Glutamine	GIBCO	Cat # A2916801
HA peptide	Sigma Aldrich	Cat # I2149
His-Pur Cobalt Resin	Thermo Fisher	Cat # 89964
His-Streptavidin (AA 25-183) protein (His tag)	Antibodies Online	Cat # ABIN666648
HLA-A2-SLYNTIATL (6I-A2) monomer	Cole et al., 2017	N/A
Human T-activator CD3/CD28 Dynabeads	Thermo Fisher Scientific	Cat # 11161D
Human serum	Sigma Aldrich	Cat # H4522
ICAM-1 protein, recombinant human	R&D Systems	Cat # 720-IC-050
Imidazole	Sigma Aldrich	Cat # I202-500G
Iodoacetamide	Sigma Aldrich	Cat # I6125
Ionomycin	Sigma Aldrich	Cat # I0634
Lysonase (Lysozyme + Benzonase)	Millipore	Cat # 71230-3
MART-1 peptide (> 95% purity by HPLC)	Cambridge Peptides Inc.	N/A
n-Dodecyl β-D-maltoside (DDM)	Millipore	Cat # 324355-5GM
Non-essential amino acids solution	GIBCO	Cat # 11140050
Not 1	New England Biolabs	Cat # R0189
NuPAGE LDS Sample Buffer	Invitrogen	Cat # NP0007
NuPAGE Sample Reducing Agent	Invitrogen	Cat # NP009
NY-ESO-1 peptides (> 95% purity by HPLC)	Cambridge Peptides Inc.	N/A
PEG-*it*^{TM}	SBI	Cat # LV810A-1
PEIpro	Polyplus	Cat # 115-010
Pepsin	Thermo Fisher	Cat # 20343
Phosphate Buffer Saline (PBS)	Sigma Aldrich	Cat # P4417
Phorbol 12-myristate 13-acetate (PMA)	Sigma Aldrich	Cat # P1585
Phycoerythrin-conjugated streptavidin	Sigma Aldrich	Cat # S4762-5MG
Pme1	New England Biolabs	Cat # R0560
Polybrene	Sigma Aldrich	Cat # 28728554
Proteases inhibitors (cOmplete, EDTA free)	Roche	Cat # 11873580001
Protein G agarose	Thermo Fisher	Cat # 15920
Puromycin	GIBCO	Cat # A11138-03
RPMI 1640	Sigma Aldrich	Cat # R8758
Sodium Acetate	Thermo Fisher	Cat # 10000500

(*Continued on next page*)

Continued

REAGENT or RESOURCE	SOURCE	IDENTIFIER
Sodium Fluoride	Sigma Aldrich	Cat # S1504-500G
Sodium Orthovanadate	New England Biolabs	Cat # 11873580001
Sodium Pyruvate	GIBCO	Cat # 11360070
Strep-Tactin Sepharose 50% suspension	IBA Lifesciences	Cat # 2-1201-010
Streptavidin Sepharose High Performance beads	GE Healthcare	Cat # 17-5113-01
Tetracycline-free FBS	Clontech	Cat # 631106
Trans-Blot Turbo Midi Nitrocellulose Transfer Packs	Biorad	Cat # 170-4159
Trans-Blot Turbo Mini Nitrocellulose Transfer Packs	Biorad	Cat # 170-4158
Trizma base	Sigma	Cat # T6066
Xba1	New England Biolabs	Cat # R0145
Zeba Spin Desalting	Thermo Fisher	Cat # 89889
0.45 μm sterile filters	Sartorius Stedim	Cat # 1655-K
8 well chamber Glass μ-slide	Ibidi	Cat # 80821
96-well V-bottom plates	Thermo Fisher	Cat # 634-0009
Critical commercial assays		
QIAprep Spin Midiprep Kit	QIAGEN	Cat # 27106
QIAprep Spin Miniprep Kit	QIAGEN	Cat # 12243
BP Clonase II kit	Thermo Fisher	Cat # 11791020
LR Clonase II kit	Thermo Fisher	Cat # 11789020
Experimental models: Cell lines		
Human: TCRβ^{-} CD8^{+} Jurkat 31.13; (J13.31)	Alcover et al., 1990	N/A
Human: TCRα^{-}β^{-}/CD8^{-} Jurkat J76; (CD8^{-} J76)	Heemskerk et al., 2003	N/A
Human: TCRα^{-}β^{-}/CD8^{+} Jurkat J76;(J76)	This study	N/A
Human: 1G4α^{WT}β^{WT} J76 CD8^{-};(CD8^{-} J76 1G4 WT)	This study	N/A
Human: 1G4α^{WT}β^{WT} J76 CD8^{+};(J76 1G4 WT)	This study	N/A
Human: 1G4α^{WT}β^{A291} J76 CD8^{-}; (CD8^{-} J76 1G4 βA291)	This study	N/A
Human: 1G4α^{WT}β^{A291} J76 CD8^{+}; (J76 1G4 βA291)	This study	N/A
Human: 1G4α^{WT}β^{F291} J76 CD8^{-}; (CD8^{-} J76 1G4 βF291)	This study	N/A
Human: 1G4α^{WT}β^{L291} J76 CD8^{-}; (CD8^{-} J76 1G4 βL291)	This study	N/A
Human: 1G4α^{WT}β^{A293} J76 CD8^{-}; (CD8^{-} J76 1G4 βA293)	This study	N/A
Human: 1G4α^{WT}β^{A303} J76 CD8^{-}; (CD8^{-} J76 1G4 βA303)	This study	N/A
Human: 2H5α^{WT}β^{WT} J76 CD8^{-};(J76 CD8^{-} 2H5 WT)	This study	N/A
Human: 2H5α^{WT}β^{A291} J76 CD8^{-};(J76 CD8^{-} 2H5 βA291)	This study	N/A
Human: 1G4α^{WT}β^{WT} CD3ζ$^{-/-}$ J76 CD8^{+}; (J76-1G4WT-ζKO)	This study	N/A
Human: 1G4 α^{WT}β^{WT} CD3ζ$^{-/-}$ J76 CD8^{+} expressing CD3ζWT; (J76-1G4WT-ζKO expressing ζWT)	This study	N/A
Human: 1G4 α^{WT}β^{WT} CD3ζ$^{-/-}$ J76 CD8^{+} expressing ζI38A; (J76-1G4WT-ζKO expressing ζI38A)	This study	N/A
Human: 1G4 α^{WT}β^{WT} CD3-ζ$^{-/-}$ J76 CD8^{+} expressing ζI41A; (J76-1G4WT-ζKO expressing ζI41A)	This study	N/A
Human: 1G4 wtc51 J76 CD8^{+}; (J76 wtc51)	This study	N/A
Human: 1G4 wtc51 J76 CD8^{-}; (CD8^{-} J76 wtc51)	This study	N/A

(*Continued on next page*)

Continued

REAGENT or RESOURCE	SOURCE	IDENTIFIER
Human: 1G4 QM-α J76 CD8^{+}; (QM-α)	This study	N/A
Human: 868 J76 CD8^{+}; (J76 868)	This study	N/A
Human: 868 J76 CD8^{-}; (CD8^{-} J76 868)	This study	N/A
Human CD8^{+} primary T cell 1G4 wtc51	This study	N/A
Human: 1G4 $\alpha^{WT}\beta^{WT}$ CD3$\zeta^{-/-}$ expressing CD3ζ-TST (J76 1G4 ζ-TST)	This study	N/A
Human: HEK293T	ATCC	Cat # CRL-3216
Human: Lenti-X293T	Clontech	Cat # 632180
Oligonucleotides		
See Table S2	This study	N/A
Recombinant DNA		
pDONR-221	Thermo Fisher scientific	Cat # 12536017
pEF3 FLAG	K. Nika, University of Oxford	N/A
pEF3 HA	K. Nika, University of Oxford	N/A
pEXPR-IBA103	IBA Lifesciences	Cat # 2-3503-000
pHR-CD8$\alpha\alpha$	A. Van Der Merwe, University of Oxford	N/A
pHR-SIN-BX-IRES-Emerald	V. Cerundolo, University of Oxford	N/A
pLIX-402	Addgene	Cat # 41394
pLEX_307	Addgene	Cat # 41392
psPAX2	Addgene	Cat # 12260
pVSV-G	Addgene	Cat # 14888
Software and algorithms		
Flow Jo	BD	N/A
GROMACS	http://www.gromacs.org/	N/A
Image Studio	Li-COR Bioscience	N/A
Insight3	B. Huang, University of California, San Francisco	N/A
Jalview	Waterhouse et al., 2009	N/A
MATLAB software	S. Shelby and S. Veatch, University of Michigan	N/A
OriginPro 2017	OriginLab	N/A
Prism	GraphPad Software	N/A
PyMOL	Schrödinger LLC	N/A
SnapGene	GSL Biotech LLC	N/A
Other		
Hi-Load 16/600 Superdex 200 pg Column	GE Healthcare	Cat # 28-9893-35
Superdex-200 Increase 5/150 GL Column	GE Healthcare	Cat # 28-9909-44

RESOURCE AVAILABILITY

Lead contact

Further information and requests for resources and reagents should be directed to and will be fulfilled by the lead contact, Oreste Acuto (oreste.acuto@path.ox.ac.uk).

Materials availability

There are no restrictions on any data or materials presented in this paper. Requests for unique resources and reagents generated in this study should be directed to and will be fulfilled by the lead contact. Please see the key resources table for commercially available materials.

Data and code availability

No unpublished custom code was generated in this study. Links to software and algorithms used in this study are indicated in the key resources table.

EXPERIMENTAL MODEL AND SUBJECT DETAILS

Cell lines

Cell lines were maintained at 37°C with 5% CO_2 in a humidified incubator (Heraeus). Human embryonic kidney epithelial 293T cells HEK293T (ATCC CRL-3216) and Lenti-X293T (Clontech) cell lines were cultured in complete DMEM (Sigma Aldrich) supplemented with 15% fetal bovine serum (FBS). Jurkat-derived cell line variants 31.13 (Alcover et al., 1990) (abbreviated J31.13) and J76 (Heemskerk et al., 2003), which lack expression of TCRβ and TCRαβ respectively, were cultured in RPMI 1640 supplemented with 10% FBS. Key resources table lists all the above cell lines and those that were derived from J31.13 and J76 in this study. Cells were routinely tested and found negative for mycoplasma and are not STR profiled. Jurkat cell lines containing a tetracycline-inducible gene expression systems were maintained in RPMI 1640 supplemented with 10% tetracycline-negative FBS (Clontech).

Primary T cells

Primary CD8 T cells were obtained from HLA-A2^{-} healthy donor blood cones (NHS) by density gradient centrifugation and negative selection using EasySep Human CD8 T cell isolation kit (STEMCELL). Isolated T cells were rested overnight at 2 × 10^6/ml in RPMI medium (Sigma) supplemented with 2 mM Glutamine, 1 mM Sodium Pyruvate, Non-essential AA, 50 μg/ml Kanamycin, 5% human serum (Sigma).

METHOD DETAILS

DNA constructs and cloning

All plasmid DNA was propagated in *E. coli* strains DH5α or Stbl3.

Generation of 1G4 α and 1G4 β plasmids for transient transfections

1G4 α and β sequences were amplified from total cDNA extracted (Roche cDNA extraction kit) from an established 1G4 expressing Jurkat cell line (Aleksic et al., 2010) with appropriate primers (see primers in Table S2) and cloned into pEF3 with a Flag- and HA-tag respectively.

Site directed mutagenesis

Single amino acid mutations in the 1G4 β chain were introduced using a modified QuikChange protocol (Agilent). Briefly, PCR primers were generated using the QuikChange open source software. PCR was carried out with an optimized protocol and the resulting product was purified using QiaQuick Gel purification kit (QIAGEN). A fraction of purified PCR was digested with DpnI for 2 h and used to transform Stbl3 *E. coli* (Invitrogen). DNA was extracted from colonies, analyzed by gel electrophoresis and sequenced (Source BioScience. UK).

Generation of the self-cleavable 1G4 αβ construct (stable cell lines)

A DNA construct was designed to obtain a single mRNA for β and α giving rise to a single polypeptide separated by the foot-and-mouth disease virus 2A (F2A) self-cleavable sequence (Ryan et al., 1991). This strategy should facilitate expression of similar amounts of β and α proteins. The aa sequence of the entire construct is provided in Table S1. See Figure S1A for a schematic of the construct.

Constructs for Tet-inducible expression of 1G4 αβ, 2H5 αβ, ζ WT and mutants, affinity enhanced 1G4 (wtc51 and QM-α), ζTST and constitutive 1G4 αβ and 868 TCR

To generate an inducible expression system for 1G4 αβWT and 1G4 αWT/βA291, the sequence coding for the self- cleavable single polypeptide β-HA-F2A-α-FLAG was cloned into the doxycycline inducible Gateway cloning plasmid pLIX-402 (Addgene) following the manufacturer's guidelines for Gateway cloning. Briefly, the DNA sequence containing the entire αβ sequence was PCR amplified from the constitutive expression plasmid pHR with primers containing the Gateway recombination sites (see primers for Gateway cloning in Table S2). Purified PCR products were inserted via the BP recombination reaction into the Gateway entry vector pDONR-221 (Thermo Fisher). After verification of successful recombination by automated sequencing (Source Bioscience, UK), the resulting entry clones were used for a LR recombination reaction to insert the sequence into the pLIX-402 destination vector (Addgene), which carries a tetracycline-inducible promoter for conditional expression of the gene of interest. Both BP and LR reactions were performed with BP and LR Clonase II kits (Thermo Fisher). To clone the high affinity TCRs (wtc51 and QM-α) and the entire panel of 1G4 and 2H5 β and ζ mutants, synthetic DNA fragments coding for either self-cleavable single polypeptide 1G4 αβ (αWT plus βWT or mutants βA291, βL291, βF291) or 2H5 WT and 2H5 βA291 or ζWT and mutants ζA38 and ζA41 were purchased from GeneArt Gene Synthesis (Thermo Fisher). All DNA fragments contained consensus sequences for Gateway Cloning System and the αβ and ζ constructs were introduced into the Gateway plasmid pLIX-402 for inducible expression, following the Gateway cloning protocol as described above. After cloning into the expression vectors, the coding sequences were verified by automated sequencing (Source BioScience, UK). To generate a twin-strep tagged (TST) ζ containing cell-line the sequence of ζ was cloned, after XbaI/EcoRI digestion, into the plasmid pEXP103 IBA (IBA Lifesciences), to be fused with a TS-tag at the C terminus. The ζTST sequence was subsequently amplified and cloned into pLIX-402 destination vector following the gateway procedure as described above. (See primers for

Gateway cloning in Table S2). To generate a constitutive expression system for 1G4 αβ and 868 αβ synthetic DNA fragments coding for either self- cleavable single polypeptide 1G4 αβ and 868 αβ were purchased from GeneArt Gene Synthesis and were introduced into the Gateway plasmid pLEX 307 (Addgene) for constitutive expression, following the Gateway cloning protocol as described above.

CRISPR/Cas9-mediated ζ knock-out

CRISPR/Cas9 mediated knock-out (KO) of ζ was achieved using the pX458 two component vector (Addgene) and the guide sequence CAGGCACAGTTGCCGATTACAGG. J76 expressing CD8 and the single polypeptide 1G4 αβ were transiently transfected and CD3 negative cells were sorted. Efficient knock-out was verified by transient transfection of the proteins of interest and analysis of rescue by FACS analysis.

TCR-CD3 expression

Transient expression of TCR-CD3

J31.13 cells were grown to approximately 0.5 – 0.6 × 10^6/ml, cells were counted, harvested (2 × 10^6 cells per electroporation) and centrifuged at 1,200 rpm. The supernatant (conditioned medium) was collected, diluted with equal volume of RPMI containing 10% FBS and kept at 37°C in the incubator. Cells were washed twice in RPMI and re-suspended in 400 μL RPMI and placed in sterile Biorad Genepulser Cuvettes (0.4 cm) containing 2 μg of plasmid DNA per 10^6 cells. Constructs and mutant DNA were routinely purified from *E. coli* using QIAGEN mini- or midi-prep kits (QIAGEN). Electroporation was performed with a Biorad GenePulser X-cell at 260 V, 1 pulse of 25 ms using the square root method. Cells were flushed out of the cuvette with 200 μL conditioned medium and placed at 37°C in the incubator until final use. Cells were usually assayed after 24 - 48 h and analyzed by flow cytometry for CD3 surface expression or for pErk in response to pMHC tetramer.

Generation of stable cell lines

For lentivirus infection, recipient cells were cultured up to approximately 0.5 – 0.6 × 10^6/ml. Cells were washed once in RPMI, checked for viability, counted and adjusted to 10^6/ml in RPMI supplemented with 10% FBS (tetracycline-free (Tet$^-$) in the case of transduction with doxycycline-inducible plasmids) (GIBCO or Clontech) and 5 μg/ml of polybrene (Sigma). Cells were plated in 12-well (1 ml/well) or 6-wells plates (2 ml/well) and one aliquot of lentivirus was added (for preparation of lentiviruses see below). 24 h post-infection, cells were washed and re-suspended in RPMI 10% FBS (standard or Tet$^-$). After transduction with a plasmid containing an antibiotic selection marker, cells were cultured for an additional 24 h before starting selection with puromycin (1 μg/ml, GIBCO). With adequate lentivirus concentration, cell mortality was relatively low.

For tetracycline-inducible cell lines, gene expression could be started after 72 h by adding 0.1 - 1 μg/ml of doxycycline hyclate (Sigma Aldrich). Cells were collected after approximately 16 h and tested for CD3 expression and signaling by flow cytometry.

Primary T cells were plated in 24-well (1 ml/well) at 1 × 10^6/ml and stimulated with human T-activator CD3/CD28 Dynabeads (Thermo Fisher Scientific) at 2:1 bead to T cell ratio in complete medium supplemented with 500 U/ml IL-2. After 48 h most of the medium was removed and replaced with fresh medium supplemented with 500 U/ml IL-2, containing 40 μL of concentrated lentiviral stock in presence of 5 μg/ml polybrene (Sigma). After 4 days the dynabeads were removed and 1G4 wtc51 positive cells were sorted after $(9V\text{-}A2)_4$-PE staining. Sorted cells were kept in complete medium supplemented with 500 U/ml IL-2 and re-stimulated with human T-activator CD3/CD28 Dynabeads 12 days after the sorting.

Production of lentiviral particles

Lentiviruses were generated using the packaging cell lines HEK293T or Lenti-X293T. The culture medium was exchanged with RPMI with 10% FBS just prior to transfection. HEK293T or Lenti-X293T at 80% confluence were transfected using PEIpro (Polyplus) according to the manufacturer's instructions or by a standard calcium-phosphate precipitation protocol. The packaging plasmids pVSVG and pSPAX2 were mixed with the lentivirus expression vectors containing the gene of interest. For the PEIpro transfection, PEIpro solution was added to the plasmids mix and immediately vortexed, left 15 min at room temperature (RT) and then added dropwise to the cells by gently swirling the plate. For calcium-phosphate precipitation, cells were left 3 h with DNA-calcium-phosphate precipitate and the media replaced with complete DMEM with 15% FBS. Independently of the transfection protocol, supernatant containing lentiviral particles was collected after 48 h and filtered through a 0.45 μm sterile filter (Sartorius Stedim). Lentivirus supernatants were either used immediately or concentrated with PEG-*it*™ (SBI) concentration kit according to the manufacturer's instruction. Briefly, lentiviral supernatants were mixed with Virus Precipitation Solution (SBI) to a final concentration of 1X Virus Precipitation Solution and incubated overnight at 4°C followed by a centrifugation at 1,500 x g for 30 min at 4°C. The pellet containing lentivirus particles was re-suspended in 1/100 of the volume of the original cell culture using cold RPMI. Aliquots were immediately frozen in cryogenic vials at - 80°C and stored until use. Aliquots of each lentivirus batch were routinely pre-tested by serial dilution titration. Frozen aliquots were thawed only once and used immediately with minimal loss of virus titer as determined by flow cytometry.

Preparation of pMHC monomers and tetramers

pMHC monomers were produced as described elsewhere (Altman and Davis, 2003) with some modifications. Human beta-2 microglobulin (β2 m) and the 1 - 278 segment of HLA-A*02:01 heavy chain with the AviTag at the C terminus were separately expressed in *E. coli*. Both proteins were recovered from inclusion bodies with Bug Buster protein extraction reagent (Millipore) supplemented with

lysozyme and benzonase (Millipore). For monomer refolding, HLA heavy chain and β2 m were added to refold buffer (100 mM Tris, 400 mM L-arginine hydrochloride, 2 mM EDTA, 5 mM reduced glutathione, 0.5 mM oxidised glutathione, 0.1 mM PMSF) supplemented with 0.5 mM urea and 10 μg/ml synthetic peptide (> 95% purity by HPLC) of either one the following variants of the NY-ESO-1 antigen (Aleksic et al., 2010) (SLLMWITQC, residues 157-165): 9V (SLLMWITQV, K_d 7.2 ± 0.5 μM), 6V (SLLMWVTQV, K_d 18 μM) or the MART-1 tumor antigen (MART-1 26-35 (ELAGIGILTV) (Circosta et al., 2009) or the 6I (SLYNTIATL, Cambridge Peptides Inc.). The resulting soluble-monovalent-monodisperse pMHC (sm-pMHC) were indicated as: 9V-A2, 6V-A2, MART-1-A2 and 6I-A2.

Peptides were synthesized by standard solid-phase chemistry using F-moc for transient N-terminal protection. All peptides were approximately 98% pure as determined by analytical HPLC and mass spectrometry. Lyophilized peptides were dissolved in DMSO (Sigma) to 10 mg/ml and aliquoted of 100 μL in low-bind Eppendorf tubes and used immediately or stored at - 80°C. The refold mixture was stirred gently for 40 h at 4°C, concentrated approximately 40-fold to 2.5 mL by centrifugation at 4°C at 3,500 x g in Centricone® Plus-70 centrifugal filter units (30 kD cut-off) (Merck Millipore) and desalted on a disposable PD 10 column (GE Healthcare) by gravity flow eluting in 3.5 mL TBS. The eluent was subject to mono-biotinylation using a BirA kit (Avidity) and fractionated by FPLC using a HiLoad™ 16/600 Superdex 200 pg column (GE Lifesciences) and the ÄKTA Pure (GE Healthcare) system. Fractions containing rHLA-A*02:01-β2 m dimers were pooled and concentrated in Amicon Ultra 15 mL centrifugal filter units (10 kD cut-off) (Merck Millipore). After addition of glycerol to a final concentration of 5%, protein concentration was adjusted to 1 mg/ml measuring OD_{280} and aliquots were frozen at - 80°C until use. We rigorously controlled the quality of monomers prior to performing experiments by size-exclusion chromatography (SEC) and we also used multi-angle light scattering with size-exclusion chromatography (SEC-MALS) for sm-9V-A2 to further control for mono-dispersity and unique molecular mass in the peak.

Tetramers were generated by slowly mixing aliquots of biotinylated rHLA-A*02:01-rβ2 m with phycoerythrin (PE)-conjugated streptavidin (Sigma) at RT under constant agitation as described earlier (Altman and Davis, 2003). Alternatively, His-streptavidin (Antibodies Online) was used. The resulting tetramers, $(9V\text{-}A2)_4$, $(6V\text{-}A2)_4$, $(MART\text{-}1\text{-}A2)_4$ and $(6I\text{-}A2)_4$, were stored at 4°C until final use.

Preparation of UCHT1-Fab' fragment

A Fab' fragment was prepared from the purified mAb UCHT1 (BioXCell). Briefly, UCHT1 was digested with Pepsin (Thermo Fisher) in 100 mM Sodium Acetate (Thermo Fisher) for 24 h at 37°C on a rocking platform. The digest was incubated overnight at 4°C on a rotating shaker with Protein G agarose (Thermo Fisher) to remove the Fc fragment. The resulting Fab_2 was reduced with 2-Mercaptoethylamine-HCl (MEA-HCl) (Thermo Fisher) for 90 min at 37°C, according to the manufacturer's instructions, to obtain the Fab' fragment. MEA-HCl excess was removed using a Zeba Spin Desalting column (Thermo Fisher) and the Fab' fragment eluted in Phosphate Buffered Saline (PBS, Sigma) at pH 7.0. The UCHT1-Fab' was conjugated to biotin using EZ-Link Maleimide-PEG2 biotin (Thermo Fisher) or to Alexa Fluor 647 or 488 using Alexa Fluor 647 C_2 Maleimide and Alexa Fluor 488 C_5 Maleimide respectively (Thermo Fisher). The Fab' was mixed in 1:10 molar ratio with 2 mM EZ-Link Maleimide-PEG2 biotin or with Alexa Fluor 647 C_2 Maleimide for 5 h with slight agitation on a rocking platform at RT. Reagent excess was removed using a Zeba Spin Desalting column. The biotin, Alexa Fluor 488 or Alexa Fluor 647-labeled UCHT1-Fab' fragments were analyzed on a non-reduced 4 - 15% Mini-PROTEAN Precast protein gel (Bio-Rad) and stored in PBS at 4°C. The biotin-labeled UCHT1-Fab' was further analyzed by FPLC to detect formation of Fab' aggregates. Biotin-labeled UCHT1-Fab' was diluted to a concentration of 0.3 mg/ml in PBS and 40 μl were analyzed by size exclusion chromatography. The analysis was carried out using a Superdex 200 increase 5/150 GL column (GE Lifesciences) and the ÄKTA Pure system (GE Lifesciences). Fab' was applied onto the column using a 40 μl loop at a flow rate of 100 μl/ml. Isocratic elution with PBS occurred at 300 μl/min with a total elution volume of 3 ml.

Flow cytometry

General staining procedures

This section provides a description of the procedures used for antibody (Ab)-mediated cell staining for flow cytometry and data acquisition. Staining was performed in 96-well V-bottom plates (Thermo Fisher) and centrifugations carried out at 670 x g for 1 min in a plate centrifuge (Eppendorf Centrifuge 5810R or Thermo IEC CL30 Centrifuge). Care was taken to gently re-suspend cells after each centrifugation to avoid cell damage and/or death. To optimize the sensitivity of the flow cytometry-based assays, Abs used in this study were systematically titrated for staining of cells expressing or lacking the target antigen and the conditions showing the best signal-to-noise were chosen. Flow cytometry staining was routinely carried out in duplicates or triplicates. For cell surface staining, 0.1×10^6 cells/sample were transferred into a 96-well V-bottom plate, washed once in 150 μL FACS buffer (0.5% bovine serum albumin (BSA) (Sigma) in PBS). After spinning, supernatants were removed and cell pellets re-suspended in 50 μL staining solution containing fluorescence-conjugated primary Ab (see Key resources table for a list of the Abs used in this study) diluted in FACS buffer and incubated for 20 min at RT or for 30 min at 4°C, depending on the Ab used. After removal of the staining solution, samples were washed twice with 150 μL FACS buffer and flow cytometry data acquired immediately or cells were fixed with 150 μL pre-warmed fixation solution (BD Cytofix®, BD Biosciences) for 10 min at 37°C. For staining of intracellular antigens, fixed samples were washed twice in 150 μL permeabilisation buffer (BD Perm/Wash I, BD Biosciences), re-suspended in 150 μL permeabilisation buffer and incubated at RT for 30 min. Permeabilised cells were stained in 50 μL permeabilisation buffer containing the desired Ab dilution. For fluorescent-conjugated primary Ab staining, samples were incubated at RT for 1 h (except for anti-pY142 CD3ζ and anti-HA, which were incubated at 4°C for 2 h). When fluorescent-conjugated secondary Abs were used, they were diluted in 50 μL permeabilisation buffer and added to cells for 20 min at RT. After each staining, cells were washed 3 times with 150 μL permeabilisation

buffer and once with 150 μL FACS buffer at the end of the staining procedure. When surface co-staining was included, it was performed prior to fixation as described above. Samples were left in FACS buffer for storage and acquisition and acquired on a CyAn ADP analyzer (Beckman Coulter) or BD LSR Fortessa X20 (BD Bioscience) as specified. Raw data was analyzed by FlowJo (FlowJo Software part of BD). Counts, percentages or median intensity fluorescence values (MFI) were extracted from FlowJo as excel files. Statistical analysis and non-linear regression were performed with Prism (GraphPad Software).

CellTrace violet labeling

Cells were washed once in PBS and adjusted to a final concentration of 10^6 cells/ml in pre-warmed PBS at 37°C. CellTrace violet (Thermo Fisher) or carrier control DMSO (Sigma) was added to reach the indicated staining concentration and cells were incubated at 37°C in the dark. After 20 min, samples were diluted 5-fold in complete medium and incubated for an additional 5 min at 37°C in the dark. After removal of the diluted staining solution, cells were re-suspended in complete medium, counted and cultured or mixed as indicated.

UCHT1-Fab' fragment binding

J76 1G4 WT or β mutants were induced for TCR expression with doxycycline 1 μg/ml for 96 h. TCR-deficient J76 $CD8^+$ cells were used to evaluate the background. Cells were harvested and stained with AF488-conjugated UCHT1-Fab' at the indicated dilutions in FACS buffer for 30 min at 4°C. After removing excess of UCHT1-Fab', cells were fixed, permeabilised and intracellular staining was performed with Alexa647-conjugated anti-HA mAb (1:50) as described in the flow cytometry protocol section. Samples were acquired on CyAn ADP analyzer (Beckman Coulter). Raw data was analyzed in FlowJo and MFI of UCHT1-Fab' binding in the TCRβ-HA positive population extracted as excel files. Further analysis and statistical tests were performed in Prism (GraphPad Software). MFI of UCHT1-Fab' binding after background subtraction was plotted against the concentration of UCHT1-Fab' and fitted with non-linear regression (One site - Specific binding).

pMHC-tetramer binding to cells for association and dissociation analysis

To measure the relative association rate of pMHC tetramers to WT and βA291 TCR, J76 $CD8^+$ 1G4 WT or βA291 cells were induced for TCR expression with 1 μg/ml doxycycline for 48 - 72 h. TCR-deficient J76 cells were used to evaluate binding background. Cells were labeled with 200 nM CellTrace violet (Thermo Fisher) or with carrier control DMSO (Sigma), mixed 1:1 and washed once before being re-suspended at 20 × 10^6/ml in RPMI, pre-warmed at 37°C. Cells were then distributed at 0.5 × 10^6 cells, 25 μl/well in a 96-well V-bottom plate (Eppendorf) and equilibrated at 37°C for 10 min in a ThermoMixer C (Eppendorf). 1:2 dilution series of 2X $(6V\text{-}A2)_4$-PE were added directly to the wells to a final concentration of 400 to 3.125 nM as indicated in the plot. After 10 min binding, a 3-fold excess of fixation buffer (BD Cytofix) was added and samples fixed for 10 min at 37°C. Samples were permeabilised and stained intracellularly with anti-HA (1:50) to detect total TCRβ as described in Flow cytometry protocols. To measure the relative dissociation rate of pMHC tetramers from WT and βA291 TCR, the same procedure as for on-rate measurements was used. This included cells, CellTrace violet labeling procedure, binding conditions and staining procedure, except that the assay was performed in 1.5 mL Eppendorf tubes. The concentration of $(6V\text{-}A2)_4$-PE in this assay was 50 nM. After 10 min of pMHC tetramer binding to the cells at 37°C, samples were washed once in RPMI containing 10% FBS and re-suspended at 10^6/ml in RPMI containing 10% FBS supplemented with 10 μg/ml anti-HLA-A, B, C (BioLegend) to prevent rebinding of dissociated pMHC tetramer. Samples (0.2 × 10^6 cells in 200 μl) were taken at the indicated times, washed once in FACS buffer and fixed for 2 h at 4°C in fixation buffer (BD Cytofix). Cells were permeabilised and stained for TCRβ-HA as for the binding assay. Samples were acquired on a CyAn ADP. Raw FACS data was analyzed in FlowJo (FlowJo Software) and MFI of $(6V\text{-}A2)_4$-PE binding and TCRβ-HA were extracted as excel files. Further analysis and statistical tests were performed in Prism (GraphPad Software). The MFI of the $(6V\text{-}A2)_4$-PE binding was corrected for background, normalized for maximal binding (max. binding = 100%), plotted against the concentration of $(6V\text{-}A2)_4$-PE or against the time after initial binding and the curves were fitted by non-linear regression (Specific binding with Hill slope or Dissociation - One phase exponential decay).

TCR-CD3 expression efficiency

To evaluate the efficiency of TCR-CD3 surface expression, J76 $CD8^-$ 1G4 (or 2H5) WT or β mutant cells were induced for TCR expression with 1 μg/ml doxycycline overnight at 0.3 × 10^6 cells/ml. Cells were incubated with 50 nM CellTrace violet or carrier control DMSO (Sigma), mixed in a 1:1 ratio and stained for surface CD3 and intracellular TCRβ-HA, using UCHT1 AF647-conjugated Fab' and AF488-conjugated anti-HA mAb respectively, as described in Flow cytometry procedures. J76 $CD8^-$ 1G4 (or 2H5) WT or β mutants, non-induced with doxycycline were used to evaluate binding background. Cells were analyzed on a BD LSR Fortessa X20 flow cytometer (BD Biosciences) and acquired data was analyzed with FlowJo FACS analysis software V10.0 (Tree Star, BD). 2D plots of TCRβ-HA versus CD3 were obtained and gates for different levels of TCRβ-HA (low, medium and high) were applied. The MFI ± SD of CD3 and TCRβ-HA was extracted within each gate and normalized to the WT in the corresponding gate. Statistical analysis using unpaired t test was performed using Prism (GraphPad Software). MFI ± SD of TCRβ-HA for 1G4 WT and β mutants in those bins was not statistically significant (ns). The same procedure was used to evaluate the efficiency of TCR-CD3 surface expression in J76 $CD8^+$ 1G4 ζKO expressing ζWT and ζ mutants (ζA38, ζA41).

pMHC-tetramer stimulation for pErk or pζ-dose-response

To measure proximal signaling in response to stimulation with pMHC, J76 $CD8^+$ or $CD8^-$ 1G4 (or 2H5) WT or mutant cells were induced for TCR expression with doxycycline (dox) overnight at 0.3 × 10^6 cells/ml. In all pMHC-tetramer stimulations for pErk, J76 $CD8^+$ or $CD8^-$ 1G4 WT (or 2H5 WT) were induced for TCR expression with 0.8 - 0.2 μg/ml of dox whereas β (or ζ) mutants were induced with 1 μg/ml. Different doses of doxycycline for 1G4 WT (or 2H5 WT) and β (or ζ) mutants were used in order to achieve

same TCR-CD3 surface expression and to simplify computation of signaling outputs and eliminate potential errors arising from normalizing for unequal $(6V\text{-}A2)_4$ binding. Cells were then labeled with 50 nM CellTrace violet (Thermo Fisher) or with carrier control DMSO (Sigma). CellTrace labeling did not interfere with proximal signaling events. To measure TCR surface expression a surface staining with AF647-conjugated UCHT1-Fab' fragment was performed according to the protocol described in Flow cytometry procedures before the stimulation assay. For this, labeled and unlabelled cells were mixed 1:1 and washed once before being re-suspended at 20×10^6/ml in RPMI without FBS pre-warmed at 37°C. Cells were then distributed at 0.5×10^6 cells/well (25 μl) in a 96-well V-bottom plate (Eppendorf) and equilibrated at 37°C for 10 min in a ThermoMixer C (Eppendorf). 1:2 dilution series of 2X $(6V\text{-}A2)_4$-PE or $(MART\text{-}1\text{-}A2)_4$-PE were added directly to the wells to a final concentration of 600 to 0.78 nM and 50 to 0.05 nM, respectively. After stimulation (pζ: 60 s, pErk: 180 s) an excess of fixation buffer (BD Cytofix) was added and samples fixed for 10 min at 37°C. Samples were permeabilised and stained intracellularly with AF647-conjugated anti-pErk (1:50) or with AF647-conjugated anti-pY142ζ (1:5) as described in Flow cytometry procedures. Samples were acquired on a CyAn ADP (Beckman Coulter) or BD LSR Fortessa X20 (BD Bioscience) as specified. Raw data was analyzed by FlowJo (FlowJo software, part of BD) and MFIs were extracted from FlowJo as excel files. Further analysis and statistical tests were performed with Prism (GraphPad Software). For pζ-dose-response the background subtracted MFIs of pζ were plotted against the background subtracted MFI of tetramer $(6V\text{-}A2)_4$-PE binding and fitted by non-linear regression ([Agonist] versus response - three parameters). The background subtracted values of MFIs of pζ with the highest dose of $(6V\text{-}A2)_4$-PE (400 nM) were normalized to WT or left as pairs and tested for significance with a t test (paired or unpaired respectively). For pErk-dose-responses, pErk MFIs were background subtracted and plotted against the dose of $(6V\text{-}A2)_4$-PE or $(MART\text{-}1\text{-}A2)_4$-PE and fitted by non-linear regression ([Agonist] versus response - Variable slope (four parameters) or [Agonist] versus response - three parameters) or fitted to a minimal model of kinetic proofreading signaling (see Mathematical modeling section). In both non-linear regression fits, no constrains were applied and the expected best-fit values of maximum pErk for WT and mutant were analyzed for significance with the F-test. The same procedure was used to evaluate max. pErk response upon $(6V\text{-}A2)_4$-PE titration, in J76 $CD8^+$ 1G4 ζKO expressing ζWT and ζmutants (ζA38, ζA41).

PMA stimulation

To evaluate maximal pErk potential of J76 $CD8^-$ 1G4 WT or βA291, cells were labeled with 50 nM CellTrace violet (Thermo Fisher) or with carrier control DMSO (Sigma), mixed 1:1 and washed once before being re-suspended at 20×10^6/ml in RPMI without FBS pre-warmed at 37°C. Cells were then distributed at 0.5×10^6 cells/well (25 μl) in a 96-well V-bottom plate (Eppendorf) and equilibrated at 37°C for 10 min in a ThermoMixer C (Eppendorf). 1:2 dilution of 2X PMA (Phorbol 12-myristate 13-acetate, Sigma) and Ionomycin (Sigma) was added directly to the wells to a final concentration of 66.6 ng/ml and 2 μg/ml, respectively. After stimulation (180 s) an excess of fixation buffer (BD Cytofix) was added and samples fixed for 10 min at 37°C. Samples were permeabilised and stained intracellularly with AF647-conjugated anti-pErk (1:50) according to the protocol described above. Raw data was analyzed by FlowJo (FlowJo software, part of BD) and MFIs were extracted from FlowJo as excel files. Further analysis and statistical test were performed with Prism (GraphPad Software). The background subtracted values of MFIs of pErk for WT and βA291 were exported and mean values of each experiment, measured in triplicates, were normalized to WT. Statistical significance was tested by unpaired t test using Prism (GraphPad Software).

Basal ζ-phosphorylation

J76 $CD8^+$ 1G4 WT or βA291 cells were induced for TCR expression with 1 μg/ml doxycycline at 0.3×10^6 cells/ml. Cells were harvested at 24, 48, 72 or 96 h and processed immediately for FACS analysis of CD3 surface expression or intracellular pζ according to the protocol described in Flow cytometry procedures. As staining for CD3 surface expression with an anti-CD3ε induced signaling even when carried out at 4°C using precooled solutions, samples were split in two and one part was stained for surface CD3 at 4°C while the other part was fixed at 37°C, permeabilised and analyzed for basal pζ. Samples were acquired on a CyAn ADP (Beckman Coulter). Raw data was analyzed by FlowJo (FlowJo software, part of BD) and MFIs were extracted from FlowJo as excel files. Further analysis and statistical tests were performed with Prism (GraphPad Software). pζ MFI was normalized to CD3 MFI (pζ/CD3), mean values of each experiment were normalized to WT or left as pairs and significance was tested by t test (unpaired or paired respectively).

Sm-pMHC binding to cells

To detect non-specific adsorption of sm-pMHC onto J76 cell membrane during the stimulation assay (see Soluble monovalent-pMHC stimulation section) CD8-deficient wtc51 J76 were induced or not for TCR expression with 1 μg/ml doxycycline for 48 h, labeled with 50 nM CellTrace violet (Thermo Fisher) or with carrier control DMSO (Sigma), mixed 1:1 and washed once before being re-suspended at 10×10^6 in 125 μL of pre-warmed RPMI at 37°C. Cells were then distributed in a 96-well V-bottom plate (Eppendorf) and equilibrated at 37°C for 10 min in a ThermoMixer C (Eppendorf) under constant shaking (500 rpm). Dilution series of 2X sm-9V-A2 were added directly to the wells to a final concentration of 400 to 0.2 nM as indicated in the plot. After 5 min of binding, samples were rapidly centrifuged at 4°C, washed with 100 μL of ice-cold FACS-buffer and stained with APC-conjugated anti-HLA-A2 (1:100) for 20 min on ice to detect sm-9V-A2 bound to cells as described in Flow cytometry protocols. Samples were acquired on BD LSR Fortessa X20 (BD Bioscience). Raw FACS data for TCR-deficient and TCR-efficient J76 were analyzed in FlowJo (FlowJo Software) and MFI of APC-anti-HLA-A2 were extracted as excel files. Further analysis and statistical tests were performed in Prism (GraphPad Software). The MFI of the APC-anti-HLA-A2 binding was corrected for background, plotted against the concentration of sm-9V-A2 and the curves were fitted by non-linear regression.

Mathematical modeling

To fit the tetramer binding data, we used an effective 1:1 binding model:

$$C = Bmax * \frac{[\text{Tetramer}]}{(Kd + [\text{Tetramer}])}$$

where B_{max} is the maximum binding and K_d is an effective binding constant. We found that this model was able to fit both the WT and mutant TCR data. Importantly, we found that a single value of K_d was sufficient to fit both dataset or equivalently, we found no evidence to reject the null hypothesis that tetramer binding was identical to both (F-test, p = 0.066).

To fit the pErk data, we coupled the binding model to a minimal model of kinetic proofreading signaling (Dushek et al., 2011; McKeithan, 1995)

$$Y = \frac{[Tetramer]}{Kd + [\text{Tetramer}]} * \frac{kp}{(kp + koff)^N}$$

where the first term determines tetramer occupancy (fraction between 0 and 1) and the second term determines the probability of signaling, k_p the forward rate of proofreading, N the number of steps, and k_{off} the unbinding rate.

Given that we found no evidence for a difference in binding (see above), we asked whether a difference in the proofreading rate (kp) can explain the difference in the pErk data. To do this, we simultaneously fit both the WT and mutant TCR pErk data to the mathematical model with a different value of k_p for each TCR. As above, we fit a single value of K_d for both datasets and in this model, we fixed the value of k_{off} to the experimentally determined value (0.85 s^{-1}; Aleksic et al., 2010) and fixed the value of N to the recently reported value (Tischer and Weiner, 2019; Yousefi et al., 2019). This is reasonable since we do not expect the TCR mutation to alter the number of steps.

We found that this model was able to produce an excellent fit to the data. Importantly, we found that a single value of k_p could not explain the pErk data or equivalently, we had sufficient evidence to reject the null hypothesis that a single value of k_p could explain the data (F-test, $p < 0.0001$).

Biochemical analysis of the TCR-CD3 complex

SDS-PAGE, immunoblotting and quantitation

SDS-polyacrylamide gel electrophoresis (PAGE) was performed using the protein electrophoresis system from Bio-Rad (Bio-Rad) according to the manufacturer's instructions. Custom-made 15% polyacrylamide gels were used and proteins separated at 100 V in TGS (Tris Glycine SDS) running buffer (Bio-Rad). Separated proteins were transferred onto nitrocellulose membranes (Trans-blot Turbo Transfer Pack, Bio-Rad) using the Trans-blot turbo transfer system (Bio-Rad). As a routine, the High-MW protocol (25 V, 2.5 A, 10 min) or the Standard protocol (25 V, 1 A, 30 min) were used for the transfer. Membranes were saturated in blocking buffer (TBS, 0.1% Tween-20, 3% BSA) for 30 - 60 min at RT with gentle shaking and incubated overnight at 4°C or 1 h at RT with the primary Ab diluted (see Key resources table for a list of the Abs used in this study) in blocking buffer. After 3 washes of 10 min each with wash buffer (TBS-T), membranes were incubated with IRDye 800 CW or IRDye 680 CW (LI-COR) secondary Ab in blocking buffer for 45 min at RT in the dark. The membrane was then washed twice for 10 min with wash buffer (TBS-T) and once with wash buffer without Tween-20 in order to remove residual detergent and reduce the background during the acquisition. Near-Infrared (NIR) Western Blot Quantitative Detection was performed using the Odyssey CLx system (LI-COR) and the images were quantified using the Image Studio Lite software. The signal of each band was calculated as median - local background, intended as the signal detected in the area surrounding the band analyzed. The signal values were exported as excel files for relative quantification.

DDM TCR-CD3 stability assay

Cells (10×10^6/sample) were counted, centrifuged once at 425 x g, transferred to a 1.5 mL tube with RPMI 0% FBS and washed with cold PBS. The cell pellet was lysed with 150 µL of ice-cold lysis buffer (150 mM NaCl, 20 mM Tris, pH 8.0 containing 0.5 or the indicated % of n-Dodecyl β-D-maltoside (DDM) (Millipore), 1X protease inhibitors (cOmplete, EDTA free, Roche), 1 mM Sodium Orthovanadate (NEB), 10 mM Sodium Fluoride (Sigma) and 25 U/ml Benzonase Nuclease (Millipore) and incubated 30 min on ice. 0.5% DDM was chosen as it is the highest concentration of detergent compatible with maximal recovery of CD3 ε and ζ associated with αβ, in contrast, increasing concentration of DDM from 1% to 4% gradually decreased CD3 recovery, affecting ζ more than ε (Figure S2A and data not shown). These data agreed with the recent cryo-EM structure (Dong et al., 2019) that shows ζζ to be the dimer most loosely associated to the rest of the complex. Lysates were centrifuged 10 min at 16,100 x g at 4°C and 15 µL of the post nuclear supernatant were saved for input control. The rest of the supernatant was transferred in a new tube containing 10 µL of anti-HA-conjugated agarose beads (Sigma) pre-washed 3 times with 1 mL of lysis buffer. One sample was transferred to a tube containing anti-HA-conjugated agarose beads pre-washed and pre-saturated for 2 h at 4°C under constant rotation with 50 µg/ml of HA peptide (Sigma), to assess background. Protein complexes were pulled down by incubating the supernatant and the anti-HA-conjugated agarose beads for 1 h at 4°C under constant rotation. Beads were centrifuged for 30 s at 2,500 x g and washed 3 times with 1 mL of ice-cold lysis buffer. After the last wash, the supernatant was carefully removed and the beads re-suspended in 20 µL 1X NuPAGE LDS Sample Buffer (Invitrogen) containing or not (see below) 1X NuPAGE Sample Reducing Agent (Invitrogen) and incubated at 70°C for 10 min. After cooling, beads were centrifuged for 30 s at 2,500 x g and the supernatant was

collected in a fresh tube, loaded on a 15% polyacrylamide gel at 4°C and processed as described above (SDS-PAGE, immunoblotting and quantitation). The results were analyzed and presented as follow: for each sample, the background subtracted signals of ε, γ, δ and ζ were normalized to β and the obtained ratios (ε/β, γ/β, δ/β and ζ/β) were normalized to WT. Statistical unpaired t test analysis was performed using Prism (GraphPad Software). To evaluate γε and δε recovery to αβ, the supernatant from a single pull down was split in two, loaded in duplicates on a 15% polyacrylamide gel and immunoblotted for either β (HA), γ, ε and ζ or β (HA), δ, ε and ζ. Moreover, to minimize the interference of the IgG light chain of the anti-HA, used to pull down β, with the detection of δ and γ (molecular weight 20 - 25 kDa), at the end of the procedure described above, the beads were re-suspended in 20 μL 1X NuPAGE LDS Sample Buffer (Invitrogen) without adding reducing agent, but rather adding Iodoacetamide (Sigma) to a final concentration of 20 mM, an alkylation agent used to block thiols of proteins. Iodoacetamide was present in all the buffers used in this specific pull down (lysis buffer and sample buffer).

For endoglycosidase H (endo H) treatment, protein complexes were eluted by re-suspending the beads in 22 μL of lysis buffer containing 1X Glycoprotein Denaturing Buffer (NEB) and incubating at 100°C for 10 min. Beads were centrifuged for 30 s at 2,500 x g, the supernatant was collected, divided in two fresh tubes and 1X buffer 3 (NEB) was added in presence or not of endo H (750 U) for 1 h at 37°C. Samples were then incubated at 70°C for 10 min with 1X NuPAGE LDS Sample Buffer (Invitrogen) containing 1X NuPAGE Sample Reducing Agent (Invitrogen) and loaded on a 15% polyacrylamide gel at 4°C and processed as described above (SDS-PAGE, immunoblotting and quantitation).

ζTST pull-down

J76 $CD8^+$ 1G4 ζKO expressing inducible ζTST were treated with 1 μg/ml doxycycline overnight, counted and washed once with 150 μL of ice-cold PBS. Pellets of 10×10^6 cells were lysed with 150 μL ice-cold lysis buffer (150 mM NaCl, 20 mM Tris, pH 8.0 containing 0.5% n-Dodecyl β-D-maltoside (DDM) (Millipore), 1X proteases inhibitors (cOmplete, EDTA free, Roche), 1 mM Sodium Orthovanadate (NEB), 10 mM Sodium Fluoride (Sigma) and 25 U/ml Benzonase Nuclease (Millipore) and incubated on ice for 10 min. Lysates were centrifuged for 10 min at 16,100 x g at 4°C and 15 μL of the post nuclear supernatant was collected as a control for total input. The rest of the supernatant was transferred into a fresh tube containing 10 μL Strep-Tactin Sepharose beads (IBA Lifesciences) pre-washed with lysis buffer and incubated for 30 min at 4°C under constant rotation. Alternatively, the supernatant was incubated 30 min at 4°C with anti-HA-conjugated agarose beads (Sigma). One sample was transferred to a tube containing Strep-Tactin beads or anti-HA-conjugated agarose beads pre-washed and pre-saturated for 2 h at 4°C under constant rotation with 50 mM biotin or 50 μg/ml of HA peptide respectively, to assess background. Beads were centrifuged for 30 s at 2,500 x g and washed 3 times with 1 mL of ice-cold lysis buffer. After the last wash, the supernatant was carefully removed and the beads re-suspended in 20 μL 1X NuPAGE LDS Sample Buffer (Invitrogen) containing 1X NuPAGE Sample Reducing Agent (Invitrogen) and incubated at 70°C for 10 min. After cooling, beads were centrifuged for 30 s at 2,500 x g and the supernatant was collected in a fresh tube before being separated on a 15% polyacrylamide gel at 4°C. Quantitative immunoblotting with anti-HA and anti-ζ mAbs were used to identify the β isoform interacting with ζ among the three identified in the total β pull-down performed using anti-HA-conjugated agarose beads.

Ligand-induced TCR-CD3 quaternary structure changes

Resting or pMHC-stimulated TCR-CD3

The structural integrity of the TCR-CD3 complex upon $(9V\text{-}A2)_4$ and sm-9V-A2 stimulation of intact cells at physiological temperature or at 0°C was evaluated by the following assay. J76 $CD8^+$ or $CD8^-$ cells expressing inducible TCR wtc51 were treated with 1 μg/ml doxycycline overnight, counted and washed once with RPMI without FBS. Cells were re-suspended at 10×10^6/150 μL in the same medium and pre-incubated for 10 min at 37°C in a ThermoMixer C (Eppendorf) under constant shaking (500 rpm) or 20 min on ice. $(9V\text{-}A2)_4$ obtained using a His-tagged streptavidin (His-SA) (100 nM), sm-9V-A2 (200 nM) or RPMI alone was added to the cells for 5 min at 37°C or on ice. After ligand binding, the samples were immediately centrifuged 30 s at 800 x g and rapidly washed once with 150 μl of ice-cold PBS. For Lck inhibition, cells were pre-treated with 5 μM A770041 (Axon) at 37°C for 20 min prior to stimulation and 5 μM A770041 was kept during the binding. Cell pellets were lysed with 150 μL ice-cold lysis buffer (300 mM NaCl, 20 mM Tris, pH 8.0 containing 0.5% n-Dodecyl β-D-maltoside (DDM) (Millipore), 1X proteases inhibitors (cOmplete, EDTA free, Roche), 1 mM Sodium Orthovanadate (NEB), 10 mM Sodium Fluoride (Sigma), 25 U/ml Benzonase Nuclease (Millipore) and 10 mM Imidazole and incubated on ice for 5 min. Lysates were centrifuged for 5 min at 16,100 x g at 4°C and 15 μL of the post nuclear supernatant was collected as a control for total input. The rest of the supernatant was incubated 5 min on ice with 1 μg of His-tagged streptavidin (sm-9V-A2 engaged samples) or transferred directly into a fresh tube containing 10 μL HisPur Cobalt Resin (Thermo Fisher) ($(9V\text{-}A2)_4$ engaged samples) or 10 μL anti-HA-conjugated agarose beads (Sigma) (non-stimulated samples), pre-washed with lysis buffer and incubated for 15 min and 30 min respectively at 4°C under constant rotation. To assess background, one sample was transferred to HisPur Cobalt beads or anti-HA-conjugated agarose beads pre-washed and pre-saturated at 4°C for 2 h under constant rotation with 300 mM Imidazole or 50 μg/ml of HA peptide respectively. Beads were centrifuged for 30 s at 2,500 x g and washed 3 times with 1 mL of ice-cold lysis buffer. After the last wash, the supernatant was carefully removed and the beads re-suspended in 20 μL 1X NuPAGE LDS Sample Buffer (Invitrogen) without reducing agent and incubated at 80°C for 5 min. After cooling, beads were centrifuged for 30 s at 2,500 x g and the supernatant was collected in a fresh tube and incubated at 80°C for 5 min with 1X NuPAGE Sample Reducing Agent (Invitrogen). Samples were separated on a 15% polyacrylamide gel at 4°C and processed as described above (SDS-PAGE, immunoblotting and quantitation). Quantitative immunoblotting with anti-HA and anti-ζ mAbs were used to evaluate the amount of β and ζ

pulled-down in each condition. The amount of ζ extracted at steady state was normalized to a specific β isoform (β_2) that we proved to be the only one able to interact with the ζ chain (for details see in ζTST pull down) and that corresponds to the only detectable isoform in $(9V\text{-}A2)_4$-engaged receptor pull downs (for schematic of the procedure see Figures **S6A** and **S7A**). The value for ζ/β_2 ratio from non-stimulated samples was set equal to one. This value represented the recovery of intact TCR-CD3 complex and was compared to ζ/β_2 from $(9V\text{-}A2)_4$ stimulated samples. Statistical analysis using unpaired t test was performed using Prism (GraphPad Software).

To isolate sm-9V-A2-engaged wtc51 with monomeric avidin agarose beads (Thermo), cells were incubated with sm-9V-A2 (200 nM) or RPMI alone for 5 min at 37°C. After ligand binding, the samples were lysed as described above and the post-nuclear supernatant was incubated with monomeric avidin beads for 30 min at 4°C under constant rotation.

For primary CD8 T cells expressing constitutive TCR wtc51, cells were incubated for 5 min at 37°C with $(9V\text{-}A2)_4$ obtained using a His-tagged streptavidin (His-SA) (40 nM), 9V-A2 (400 nM) or RPMI alone. Lysis was performed in presence of 30 mM imidazole and this concentration was maintained during the following washes.

Isolation of $(6I\text{-}A2)_4$ and sm-6I-A2 engaged receptors in J76 $CD8^+$ cells expressing 868 TCR (K_d = 50 nM) was performed as described above with little variations. Cells were incubated with $(6I\text{-}A2)_4$ obtained using a His-tagged streptavidin (His-SA) (40 nM), sm-6I-A2 (400 nM) or RPMI alone for 5 min at 37°C. Lysis was performed in the presence of 30 mM imidazole and this concentration was maintained during the following washes.

To have a comparable amount of β detected by immunoblotting in non-stimulated samples and $(6I\text{-}A2)_4$ and sm-6I-A2 engaged samples, only 1/3 of the elution from non-stimulated samples was loaded on the gel.

Isolation of $(9V\text{-}A2)_4$ engaged receptors in J76 $CD8^+$ cells expressing inducible QM-α TCR (K_d = 140 nM) was performed as described above with little variations. Cells were incubated with $(9V\text{-}A2)_4$ obtained using a His-tagged streptavidin (His-SA) (333 nM), or RPMI alone for 5 min at 37°C. Lysis was performed in the presence of 30 mM imidazole and this concentration was maintained during the following washes. Elution was performed by incubating the beads at 4°C for 15 min with 15 μL of lysis buffer containing 300 mM imidazole. Beads were then centrifuged for 30 s at 2500 x g and the supernatant was collected in a fresh tube and incubated at 80°C for 5 min with 1X NuPAGE LDS Sample Buffer containing 1X NuPAGE Sample Reducing Agent (Invitrogen).

To have a comparable amount of β detected by immunoblotting in non-stimulated samples and $(9V\text{-}A2)_4$ engaged samples, only 1/40 of the elution from non-stimulated samples was loaded on the gel.

Soluble monovalent-pMHC stimulation

Intracellular triggering upon soluble monovalent agonist (sm-pMHC) binding was evaluated by pErk activation, according to the following procedure. J76 $CD8^+$ or $CD8^-$ cells expressing 868 or wtc51 TCRs (the latter induced for TCR-CD3 expression with 1 μg/ml doxycycline for 48 h) were counted and washed once with RPMI without FBS. Cells were re-suspended at 10×10^6/125 μL in the same medium and pre-incubated for 10 min at 37°C in a ThermoMixer C (Eppendorf) under constant shaking (500 rpm). For Lck inhibition, cells were pre-treated with 5 μM A770041 (Axon) at 37°C for 15 min prior to stimulation and 5 μM A770041 was kept during the binding. Tetramers ($pMHC_4$,25 nM), sm-pMHC (100 nM) or RPMI alone were added to the cells for 5 min at 37°C. After ligand binding, samples were immediately boiled for 10 min at 95°C by adding pre-warmed 2X NuPAGE LDS Sample Buffer (Invitrogen) containing 2X NuPAGE Sample Reducing Agent (Invitrogen) to instantly stop the reaction. Cell lysates were let cool down and 25 U of Benzonase Nuclease (Millipore) were added every 15 min for 4 times to allow a complete DNA/RNA digestion. Lysates were centrifuged for 15 min at 16,100 x g at 4°C and supernatant was collected in a fresh tube before being separated on a 15% polyacrylamide gel. Quantitative immunobloting with anti-pErk and anti-actin was used to evaluate the amount of Erk phosphorylation (for details, see SDS-PAGE, immunoblotting and quantitation). The value for pErk (-background)/actin ratio from non-stimulated samples was set equal to one. Statistical analysis using unpaired t test was performed using Prism (GraphPad Software).

To exclude that the observed signaling (pErk) was the consequence of surface cell-to-cell ligand cross-presentation, we used the above protocol (also used for the evaluation of sm-pMHC binding by FACS, see sm-pMHC binding to cells section) with cells expressing or not TCR. Briefly, CD8-deficient wtc51 J76 not induced for TCR expression were counted, washed once with RPMI, re-suspended at 10×10^6/125 μL in the same medium and pre-incubated for 10 min at 37°C in a ThermoMixer C (Eppendorf) under constant shaking (500 rpm). Sm-9V-A2 (200 nM) or RPMI alone was added to the cells for 5 min at 37°C, rapidly washed with ice-cold RPMI (to minimize unbinding), re-suspended in pre-warmed RPMI at 10×10^6/125 μL and added to an equal amount of dox-induced CD8-deficient wtc51 J76 for 5 min at 37°C. After ligand binding cells were immediately boiled for 10 min at 95°C by adding pre-warmed 2X NuPAGE LDS Sample Buffer (Invitrogen) containing 2X NuPAGE Sample Reducing Agent (Invitrogen) to instantly stop the reaction and cell lysates were analised as described above.

UCHT1-Fab' or UCHT1 Ab-engaged TCR-CD3

The structural integrity of the TCR-CD3 complex upon agonist stimulation of intact cells at physiological temperature or at 0°C was evaluated by the following assay. The anti-CD3ε mAb UCHT1 was used as a potent agonist of TCR-CD3 and its Fab' as the non-agonist control that binds to the same determinant. J76 $CD8^+$ cells stably expressing 1G4 TCR were counted and washed once with RPMI without FBS. Cells were re-suspended at 10×10^6/150 μL in the same medium and pre-incubated for 10 min at 37°C in a ThermoMixer C (Eppendorf) under constant shaking (500 rpm) or for 20 min on ice. Mono-biotinylated Fab' of UCHT1 mAb (0.72 μg) or biotinylated UCHT1 intact mAb (2 μg) (BioLegend) was added to the cells for 5 min at 37°C or on ice. These doses

of anti-CD3ε ligands that corresponded to approximately the same molarity were proven to pull-down similar amounts of TCR-CD3 complex, as detected by ε immunoblot. One sample was left untreated as negative control. After ligand binding, the samples were immediately centrifuged 30 s at 800 x g and rapidly washed once with 150 μL of ice-cold PBS. For Lck inhibition, cells were pre-treated with 5 μM A770041 (Axon) at 37°C for 20 min prior to binding of Fab' UCHT1 or UCHT1 mAb and 5 μM A770041 was kept during the binding. Cell pellets were lysed with 150 μL ice-cold lysis buffer (150 mM NaCl, 20 mM Tris, pH 8.0 containing 0.5% n-Dodecyl β-D-maltoside (DDM) (Millipore), 1X proteases inhibitors (cOmplete, EDTA free, Roche), 1 mM Sodium Orthovanadate (NEB), 10 mM Sodium Fluoride (Sigma) and 25 U/ml Benzonase Nuclease (Millipore) and incubated on ice for 30 min. Lysates were centrifuged for 10 min at 16,100 x g at 4°C and 15 μL of the post nuclear supernatant was collected as a control for total input. The rest of the supernatant was transferred into a fresh tube containing 10 μL Streptavidin Sepharose High Performance beads (GE Healthcare) pre-washed with lysis buffer and incubated for 1 h at 4°C under constant rotation. Beads were centrifuged for 30 s at 2,500 x g and washed 3 times with 1 mL of ice-cold lysis buffer. After the last wash, the supernatant was carefully removed and the beads re-suspended in 20 μL 1X NuPAGE LDS Sample Buffer (Invitrogen) containing 1X NuPAGE Sample Reducing Agent (Invitrogen) and incubated at 70°C for 10 min. After cooling, beads were centrifuged for 30 s at 2,500 x g and the supernatant was collected in a fresh tube before being separated on a 15% polyacrylamide gel. Quantitative immunoblotting with anti-ε, anti-HA and anti-ζ mAbs was used to evaluate the amount of ε, β and ζ, respectively, pulled-down after each stimulatory condition (for details, see SDS-PAGE, immunoblotting and quantitation). The recovery of ε was assumed to be invariant in both the stimulatory (UCHT1 mAb) and non-stimulatory (UCHT1-Fab') condition and was used for the normalization of the amounts of the other subunits in each pull-down. Therefore, the values for β/ε and ζ/ε ratios obtained from UCHT1-Fab' engaged TCR-CD3 were set equal to one. These values represented the recovery of intact TCR-CD3 complex and were compared to the same ratios from UCHT1 stimulated samples. Statistical analysis using unpaired t test was performed using Prism (GraphPad Software).

Microscopy

dSTORM imaging and analysis

Prior to dSTORM imaging, cells were plated on glass μ-slide 8-wells chamber (Ibidi) coated with recombinant human ICAM-1 protein (R&D Systems) at 2.5 μg/ml concentration. Cells were incubated for 15 minutes at 37°C followed by fixation for 30 min with 4% PFA at RT. Cells were blocked with 5% BSA in PBS for 1 h followed by incubation with an anti-CD3 primary antibody directly conjugated with Alexa Fluor 647 (BioLegend) for 1 h at RT. Cells were washed three times with PBS and post-fixed with 4% PFA for 5 min before imaging. Cysteamine based dSTORM imaging buffer was used to perform the single molecule localization experiments with the following composition: 100 mM Cysteamine MEA (Sigma Aldrich), 5% Glucose (Sigma Aldrich), 1% Glox (0.5 mg/mL glucose oxidase, 40 mg/mL catalase (Sigma Aldrich) dissolved in 1X PBS. dSTORM imaging of CD3 was performed by using a 150 × 1.45-NA oil-immersion objective in TIRF mode (TIRF; Olympus). Laser light of 642 nm was used to excite the Alexa Fluor 647 dye and switch it to the dark state. An additional 405 nm laser light was used to reactivate the Alexa Fluor 647 fluorescence. The emitted light from Alexa Fluor 647 was collected by the same objective and imaged by an EMCCD camera (Evolve Delta; Photometrics) at a frame rate of 10 ms per frame. A maximum of 5,000 frames per condition were acquired. dSTORM images were analyzed and rendered as previously described (Bates et al., 2007; Huang et al., 2008) using a custom-written software (Insight3, provided by B. Huang, University of California, San Francisco). Peaks in single-molecule images were identified based on a threshold and fit to a simple Gaussian to determine the x and y positions. Only localizations with photon count > 400 photons were included, and localizations that appeared within one pixel in five consecutive frames were merged together and fitted as one localization. The final images were rendered by representing the x and y positions of the localizations as a Gaussian with a width that corresponds to the determined localization precision. Sample drift during acquisition was calculated and subtracted by reconstructing dSTORM images from subsets of frames (500 frames) and correlating these images to a reference frame (the initial time segment).

Pair auto-correlation analysis

Pair auto-correlation analysis is independent of the number of localizations and is not susceptible to over-counting artifacts related to fluorescent dye re-blinking (Stone et al., 2017). Auto-correlation analysis of CD3 protein was performed using MATLAB software provided by Sarah Shelby and Sarah Veatch from University of Michigan. Regions containing cells were masked by a region of interest, and the auto-correlation function from the x and y coordinate list from the 642 nm dSTORM channel was computed from these regions using an algorithm described previously (Shelby et al., 2013; Stone et al., 2017; Veatch et al., 2012).

DBSCAN cluster analysis

Quantitative cluster analysis was based on Density-based spatial clustering of applications with noise (DBSCAN) (Ester et al., 1996). The DBSCAN method detects clusters using a propagative method which links proteins belonging to the same cluster based on the minimum number of neighbors ε (ε = 7) in the radius r (r = 25 nm). Clus-DoC (Pageon et al., 2016) MATLAB based software with implemented DBSCAN analysis was used. The x and y coordinate list of dSTORM localizations was used and regions containing cells were selected to give the mean ± SD value of CD3 cluster size per cell.

Fluorescent Recovery after Photobleaching (FRAP) and analysis

Prior to FRAP experiment, cells were incubated for 20 minutes at 37°C with an anti-CD3 primary antibody directly conjugated with Alexa Fluor 647 (BioLegend). Cells were washed with cell media and seeded on glass μ-slide 8 well chamber (Ibidi) coated with Poly-L-Lysine for 5 min before imaging. Cysteamine based dSTORM imaging buffer was used to perform the

experiments (see dSTORM imaging and analysis). FRAP imaging of CD3 was performed by using a Nikon A1R HD25 confocal system with a 60x oil-immersion objective (Nikon, UK) in humidified 37°C, 5% CO_2 chamber. A circular region was defined on the surface of the cell. This region was bleached using the 405 nm laser with maximum power for 7 s. Before and after bleaching, the whole region was visualized by 640 nm laser for the duration of 50 s in 4 s intervals. FRAP curves were exported from NIS-Elements software (Nikon, UK) and the post processing of the extracted curves was performed in Origin software (OriginPro 2017; OriginLab).

Atomistic Molecular Dynamics Simulations

The transmembrane region (TMR) of the T cell receptor (TCR) was extracted from the cryo-EM structure PDB: 6JXR (Dong et al., 2019) and used for atomistic molecular dynamics simulations. The residues of TCRβ TMR are numbered according to that used in our experiments ("n - 1" compared to PDB: 6JXR, i.e., our βY291 is βY292 in 6JXR). The TMR sequences used are shown in Table S3. The octameric protein structure was inserted in a complex asymmetric bilayer (lipid concentration is shown in Table S4) using CHARMM-GUI (https://charmm-gui.org) and was solvated with TIP3 water molecules. The lipid types and their concentrations in our simulated bilayer were considered from studies on the T cell membrane conducted by Zech et al. (2009). The system was then neutralized with a concentration of 150 mM of NaCl ions. The resultant molecular system and molecular dynamics parameters were obtained from CHARMM-GUI and thereafter run using Gromacs 2016.4 (Van Der Spoel et al., 2005) using the CHARMM36 forcefield (Lee et al., 2016). An energy-minimization of the entire system was conducted for 5000 steps using the steepest descent algorithm. The energy-minimized system underwent a 6-step NPT equilibration where position restraints on the protein backbone, side-chain and lipids were gradually released. The LINCS algorithm (Hess et al., 1997) was used to apply constraints on bond lengths. The fully equilibrated system was used to generate starting points for 3 production simulation repeats with different initial velocities. This was done for the wild-type complex, ζA38, ζA41 and βA291 mutants which started from the same initial configuration. Each production simulation was run for 1250 ns employing a 2 femtoseconds time-step and the co-ordinates were saved every 40 pico-seconds. The Nose-Hoover thermostat (Hoover, 1985) was used with a reference temperature of 323 K and coupling constant of 1 picosecond. The Parrinello-Rahman barostat (Parrinello and Rahman, 1981) was used with a semi-isotropic pressure coupling type, reference pressure of 1 atmosphere, compressibility value of 4.5×10^{-5} bar^{-1} and coupling constant of 5 picoseconds. The Particle mesh Ewald algorithm (Essmann et al., 1995) with a 12 Å distance cut-off was used to define non-bonded van der Waals (Lennard-Jones) and coulombic interactions. Visualization was carried out using VMD (Humphrey et al., 1996). Protein-protein interaction profiles and spatial distribution plots are a result of merged data from all the 3 repeats of all-atom simulations. To calculate the spatial distribution of ζζ relative to αβ, the positions of Cα atoms of αβ were fixed throughout the simulation time. The spatial distributions of Cα atoms of ζζ and αβ are viewed from the extracellular region.

Modeling of the 1G4 TCR affinity mutants

The 1G4-A2-SLL structure (from PDB: 2BNR) (Chen et al., 2005) was used to model the 1G4 QM-α-A2-SLL and 1G4 wtc51-A2-SLL tri-molecular complex structures. Sequences were adjusted with COOT (Emsley and Cowtan, 2004) and graphical representations and analysis were prepared with PYMOL (DeLano, 2002; Available: https://pymol.org/2/).

QUANTIFICATION AND STATISTICAL ANALYSIS

Flow cytometry

All experiments were replicated at least two - three times with technical and/ or biological replicates each time. Raw data was processed in FlowJo (FlowJo software, part of BD) and MFIs, counts or percentages were extracted from FlowJo as excel files. Statistical analysis and non-linear regression fitting were performed with Prism (GraphPad Software). Significance was assessed by two-tailed paired or unpaired t test. Non-linear regressions were tested for Goodness of Fit (R^2), for normality (D'Agostino & Pearson omnibus normality test) and with the replicates test. Non-linear regression fits are specified in the methods paragraph describing the experimental procedure. R^2 and *p-value* for the fits, significance tests and significance are indicated in the figure or figure legend.

Pull-down and Immunoblotting

Experiments were replicated at least two - three times with technical and/ or biological replicates each time as indicated at the representing figure. Raw data was extracted from Image studio (LI-COR Biosciences) and processed in Excel and Prism (GraphPad Software). Statistical analysis (two-tailed unpaired t test) was performed and method and significance are indicated in the figure or figure legend.

Microscopy

Samples were tested for normality with a Kolmogorov–Smirnov test. The statistical significance of differences between two datasets was assessed by a two-tailed t test assuming unequal variance. All statistical analysis was performed using Origin software (OriginPro 2017; OriginLab).

Simulations

For the atomistic simulations multiple repeat simulations were performed to ensure reproducibility of our results. Analyses of the simulations were conducted using Gromacs, VMD, and locally written code.

Modeling

Graphics and analysis were prepared with PYMOL (DeLano, 2002; Available: https://pymol.org/2/).

Significance

* $p \leq 0.05$ /** $p \leq 0.01$/*** $p \leq 0.001$/**** $p \leq 0.0001$/ ns = not significant.

Cell Reports, Volume *36*

Supplemental information

Allosteric activation of T cell antigen receptor signaling by quaternary structure relaxation

Anna-Lisa Lanz, Giulia Masi, Nicla Porciello, André Cohnen, Deborah Cipria, Dheeraj Prakaash, Štefan Bálint, Roberto Raggiaschi, Donatella Galgano, David K. Cole, Marco Lepore, Omer Dushek, Michael L. Dustin, Mark S.P. Sansom, Antreas C. Kalli, and Oreste Acuto

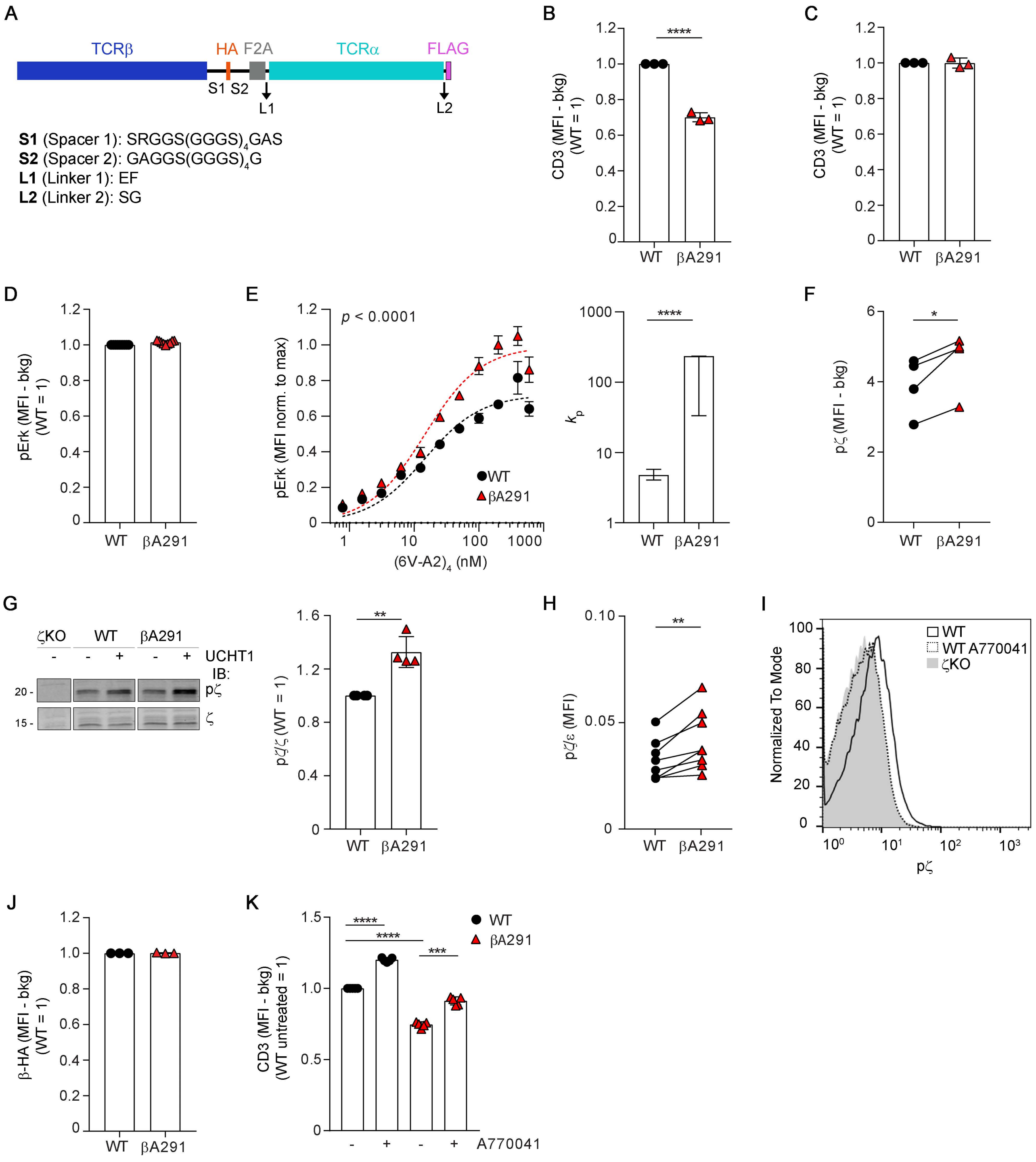
A
TCRβ
HA
F2A
TCRα
FLAG
S1 S2
L1
L2
S1 (Spacer 1): SRGGS(GGGS)4GAS
S2 (Spacer 2): GAGGS(GGGS)4G
L1 (Linker 1): EF
L2 (Linker 2): SG
B
C
CD3 (MFI - bkg) (WT = 1)
WT
βA291
****
D
pErk (MFI - bkg) (WT = 1)
E
p < 0.0001
pErk (MFI norm. to max)
(6V-A2)4 (nM)
kp
F
pζ (MFI - bkg)
G
ζKO
WT
βA291
UCHT1
IB:
pζ
ζ
pζ/ζ (WT = 1)
H
pζ/ε (MFI)
I
WT
WT A770041
ζKO
Normalized To Mode
pζ
J
β-HA (MFI - bkg) (WT = 1)
K
CD3 (MFI - bkg) (WT untreated = 1)
A770041

Fig S1

Figure S1. Gain-of-function mutations in the βTMR, related to Figure 1

A Scheme of the single polypeptide self-splicing 1G4 αβ expressed in J76 and $CD8^-$ J76 cells. β chain (blue), α chain (cyan), HA-tag (orange), FLAG-tag (magenta), F2A sequence (grey). The entire aa sequence can be found in **Table S1**. Spacer and short linker aa sequences are indicated below the scheme. **B** CD3 surface expression of $CD8^-$ J76 1G4-WT or 1G4-βA291. Cells were labelled or not with CellTrace violet, mixed 1:1 and analysed for CD3 surface expression and total TCRβ-HA by FACS. Gates for equal HA-tag expression level were applied and the CD3 MFI of 1G4-WT and 1G4-βA291 was extracted from the TCRβ-HA^{low} gate, see STAR Methods. Mean ± SEM of background subtracted CD3 MFI from the indicated gate normalised to the corresponding WT in the same gate, n = 3 experiments in duplicates, unpaired *t*-test $p < 0.0001$. **C** Similar CD3 surface expression of $CD8^-$ J76 1G4-WT and 1G4-βA291. Cells were induced with different doses of doxycycline, labelled or not with CellTrace violet, mixed 1:1 and analysed for CD3 surface expression by FACS, see STAR Methods. Mean ± SD of $CD3^+$ MFI normalised to WT, n = 3 experiments in triplicates, unpaired *t*-test (ns). **D** PMA/Iono induced pErk response of $CD8^-$ J76 1G4-WT or 1G4-βA291. Cells were labelled or not with CellTrace violet, mixed 1:1, treated with PMA/Iono (5') and analysed for pErk by FACS. Mean ± SD of pErk MFI normalised to WT, n = 3 experiments in triplicates, unpaired *t*-test (ns). **E** pErk response of $CD8^-$ J76 1G4-WT or 1G4-βA291 stimulated with $(6V\text{-}A2)_4$. Cells were induced with different doses of doxycycline, labelled or not with CellTrace violet, mixed 1:1 and stimulated for 3 min with different doses (0.78 – 600 nM) of PE-conjugated $(6V\text{-}A2)_4$ and analysed for pErk and $(6V\text{-}A2)_4$ binding by FACS. **Left**, $(6V\text{-}A2)_4$ nM vs. pErk MFI normalised to max (showed in Fig. **1C**) fitted to a minimal model of kinetic proofreading, n = 3 experiments in triplicates, $R^2 = 0.84$ (WT), 0.90 (βA291), F-test $p < 0.0001$. **Right**, mean ± SD of proofreading rate (k_p), n = 3 experiments in triplicates, F-test $p < 0.0001$. **F** Paired max. pζ values related to dose response reported in Fig. **1E**. J76 1G4-WT or 1G4-βA291 were labelled or not with CellTrace violet, mixed 1:1, stimulated for 1 min with increasing doses (3.125 - 200 nM) of PE-conjugated $(6V\text{-}A2)_4$ and analysed for pζ and $(6V\text{-}A2)_4$ binding by FACS. Background subtracted MFI of $(6V\text{-}A2)_4$ was plotted vs. pζ MFI and fitted by non-linear regression, n = 4, ratio paired *t*-test $p = 0.023$. **G** pζ response of J76 1G4-WT or 1G4-βA291 stimulated or not with UCHT1 Ab. **Left**, immunoblot (IB) representative of 4 experiments. **Right**, mean ± SD of pζ/ζ, n = 4, unpaired *t*-test $p = 0.0013$. **H** Paired mean values of the basal pζ in J76 1G4-WT or 1G4-βA291 reported in Fig. **1F**. Resting cells were analysed for pζ or CD3 surface expression by FACS. pζ MFI was normalised to surface CD3 MFI, n = 8, paired *t*-test $p = 0.0037$. **I** Representative FACS histograms of pζ staining related to Fig **1F**. J76 1G4-WT cells were treated or not with A770041 (5 μM) for 15 min at 37 ºC and analysed for pζ by FACS. J76-1G4WT-ζKO served as a negative control. **J** β-HA total expression of $CD8^-$ J76 1G4-WT or 1G4-βA291. Cells were labelled or not with CellTrace violet, mixed 1:1 and analysed for CD3 surface expression and total TCRβ-HA by FACS in a single HA bin, see STAR Methods. Mean ± SEM of background subtracted HA MFI in HA^{low} gate, n = 3, unpaired *t*-test (ns). **K** TCR-CD3 surface expression in J76 1G4-WT or 1G4-βA291 treated or not with A770041, n = 5, unpaired *t*-test $p < 0.0001$ (βA291 vs. WT), $p < 0.0001$ (WT ± A770041), $p = 0.0003$ (βA291 ± A770041).

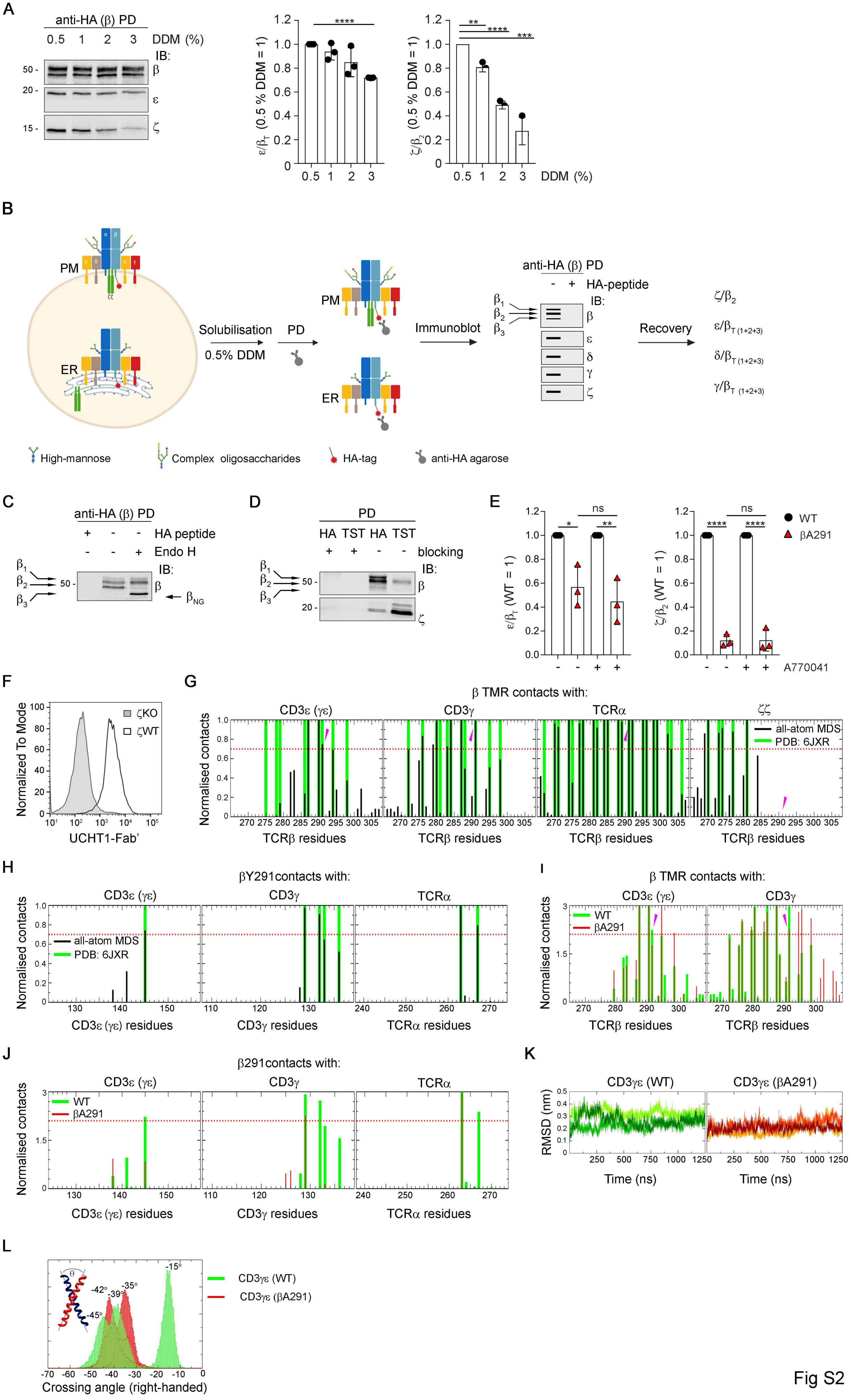
A
anti-HA (β) PD
0.5 1 2 3 DDM (%)
IB:
β
ε
ζ
ε/βT (0.5 % DDM = 1)
ζ/β2 (0.5 % DDM = 1)
DDM (%)
B
PM
ER
Solubilisation
0.5% DDM
PD
Immunoblot
anti-HA (β) PD
HA-peptide
Recovery
ζ/β2
ε/βT (1+2+3)
δ/βT (1+2+3)
γ/βT (1+2+3)
High-mannose
Complex oligosaccharides
HA-tag
anti-HA agarose
C
anti-HA (β) PD
HA peptide
Endo H
βNG
D
PD
HA TST HA TST
blocking
E
ε/βT (WT = 1)
ζ/β2 (WT = 1)
WT
βA291
A770041
F
Normalized To Mode
ζKO
ζWT
UCHT1-Fab'
G
β TMR contacts with:
CD3ε (γε)
CD3γ
TCRα
ζζ
all-atom MDS
PDB: 6JXR
Normalised contacts
TCRβ residues
H
βY291contacts with:
CD3ε (γε)
CD3γ
TCRα
all-atom MDS
PDB: 6JXR
CD3ε (γε) residues
CD3γ residues
TCRα residues
I
β TMR contacts with:
WT
βA291
TCRβ residues
J
β291contacts with:
WT
βA291
K
CD3γε (WT)
CD3γε (βA291)
RMSD (nm)
Time (ns)
L
CD3γε (WT)
CD3γε (βA291)
Crossing angle (right-handed)

Fig S2

Figure S2. βY291 contribution to TCR-CD3 quaternary structure cohesion, related to Figure 2

A Sensitivity of TCR-CD3 quaternary structure cohesion to DDM concentrations. $CD8^-$ J76 1G4-WT were solubilised with increasing concentrations of DDM and analysed by β-HA PD and IB for β, ε and ζ (**left**). Mean ± SD of ε/β_T (**middle**) and ζ/β_2 (**right**), n = 3, unpaired *t*-test (ε/β_T): $p < 0.0001$; (ζ/β_2): 0.5 vs. 1 % $p < 0.01$, 0.5 vs. 2 % $p < 0.0001$, 0.5 vs. 3 % $p < 0.001$. Note that at ≤ 0.5 % DDM there is no detectable change in cohesion of CD3 subunits with TCRαβ, further supporting that in TCR-CD3 extracted at 0.5 % DDM the subunits' stoichiometry in the octamer remains intact (Swamy et al., 2008). At ≥ 1 % DDM there is progressive loss of ζ followed by γε and δε. **B** Graphic scheme describing the DSA. The entire pool of the TCRαβ is captured by anti-HA (β-HA) PD which includes partial αβγεδε complex assembled in the endoplasmic reticulum (ER) (Alcover et al., 2018), αβγεδεζζ complex resident in the *trans*-Golgi (Alcover et al., 2018) or recycling at steady state in the ER (Alcover et al., 2018) and the largest fraction of αβγεδεζζ present at the plasma membrane (PM). The IB scheme on the right shows expected band patterns for β, ε, δ, γ and ζ derived from the ER and PM. Arrows indicate the different isoforms of TCRβ (β_1, β_2, β_3). To evaluate ζ recovery, ζ/β_2 ratio was calculated and the value for ζ/β_2 ratio from WT was set equal to one. To evaluate ε, δ, γ recovery, ε/β_T, δ/β_T and γ/β_T ratios were calculated and the ratios for WT were set equal to one. These represented the recovery of intact TCR-CD3 complex. Ratios < 1 indicate a lower recovery of ζ, ε, δ and γ hence a reduced cohesion of TCR-CD3 quaternary structure. See STAR Methods for a detailed description of the experimental procedure. **C** J76 wtc51 treated or not with endo H and analysed by β-HA PD and IB for β. Arrows indicate β isoforms, β_{NG} indicates non-glycosylated β isoform after removal of high-mannose carbohydrates by endo-H treatment. Data representative of 2 experiments. **D** J76-1G4-ζKO expressing inducible ζTST were solubilised and analysed by β-HA (lanes 1, 3) or ζTST (lanes 2, 4) PD and IB for β and ζ. Arrows indicate β isoforms. IB representative of 2 experiments. **E** $CD8^-$ J76 1G4-WT or 1G4-βA291 ± A770041 were solubilised and analysed by β-HA PD and IB for β, ε and ζ. Mean ± SD of ε/β_T (**left**) and ζ/β_2 (**right**), n = 3, unpaired *t*-test (ε/β_T): 1G4-WT vs. 1G4-βA291 $p < 0.05$, 1G4-WT+A770041 vs. 1G4-βA291+A770041 $p < 0.01$; (ζ/β_2): 1G4-WT vs. 1G4-βA291 $p < 0.0001$, 1G4-WT+A770041 vs. 1G4-βA291+A770041 $p < 0.0001$; ns = non-significant. **F** Representative FACS histograms of UCHT1-Fab' staining of J76-1G4WT-ζKO reconstituted with doxycycline inducible ζWT. Cells were induced or not for ζWT expression, labelled or not with CellTrace violet, mixed 1:1 and analysed for TCR-CD3 surface expression by FACS. **G** Normalised number of contacts of the WT β TMR with the rest of the TCR-CD3 TMRs in our all-atom molecular dynamics simulations (MDS) (related to Fig. **2D**). The contacts in the cryo-EM structure (PDB: 6JXR) are shown in green for comparison with the WT all-atom MDS (black). Magenta arrow indicates β291. Normalisation was done by dividing the number of contacts of each residue by the highest number of contacts. For all contacts analyses in Figs. **S2G** - **S2J**, a cut-off distance of 4 Å was used to define a contact and the red dotted line represents 70 % of the normalised contacts, a threshold used to measure the significance of contacts. **H** Normalised number of contacts of βY291 with the rest of the TCR-CD3 TMRs (related to Fig. **2D**). Comparison of protein-protein interactions between our WT all-atom MDS (black) and the cryo-EM structure (PDB: 6JXR) (green). Normalisation was done by dividing the number of contacts of each residue by the highest number of contacts. **I** Normalised number of contacts of βWT (green) and βA291 (red) with CD3γε TMR in our all-atom MDS. Magenta arrow indicates β291. Normalisation

was done by dividing the number of contacts of each residue by the number of simulation frames. **J** Normalised number of contacts of βY291 WT (green) and βA291 mutant (red) in our all-atom MDS. Normalisation was done by dividing the number of contacts of each residue by the number of simulation frames. **K** Root mean square deviations (RMSD) of the Cα atoms of CD3γε relative to their initial configuration during the WT and βA291 all-atom MDS. Each line represents RMSD obtained from one simulation ($n = 3$). **L** Comparison of crossing angle distribution of CD3γε TMR between WT and βA291, in 3 simulations. The most observed crossing angle values are labelled.

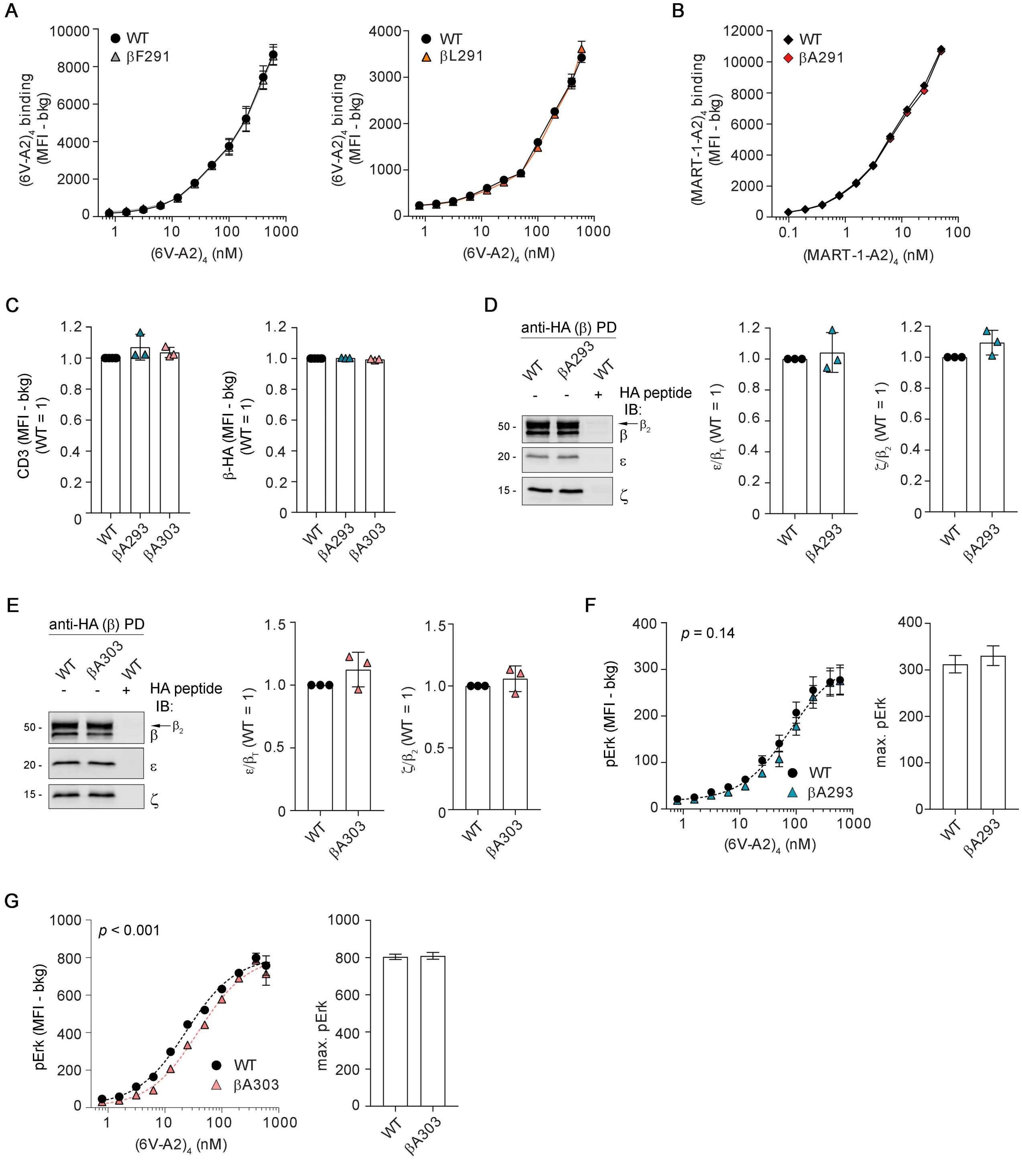

A
WT
βF291
(6V-A2)4 binding (MFI - bkg)
(6V-A2)4 (nM)
WT
βL291
B
WT
βA291
(MART-1-A2)4 binding (MFI - bkg)
(MART-1-A2)4 (nM)
C
CD3 (MFI - bkg) (WT = 1)
β-HA (MFI - bkg) (WT = 1)
WT
βA293
βA303
D
anti-HA (β) PD
HA peptide
IB:
β2
β
ε
ζ
ε/βT (WT = 1)
ζ/β2 (WT = 1)
E
anti-HA (β) PD
F
p = 0.14
pErk (MFI - bkg)
max. pErk
G
p < 0.001

Fig S3

Figure S3. Loosening ζ association enhances signalling, related to Figure 3

A $(6V\text{-}A2)_4$ binding to $CD8^-$ J76 1G4-WT or 1G4-βF291 (**left**) or 1G4-βL291 (**right**) related to Figs. **3C** and **3D**. Cells were induced with different doses of doxycycline, labelled or not with CellTrace violet, mixed 1:1 and stimulated for 3 min with different doses (0.78 – 600 nM) of PE-conjugated $(6V\text{-}A2)_4$ and analysed by FACS. Plots show mean ± SD of 3 experiments measured in triplicates. **B** $(MART\text{-}1\text{-}A2)_4$ binding to $CD8^-$ J76 2H5-WT or 2H5-βA291 related to Fig. **3G**. Cells were induced with different doses of doxycycline, labelled or not with CellTrace violet, mixed 1:1 and stimulated for 3 min with different doses (0.05 – 50 nM) of PE-conjugated $(MART\text{-}1\text{-}A2)_4$ and analysed by FACS. Plot shows mean ± SD of 3 experiments measured in triplicates. **C** TCR-CD3 surface expression in $CD8^-$ J76 of 1G4-WT, 1G4-βA293 or 1G4-βA303. Cells were labelled or not with CellTrace violet, mixed 1:1 and analysed for CD3 surface expression and total TCRβ-HA by FACS. Gates for equal HA-tag expression level were applied and the CD3 and β-HA MFI were extracted from the TCRβ-HA^{low} gate, see STAR Methods. **Left**, mean ± SEM of CD3 MFI in HA^{low} gate, 3 experiments measured in duplicates, *t*-test (ns). **Right**, mean ± SEM of β-HA MFI in HA^{low} gate, 3 experiments measured in duplicates, *t*-test (ns). **D** $CD8^-$ J76 1G4-WT or 1G4-βA293 were solubilised and analysed by β-HA PD and IB for β, ε and ζ. **Left**, IB: 1 of 3 experiments. The arrow indicates β_2 isoform. **Middle**, mean ± SD of ε/β_T, n = 3, *t*-test (ns). **Right**, mean ± SD of ζ/β_2, n = 3, unpaired *t*-test (ns). **E** $CD8^-$ J76 1G4-WT or 1G4-βA303 were solubilised and analysed by β-HA PD and IB for β, ε and ζ. **Left**, IB: 1 of 3 experiments. The arrow indicates β_2 isoform. **Middle**, mean ± SD of ε/β_T, n = 3, *t*-test (ns). **Right**, mean ± SD of ζ/β_2, n = 3, unpaired *t*-test (ns). **F** pErk response of $CD8^-$ J76 1G4-WT or 1G4-βA293. Cells were induced with different doses of doxycycline, labelled or not with CellTrace violet, mixed 1:1 and stimulated for 3 min with different doses (0.78 – 600 nM) of PE-conjugated $(6V\text{-}A2)_4$ and analysed by FACS. **Left**, non-linear regression fit of $(6V\text{-}A2)_4$ nM vs. pErk MFI, n = 3, $R^2 = 0.52$ (WT), 0.60 (βA293). **Right**, mean ± SD of max. pErk, n = 3, F-test (ns). **G** pErk response of $CD8^-$ J76 1G4-WT or 1G4-βA303. Cells were induced with different doses of doxycycline, labelled or not with CellTrace violet, mixed 1:1 and stimulated for 3 min with different doses (0.78 – 600 nM) of PE-conjugated $(6V\text{-}A2)_4$ and analysed by FACS. **Left**, non-linear regression fit of $(6V\text{-}A2)_4$ nM vs. pErk MFI, n = 3, $R^2 = 0.96$ (WT), 0.95 (βA293). **Right**, mean ± SD of max. pErk, n = 3, F-test (ns).

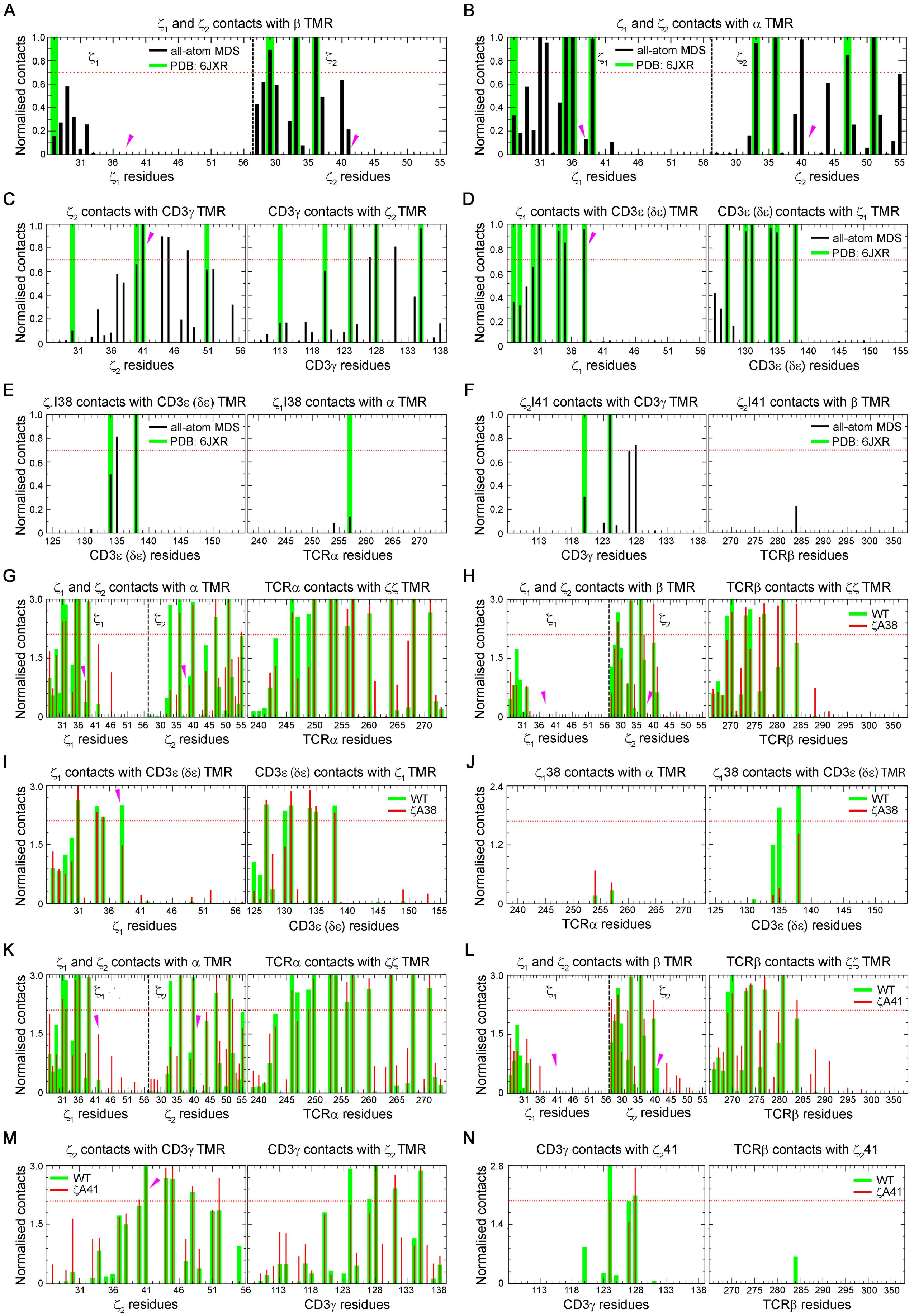

A
ζ_1 and ζ_2 contacts with β TMR
all-atom MDS
PDB: 6JXR
Normalised contacts
ζ_1 residues
ζ_2 residues
B
ζ_1 and ζ_2 contacts with α TMR
C
ζ_2 contacts with CD3γ TMR
CD3γ contacts with ζ_2 TMR
CD3γ residues
D
ζ_1 contacts with CD3ε (δε) TMR
CD3ε (δε) contacts with ζ_1 TMR
CD3ε (δε) residues
E
ζ_1I38 contacts with CD3ε (δε) TMR
ζ_1I38 contacts with α TMR
TCRα residues
F
ζ_2I41 contacts with CD3γ TMR
ζ_2I41 contacts with β TMR
TCRβ residues
G
ζ_1 and ζ_2 contacts with α TMR
TCRα contacts with ζζ TMR
H
ζ_1 and ζ_2 contacts with β TMR
TCRβ contacts with ζζ TMR
WT
ζA38
I
ζ_1 contacts with CD3ε (δε) TMR
CD3ε (δε) contacts with ζ_1 TMR
J
$\zeta_1$38 contacts with α TMR
$\zeta_1$38 contacts with CD3ε (δε) TMR
K
ζ_1 and ζ_2 contacts with α TMR
TCRα contacts with ζζ TMR
L
ζ_1 and ζ_2 contacts with β TMR
TCRβ contacts with ζζ TMR
ζA41
M
ζ_2 contacts with CD3γ TMR
CD3γ contacts with ζ_2 TMR
N
CD3γ contacts with $\zeta_2$41
TCRβ contacts with $\zeta_2$41

Fig S4

Figure S4. Loosening ζ association enhances signalling, related to Figure 4

A Normalised number of contacts of ζ_1 and ζ_2 with β TMR, in our WT all-atom MDS (black) and in the cryo-EM structure (PDB: 6JXR) (green), related to Fig. **4A**. Magenta arrow indicates ζ_1I38 (**left**) or ζ_2I41 (**right**). In Figs. **S4A** - **S4F**, when comparing the contacts in the cryo-EM structure to our WT all-atom MDS, normalisation was done by dividing the number of contacts of each residue by the highest number of contacts. For all contacts analyses in Figs. **S4A** - **S4N**, a cut-off distance of 4 Å was used to define a contact and the red dotted line represents 70 % of the normalised contacts, a threshold used to measure the significance of contacts. **B** Normalised number of contacts of ζ_1 and ζ_2 with α TMR, in our WT all-atom MDS (black) and in the cryo-EM structure (PDB: 6JXR) (green), related to Fig. **4B**. Magenta arrow indicates ζ_1I38 (**left**) or ζ_2I41 (**right**). **C** Normalised number of contacts of ζ_2 TMR with CD3γ TMR (**left**) and of CD3γ with ζ_2 TMR (**right**), related to Fig. **4C**. Comparison between protein-protein interactions resulted from our WT all-atom MDS (black) and the cryo-EM structure (PDB: 6JXR) (green). Magenta arrow indicates ζ_2I41. **D** Normalised number of contacts of ζ_1 with CD3ε (δε) TMR (**left**) and of CD3ε (δε) with ζ_1 TMR (**right**), related to Fig. **4D**. Comparison between protein-protein interactions resulted from our WT all-atom MDS (black) and the cryo-EM structure (PDB: 6JXR) (green). Magenta arrow indicates ζ_1I38. **E** Normalised number of interactions of ζ_1I38 with the rest of TCR-CD3 TMRs. Comparison between protein-protein interactions resulted from our WT all-atom MDS (black) and the cryo-EM structure (PDB: 6JXR) (green). **F** Normalised number of interactions of ζ_2I41 with the rest of TCR-CD3 TMRs. Comparison between protein-protein interactions resulted from our WT all-atom MDS (black) and the cryo-EM structure (PDB: 6JXR) (green). **G** Normalised number of contacts of ζ_1 and ζ_2 with α TMR (**left**) and of α TMR with ζζ TMR (**right**) in the WT (green) and ζA38 (red) all-atom MDS. Magenta arrow indicates ζ38 showing no significant contacts with α TMR in both WT and ζA38 simulations. However, one contact of ζ_2 (ζ_2Y33) with α TMR and two contacts of α TMR (αL247, αV249) with ζζ TMRs are reduced in the simulations carrying ζA38 substitution. For Figs. **S4G** - **S4N**, when comparing the contacts in the WT simulations to the contacts of the mutants, normalisation was done by dividing the number of contacts of each residue by the number of simulation frames. **H** Normalised number of contacts of ζ_1 and ζ_2 with β TMR (**left**) and of β TMR with ζζ TMR (**right**) in the WT (green) and ζA38 mutant (red) all-atom MDS. Magenta arrow indicates ζ38 showing no significant contacts with β TMR in both WT and ζA38 simulations. However, an increase in the interactions between ζ_2 (ζ_2F40) and β (βS276, βL280, βL284) were observed in the simulations carrying ζA38 substitution. This is likely to be the consequence of the increased ζζ loosening that allows ζ_2 to wobble and to come in contact with β TMR. **I** Normalised number of contacts of ζ_1 with CD3ε (δε) TMR (**left**) and of CD3ε (δε) with ζ_1 TMR (**right**) in the WT (green) and ζA38 mutant (red) all-atom MDS. Magenta arrow indicates $\zeta_1$38 showing that this residue reduced its interaction with CD3ε (δε) TMR when mutated to alanine (ζA38, in red), with a net effect of increasing flexibility of both ζ_1 and ζ_2 subunits ($\zeta_1 > \zeta_2$) relative to TCRαβ (see also Fig. **4F**). **J** Normalised number of contacts of α TMR (**left**) and of CD3ε (δε) TMR (**right**) with $\zeta_1$38 in the WT (green) and ζA38 mutant (red) all-atom MDS. A small number of contacts between $\zeta_1$38 and α TMR were observed in both WT and ζA38 simulations while all three residues of CD3ε (δε) TMR (εV134, εI135 and εI138) that interacted in the WT simulations reduced their contacts with ζ_1A38 (red). Normalisation is performed such that the number of contacts of TCRα TMR is compared to that of CD3ε (δε) residues. **K** Normalised number of contacts of ζ_1 and ζ_2 with α TMR (**left**) and of α TMR with ζζ TMR (**right**)

in the WT (green) and ζA41 mutant (red) all-atom MDS. Magenta arrow indicates ζ41 showing no significant contacts with α TMR in both WT and ζA41 simulations. However, one contact of ζ_2 (ζ_2Y33) with α TMR and two contacts of α TMR (αL247, αV249) with ζζ TMRs were reduced in the simulations carrying ζA41 substitution. However, one contact of ζ_2 (ζ_2R52) with α TMR increased on ζA41 substitution. **L** Normalised number of contacts of ζ_1 and ζ_2 with β TMR (**left**) and of β TMR with ζζ TMR (**right**) in the WT (green) and ζA41 mutant (red) all-atom MDS. Magenta arrow indicates ζ41 showing no significant contacts of $\zeta_1$41 and $\zeta_2$41 with β TMR in both WT and ζA41 simulations. However, two contacts of ζ_2 with β TMR (ζ_2L27 and ζ_2F40) and one of β TMR with ζζ TMRs (βL284) were increased in the simulations carrying ζA41 substitution. However, one residue of the β TMR (βS269) reduced contact with ζζ TMR during the ζA41 simulations. **M** Normalised number of contacts of ζ_2 with CD3γ TMR (**left**) and of CD3γ with ζ_2 TMR (**right**) in the WT (green) and ζA41 mutant (red) all-atom MDS. Magenta arrow indicates ζ41 showing that the mutated residue ζ_2A41 still maintained contact with CD3γ during the simulations compared to the WT. However, γV124, which strongly interacted with ζ_2I41 in the WT, reduced its interaction with ζ_2A41. One contact of ζ_2 (ζ_2R52) with CD3γ TMR increased in the simulations carrying the ζA41 substitution. **N** Normalised number of contacts of CD3γ TMR (**left**) and of β TMR (**right**) with $\zeta_2$41 in the WT (green) and ζA41 mutant (red) all-atom MDS. Two contacts of CD3γ (γV124 and γF127) with $\zeta_2$41 were reduced and one contact (γV128) was increased during ζA41 simulation. No significant contacts of β TMR with $\zeta_2$41 were observed in both WT and ζA41 simulations.

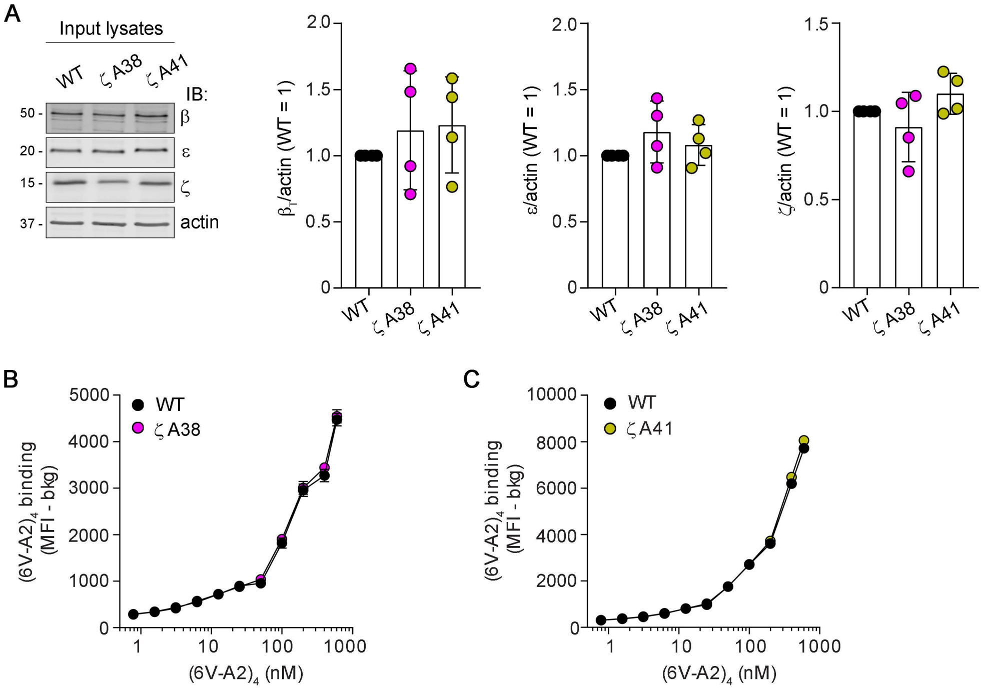

A
Input lysates
WT
ζ A38
ζ A41
IB:
50 -
20 -
15 -
37 -
β
ε
ζ
actin
$β_T$/actin (WT = 1)
ε/actin (WT = 1)
ζ/actin (WT = 1)
B
WT
ζ A38
$(6V\text{-}A2)_4$ binding (MFI - bkg)
$(6V\text{-}A2)_4$ (nM)
C
WT
ζ A41
$(6V\text{-}A2)_4$ binding (MFI - bkg)
$(6V\text{-}A2)_4$ (nM)

Fig S5

Figure S5. Loosening ζ association enhances signalling, related to Figure 5

A J76-1G4WT-ζKO expressing 1G4 ζWT or ζA38 or ζA41 were lysed with 0.5 % DDM and analysed by IB for β, ε, ζ and actin. **Left**, IB of input lysates of the experiment shown in Fig. **5B** (1 of 4 experiments). **Right**, mean ± SD of β_T/actin, ε/actin and ζ/actin normalized to WT, n = 4, unpaired *t*-test (ns). **B** $(6V\text{-}A2)_4$ binding to J76-1G4WT-ζKO expressing ζWT or ζA38 related to Fig. **5C**. Cells were induced with different doses of doxycycline, labelled or not with CellTrace violet, mixed 1:1 and stimulated for 3 min with different doses (0.78 – 600 nM) of PE-conjugated $(6V\text{-}A2)_4$ and analysed by FACS. Plot shows mean ± SD of 3 experiments measured in triplicates. **C** $(6V\text{-}A2)_4$ binding to J76-1G4WT-ζKO expressing ζWT or ζA41 related to Fig. **5D**. Cells were induced with different doses of doxycycline, labelled or not with CellTrace violet, mixed 1:1 and stimulated for 3 min with different doses (0.78 – 600 nM) of PE-conjugated $(6V\text{-}A2)_4$ and analysed by FACS. Plot shows mean ± SD of 3 experiments measured in triplicates.

A

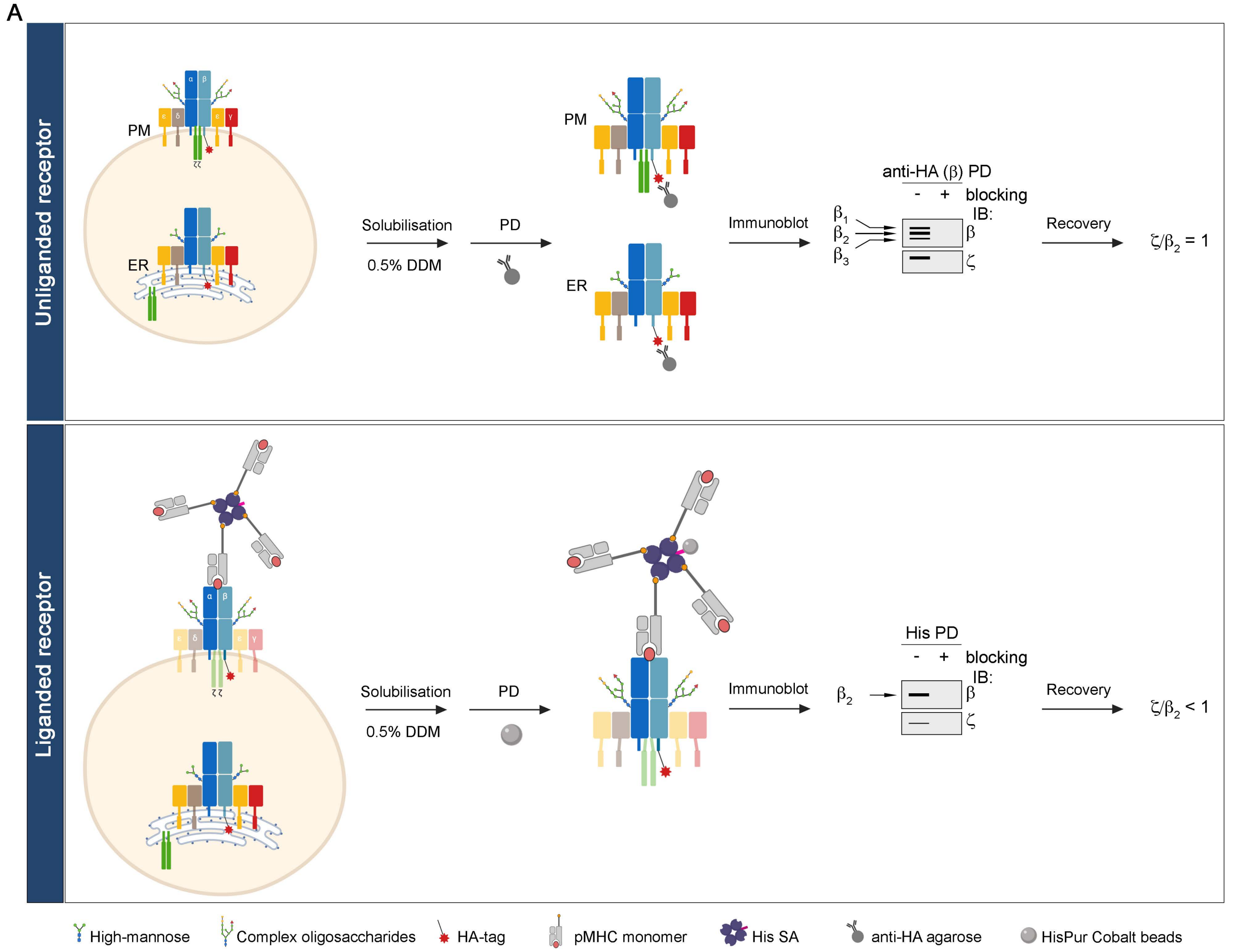

B

	K_d(nM)	CDR1α	CDR2α	CDR3α	CDR1β	CDR2β	CDR3β
1G4	1300	DRGSQS	IQSSQRE	CAVRPTSGGSYIPTFG	MNHEY	SVGAGI	CASSYVGNTGELFFG
QM-α	140	DRGSQS	IQS**W**QRE	CAVRPTSGGSYIPTFG	MNHEY	SV**AE**GI	CASSYVGNTG**LA**FFG
wtc51	15	DRGSQS	IQSSQRE	CAVRPTSGGSYIPTFG	MNHEY	SV**AIQT**	CASSYVGNTGELFFG

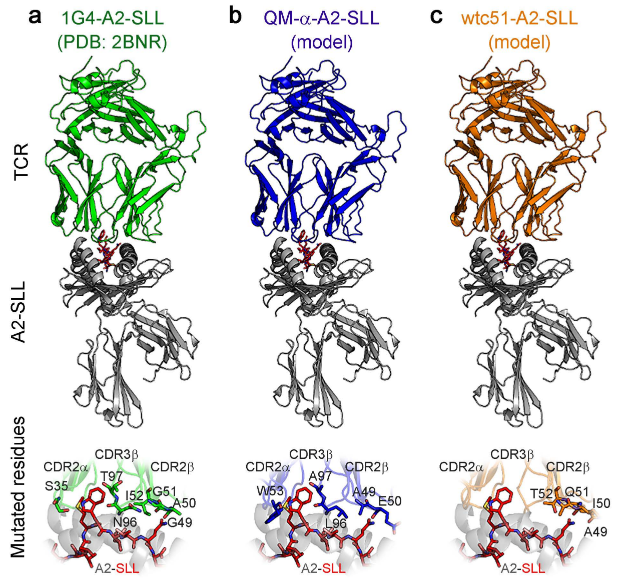

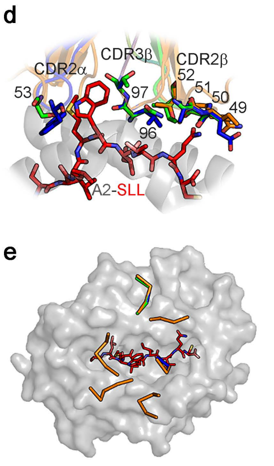

f

TCR	Crossing angle
1G4	69.4°
QM-α	69.4°
wtc51	69.4°

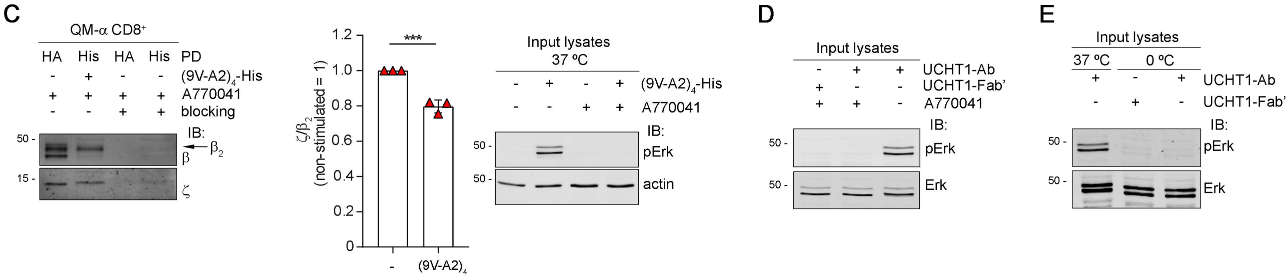

Fig S6

Figure S6. pMHC tetramer binding loosens αβ association with ζ, related to Figure 6

A Graphical scheme describing the experimental procedure used to compare the cohesion of unliganded receptor (**top**) and $(pMHC)_4$ ligated TCR-CD3 (liganded receptor, **bottom**). The unliganded receptor was captured by anti-HA (β-HA) PD described in Fig. **S2B**. To pull down the liganded receptor, cells were stimulated with tetramerised His-tagged streptavidin (His SA) and ligand excess was removed. Cells were solubilised with 0.5 % DDM and post-nuclear lysate was incubated with HisPur Cobalt beads to pull down $pMHC_4$-engaged TCR-CD3. The IB schemes on the right show expected bands pattern for β and ζ. Arrows indicate the different isoforms of TCRβ (β_1, β_2, β_3). To evaluate ζ recovery, ζ/β_2 ratio was calculated and the value for ζ/β_2 ratio from non-stimulated samples was set equal to one. This value represented the recovery of intact TCR-CD3 complex and was compared to ζ/β_2 ratio from $(pMHC)_4$ stimulated samples. A ratio < 1 indicates a lower recovery of ζ revealing a reduced cohesion of TCR-CD3 quaternary structure. See STAR Methods for a detailed description of the experimental procedure. **B** Structure and modelling of 1G4-WT and affinity enhanced 1G4 mutants (QM-α and wtc51) used in this study. **Top**, table shows binding affinities of the 1G4 and affinity enhanced TCR mutants (QM-α and wtc51) with sequence alignment highlighting the CDR loops with mutated residues (bold and underlined). **a**, structural overview of the 1G4 TCR (green)-A2 (grey)-SLL (red sticks) tri-molecular complex structure (PDB: 2BNR). Positions of the TCR residues (green sticks) in relation to A2-SLL that are mutated in the affinity enhanced TCRs are shown below. **b**, structural overview of the QM-α TCR (blue)-A2-SLL tri-molecular complex structure (mutations modelled using PDB: 2BNR). Positions of the TCR residues (blue sticks) in relation to A2-SLL that are mutated in the affinity enhanced TCR are shown below. **c**, structural overview of the wtc51 TCR (orange)-A2-SLL tri-molecular complex structure (mutations modelled using PDB: 2BNR). Positions of the TCR residues (orange sticks) in relation to A2-SLL that are mutated in the affinity enhanced TCRs are shown below. **d**, overlay of the mutated residues in the CDR loops comparing 1G4 (green sticks), QM-α (blue sticks) and wtc51 (orange sticks) TCRs. **e**, overlay of the positions of the CDR loops comparing 1G4 (green ribbon), QM-α (blue ribbon) and wtc51 (orange ribbon). **f**, analysis of the TCR crossing angles for each TCR. **C** J76 QM-α treated with A770041 and stimulated or not with $(9V\text{-}A2)_4$-His. **Left**, β-HA (lanes 1, 3) or His (lanes 2, 4) PD and IB for β and ζ (1 of 3 experiments). The arrow indicates β_2 isoform. **Middle**, mean ± SD of ζ/β_2, n = 3, unpaired t-test $p < 0.001$. **Right**, pErk IB: 1 of 3 experiments. **D** J76 1G4 ± A770041 were incubated with or w/o UCHT1-Fab' or UCHT1-Ab. pErk IB of the experiment shown in Fig. **6G** (1 of 3 experiments). **E** J76 1G4 were cooled on ice for 20 min and incubated for 5 min on ice with UCHT1-Fab' or UCHT1-Ab. pErk IB of the experiment shown in Fig. **6H** (1 of 3 experiments).

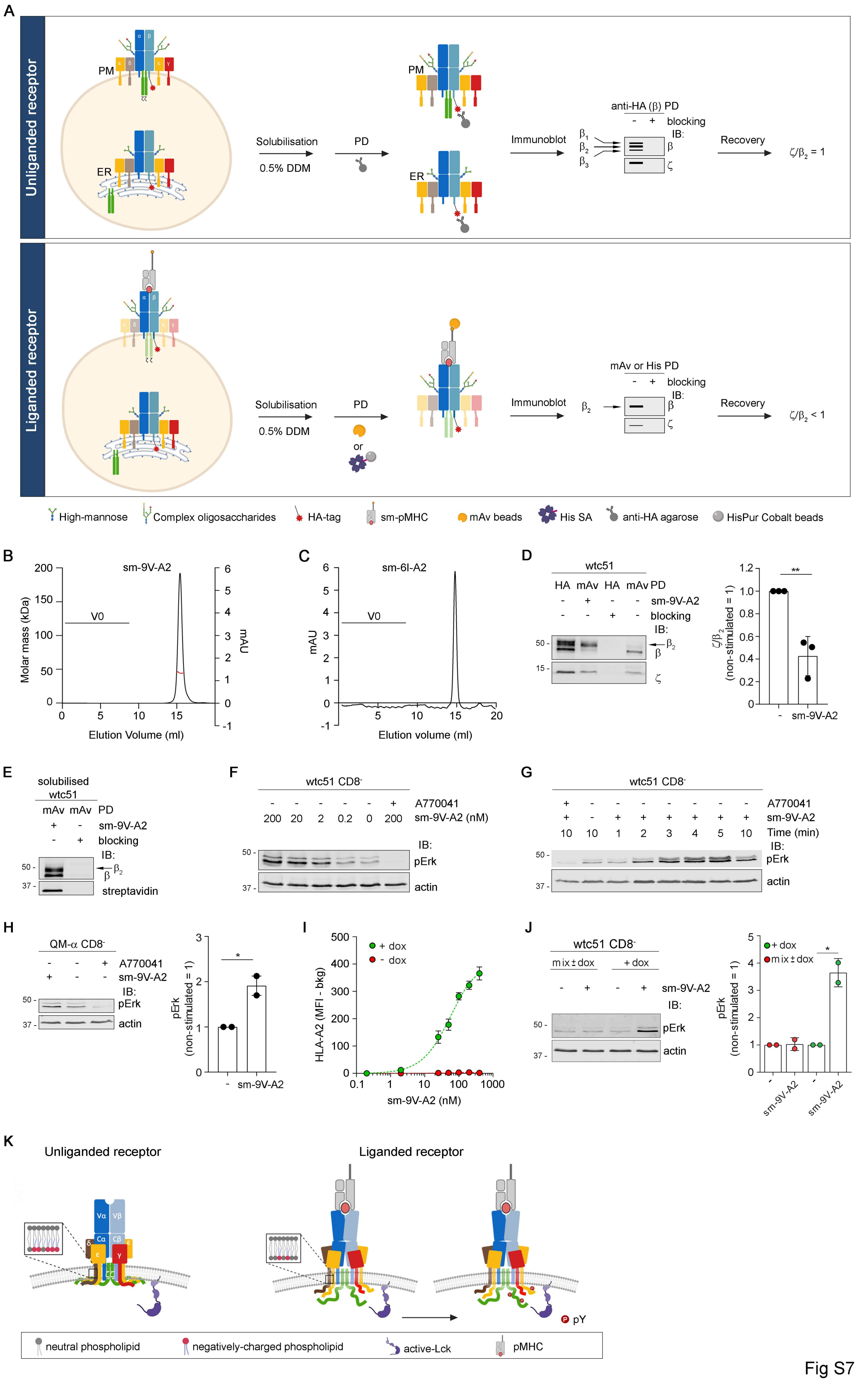

A
Unliganded receptor
Liganded receptor
Solubilisation
0.5% DDM
PD
Immunoblot
Recovery
anti-HA (β) PD
mAv or His PD
blocking
IB:
ζ/β2 = 1
ζ/β2 < 1
High-mannose
Complex oligosaccharides
HA-tag
sm-pMHC
mAv beads
His SA
anti-HA agarose
HisPur Cobalt beads
B
sm-9V-A2
Molar mass (kDa)
Elution Volume (ml)
mAU
C
sm-6I-A2
Elution volume (ml)
D
wtc51
sm-9V-A2
E
solubilised wtc51
streptavidin
F
wtc51 CD8-
A770041
sm-9V-A2 (nM)
pErk
actin
G
Time (min)
H
QM-α CD8-
pErk (non-stimulated = 1)
I
+ dox
- dox
HLA-A2 (MFI - bkg)
sm-9V-A2 (nM)
J
mix ± dox
+ dox
K
Unliganded receptor
Liganded receptor
pY
neutral phospholipid
negatively-charged phospholipid
active-Lck
pMHC

Fig S7

Figure S7. Monovalent pMHC in solution triggers TCR-CD3 untying and intracellular signalling, related to Figure 7

A Graphical scheme describing the experimental procedure used to compare the cohesion of unliganded receptor (**top**) and soluble, monovalent, mono-dispersed (sm)-pMHC ligated TCR-CD3 (liganded receptor, **bottom**). The unliganded receptor was captured by anti-HA (β-HA) PD described in Fig. **S2B**. To pull down the liganded receptor cells were stimulated with biotinylated sm-pMHC and ligand excess was removed. Cells were then solubilised with 0.5 % DDM and post-nuclear lysate was incubated with His-tagged streptavidin (His SA) followed by pull down with HisPur Cobalt beads or post-nuclear lysate was incubated with monomeric Avidin beads (mAv). The IB schemes on the right show expected bands pattern for β and ζ. Arrows on the left indicate the different isoforms of TCRβ (β_1, β_2, β_3). To evaluate ζ recovery, ζ/β_2 ratio was calculated and the value for ζ/β_2 ratio from the non-stimulated sample was set equal to one. This value represented the recovery of intact TCR-CD3 complex and was compared to ζ/β_2 ratio from sm-pMHC stimulated samples. A ratio < 1 indicates a lower recovery of ζ revealing a reduced cohesion of TCR-CD3 quaternary structure. See STAR Methods for a detailed description of the experimental procedure. **B** Size-exclusion chromatography-multi-angle-light scattering analysis of sm-9V-A2 showing a single, homogeneous peak of 44.6 kDa. The red line indicates the Molar mass (kDa). V0: void volume. **C** Gel-filtration chromatogram of sm-6I-A2 showing a single, homogeneous peak. V0: void volume. **D** J76 wtc51 stimulated or not with sm-9V-A2. **Left**, β-HA (lanes 1, 3) or monomeric Avidin (mAv) (lanes 2, 4) PD and IB for β and ζ (1 of 3 experiments). The arrow indicates β_2 isoform. **Right**, mean ± SD of ζ/β_2, n = 3, unpaired *t*-test $p < 0.01$. **E** $CD8^-$ J76 wtc51 were lysed, incubated or not with sm-9V-A2 and subjected to PD by anti-HA or monomeric Avidin (mAv). Monomeric Avidin (mAv) PD and IB for β and streptavidin IRDye 800CW. The arrow indicates β_2 isoform. **F** $CD8^-$ J76 wtc51 ± A770041 were stimulated or not with the indicated concentrations of sm-9V-A2 for 5 minutes. pErk IB representative of 2 experiments. **G** $CD8^-$ J76 wtc51 ± A770041 were stimulated or not with sm-9V-A2 for the indicated time points. pErk IB representative of 2 experiments. **H** $CD8^-$ J76 QM-α ± A770041 were stimulated or not with sm-9V-A2. **Left**, pErk IB (1 of 2 experiments). **Right**, mean ± SEM of pErk, n = 2, unpaired *t*-test $p = 0.05$. **I** Sm-9V-A2 binding to $CD8^-$ J76 wtc51. Cells were induced or not with doxycycline (dox), labelled with CellTrace violet or left untreated, mixed 1:1 and stimulated for 5 min with different doses (0.02 – 400 nM) of sm-9V-A2 at 37 ºC. Cells were rapidly washed with FACS buffer, stained with anti-HLA-A2 and analysed by FACS. Non-linear regression fit of sm-9V-A2 (nM) vs. HLA-A2 (MFI), n = 3. **J** $CD8^-$ J76 wtc51 not doxycycline-induced (-dox) for TCR expression were reacted or not with 200 nM sm-9V-A2 for 5 minutes at 37 ºC, washed and mixed with equal number of dox-induced $CD8^-$ J76 wtc51 (mix ± dox), see STAR Methods for a detailed protocol. Dox-induced (+ dox) $CD8^-$ J76 wtc51 stimulated or not with 200 nM sm-9V-A2 for 5 minutes at 37 ºC served as control. **Left**, pErk IB (1 of 2 experiments). **Right**, mean ± SD of pErk of mixed dox-induced and not dox-induced ± sm-9V-A2 cells (mix ± dox), n = 2 experiments in duplicates, unpaired *t*-test: ns (mix ± dox), $p < 0.05$ (+ dox). **K** Graphical scheme describing the "TCR-CD3 allosteric relaxation" mechanism uncovered in this study. Briefly we suggest that in absence of force, co-receptor, clustering or PTPs exclusion, monovalent pMHC binding to TCR-CD3 allosterically regulates a cascade of conformational changes that relaxes the quaternary structure of TCR-CD3 TMRs. In the proposed model, conformational changes occurring at the pMHC binding site propagate to CαCβ ECDs at the site where they contact the CD3 subunits. These rearrangements are

transmitted to the CD3 TMRs, resulting in a reduced cohesion of TCR-CD3 TMRs, ITAMs exposure and their phosphorylation by active Lck. Moreover, we envisage the possibility that pMHC-induced reconfiguration of the octamer's TMRs leads to a local redistribution of negatively-charged lipids which could reduce hydrophobic and electrostatic forces that help holding CD3 tails within the plasma membrane.

Table S1. 1G4 amino acid sequence, related to Figure S1A and to the STAR methods section

1G4 WT αβ self-cleavable single polypeptide (TCRβ-spacer-HA-spacer-F2A-L1-TCRα-L2-Flag)
MSIGLLCCAALSLLWAGPVNAGVTQTPKFQVLKTGQSMTLQCAQDMNHEYMSWYRQDPGMGLRLIHYSVG<br>AGITDQGEVPNGYNVSRSTTEDFPLRLLSAAPSQTSVYFCASSYVGNTGELFFGEGSRLTVLEDLKNVFPPEVA<br>VFEPSEAEISHTQKATLVCLATGFYPDHVELSWWVNGKEVHSGVSTDPQPLKEQPALNDSRYCLSSRLRVSA<br>TFWQNPRNHFRCQVQFYGLSENDEWTQDRAKPVTQIVSAEAWGRADCGFTSESYQQGVLSATILYEILLGKA<br>TLYAVLVSALVLMAMVKRKDFSRGGSGGGSGGGSGGGSGGGSGASYPYDVPDYAGAGGSGGGSGGGSGG<br>GSGGGSGVKQTLNFDLLKLAGDVESNPGPEFMETLLGLLILWLQLQWVSSKQEVTQIPAALSVPEGENLVLN<br>CSFTDSAIYNLQWFRQDPGKGLTSLLLIQSSQREQTSGRLNASLDKSSGRSTLYIAASQPGDSATYLCAVRPTS<br>GGSYIPTFGRGTSLIVHPYIQNPDPAVYQLRDSKSSDKSVCLFTDFDSQTNVSQSKDSDVYITDKTVLDMRSM<br>DFKSNSAVAWSNKSDFACANAFNNSIIPEDTFFPSPESSCDVKLVEKSFETDTNLNFQNLSVIGFRILLLKVAGF<br>NLLMTLRLWSSSGDYKDDDDK

Table S2. gRNA and Primers, related to the STAR methods section

CRISPR gRNA	
CD3ζ	CAGGCACAGTTGCCGATTACAGG
Gateway cloning	
1G4 β-HA-F2A-α-FLAG FW	GGGGACAAGTTTGTACAAAAAAGCAGGCTTAATGAGCATCGGCCT CCTGTGCTGTGCAGCC
1G4 β-HA-F2A-α-FLAG REV	GGGGACCACTTTGTACAAGAAAGCTGGGTTTTATTTGTCG TCGTCGTCTTTGTAGTCTCC
CD3ζ-TST FW	ACAAGTTTGTACAAAAAAGCAGGCTTCACCATGAAGTGGAAGGCG CTTTTCAC
CD3ζ-TST REV	ACCACTTTGTACAAGAAAGCTGGGTTTTATTTTTCGAACTGCGGGT GGCTC
Amplification of 1G4 WT from cDNA	
TCRα FW	GGATCCATGGAGACCCTCTTGGGCCTGCTT
TCRα REV	TCTAGAGCTGGACCACAGCCGCAGCGT
TCRβ FW	GGATCCATGAGCATCGGCCTCCTGTGCTGT
TCRβ REV	GGATCCATGAGCATCGGCCTCCTGTGCTGT

Table S3. Protein sequences used in the all atom simulations, related to to the STAR methods section

Chain	Residue range	TMR sequences used
CD3-ζ_1	27-57	LDPKLCYLLDGILFIYGVILTALFLRVKFSR
CD3-ζ_2	27-55	LDPKLCYLLDGILFIYGVILTALFLRVKF
TCR-α	239-273	DTNLNFQNLSVIGFRILLLKVAGFNLLMTLRLWSS
TCR-β	266-307	TSESYQQGVLSATILYEILLGKATLYAVLVSALVLMAMVKRK
CD3-δ	98-129	ELDPATVAGIIVTDVIATLLLALGVFCFAGHE
CD3-ε (δε dimer)	125-155	MDVMSVATIVIVDICITGGLLLLVYYWSKNR
CD3-γ	109-138	ELNAATISGFLFAEIVSIFVLAVGVYFIAG
CD3-ε (γε dimer)	125-156	MDVMSVATIVIVDICITGGLLLLVYYWSKNRK

Table S4. Bilayer lipid concentrations used in the all atom simulations, related to the STAR methods section

Lipid concentration (%)	POPC	POPS	POPE	SM	CHOL	PIP_2	PIP_3
Inner leaflet	10	20	40	-	20	8	2
Outer leaflet	50	-	10	20	20	-	-
